# Supplementary material for: Prevalence of hepatitis B virus infection and its associated factors among students in N’Djamena, Chad
Source: PLoS One. 2024 Apr 18;19(4):e0273589. doi: 10.1371/journal.pone.0273589 (PMC11025733; doi:10.1371/journal.pone.0273589)
Supplement: S1 Questionnaire — (DOCX) [file pone.0273589.s002.docx]

**Questionnaire used in the study**

Faculty/University of ……..

Pre-test counseling

1-Code: TC XX…… Level of study (year)………Age… ……city of origin……………...

2-Civil status …………………. Sexual partners: one □ several □ Gender…… Ethnicity……...

3-Do you know about hepatitis B virus infection? Yes □ No□

4-Have you ever taken the screening test? Yes □ No □ If yes results: HBsAg Pos □ Neg □

5- Have you been transfused? Yes □ No □ If yes in which year?..................................

6-Have you ever had surgery? Yes □ No□

7-Have you ever been hospitalized for a long time? Yes □ No□

8-Do you have a HBV carrier mother? Yes □ No □ ignorant □

9-Do you have a close family member carrier of HBV? Yes □ No □ ignorant □

10-Have you had transcutaneous medical examinations or acupuncture? Yes □ No□

11-Have you had a piercing, tattoo, circumcision, excision or scarification? If yes, specify which of the practices………………………Yes□ No□

12-Have you been imprisoned at some point in your life? Yes□ No□.

13-Have you had unprotected sex? Yes□ No□

14-Have you ever taken drugs intravenously? Yes□ No□

15-Do you drink alcohol? Yes□ No □ No response □

16-Do you share sharp objects? Yes□ No □ No answer□

17-Do you live with your family? Alone □ with a roommate □ No answer □

18-What is the living density? 1 or 2 people/room□ 2 people or more per room□ No answer □

19-Do you share clothes? Yes□ No □ No response □

20-Do you share sleeping space? Yes□ No □ No response □

21-Are you ready to accept a positive result? Yes□ No □ No answer □

22- Adherence to screening? Yes□ No□

23- Test results Rapid test: HBsAg Pos □ Neg □

24-Return for results: Yes□ No □

Post-test counseling

Date……………counsellor …………………Additional counselling? Yes □ No □ Appointment date for clinical assessment: ……....Observations: ………………… …………
