# Supplementary material for: Prevalence of hepatitis B virus infection and its associated factors among students in N’Djamena, Chad
Source: PLoS One. 2024 Apr 18;19(4):e0273589. doi: 10.1371/journal.pone.0273589 (PMC11025733; doi:10.1371/journal.pone.0273589)
Supplement: S1 File — (DOCX) [file pone.0273589.s003.docx]

**Analysis results**

rm (list = ls ())
library(funModeling)

## Warning: le package 'funModeling' a été compilé avec la version R 4.2.2

## Le chargement a nécessité le package : Hmisc

## Warning: le package 'Hmisc' a été compilé avec la version R 4.2.2

## Le chargement a nécessité le package : lattice

## Le chargement a nécessité le package : survival

## Le chargement a nécessité le package : Formula

## Le chargement a nécessité le package : ggplot2

## Warning: le package 'ggplot2' a été compilé avec la version R 4.2.2

##
## Attachement du package : 'Hmisc'

## Les objets suivants sont masqués depuis 'package:base':
##
## format.pval, units

## funModeling v.1.9.4 :)
## Examples and tutorials at livebook.datascienceheroes.com
## / Now in Spanish: librovivodecienciadedatos.ai

library(finalfit)

## Warning: le package 'finalfit' a été compilé avec la version R 4.2.2

library(readxl)

## Warning: le package 'readxl' a été compilé avec la version R 4.2.2

library(readxl)
library(dplyr)

## Warning: le package 'dplyr' a été compilé avec la version R 4.2.2

##
## Attachement du package : 'dplyr'

## Les objets suivants sont masqués depuis 'package:Hmisc':
##
## src, summarize

## Les objets suivants sont masqués depuis 'package:stats':
##
## filter, lag

## Les objets suivants sont masqués depuis 'package:base':
##
## intersect, setdiff, setequal, union

library(tidyverse)

## Warning: le package 'tidyverse' a été compilé avec la version R 4.2.2

## ── Attaching packages
## ───────────────────────────────────────
## tidyverse 1.3.2 ──

## ✔ tibble 3.1.8 ✔ purrr 0.3.5
## ✔ tidyr 1.2.1 ✔ stringr 1.4.1
## ✔ readr 2.1.3 ✔ forcats 0.5.2

## Warning: le package 'tibble' a été compilé avec la version R 4.2.2

## Warning: le package 'tidyr' a été compilé avec la version R 4.2.2

## Warning: le package 'readr' a été compilé avec la version R 4.2.2

## Warning: le package 'purrr' a été compilé avec la version R 4.2.2

## Warning: le package 'stringr' a été compilé avec la version R 4.2.2

## Warning: le package 'forcats' a été compilé avec la version R 4.2.2

## ── Conflicts ────────────────────────────────────────── tidyverse_conflicts() ──
## ✖ dplyr::filter() masks stats::filter()
## ✖ dplyr::lag() masks stats::lag()
## ✖ dplyr::src() masks Hmisc::src()
## ✖ dplyr::summarize() masks Hmisc::summarize()

library("PerformanceAnalytics")

## Warning: le package 'PerformanceAnalytics' a été compilé avec la version R 4.2.2

## Le chargement a nécessité le package : xts

## Warning: le package 'xts' a été compilé avec la version R 4.2.2

## Le chargement a nécessité le package : zoo

## Warning: le package 'zoo' a été compilé avec la version R 4.2.2

##
## Attachement du package : 'zoo'
##
## Les objets suivants sont masqués depuis 'package:base':
##
## as.Date, as.Date.numeric
##
##
## Attachement du package : 'xts'
##
## Les objets suivants sont masqués depuis 'package:dplyr':
##
## first, last
##
##
## Attachement du package : 'PerformanceAnalytics'
##
## L'objet suivant est masqué depuis 'package:graphics':
##
## legend

library(finalfit)
library(mlbench)

## Warning: le package 'mlbench' a été compilé avec la version R 4.2.2

library(forestmodel)

## Warning: le package 'forestmodel' a été compilé avec la version R 4.2.2

library(knitr)
library(ggplot2)
library(gtsummary)

## Warning: le package 'gtsummary' a été compilé avec la version R 4.2.2

library(car)

## Warning: le package 'car' a été compilé avec la version R 4.2.2

## Le chargement a nécessité le package : carData
##
## Attachement du package : 'car'
##
## L'objet suivant est masqué depuis 'package:purrr':
##
## some
##
## L'objet suivant est masqué depuis 'package:dplyr':
##
## recode

library(funModeling)
library(MASS)

## Warning: le package 'MASS' a été compilé avec la version R 4.2.2

##
## Attachement du package : 'MASS'
##
## L'objet suivant est masqué depuis 'package:gtsummary':
##
## select
##
## L'objet suivant est masqué depuis 'package:dplyr':
##
## select

BDE <- read_excel("C:/Users/HP/Desktop/BDE.xlsx")

tbl <- BDE %>%
 tbl_summary(
 include = c("Genre",
 "Age",
 "Age_group",
 "Site",
 "Level",
 "Marital_Status" ,
 "History_of_surgical_procedure" ,
 "History_of_blood_transfusion" ,
 "Number_Sexual_partenar" ,
 "Knowledge_of_HepatitisB" ,
 "History_of_hospital_admission" ,
 "Mother_HBV" ,
 "Family_history_HBV" ,
 "Transcutaneous_medical_examinations" ,
 "History_of_injection_of_drugs" ,
 "Risky_practice",
 "Jail" ,
 "Unprotected_sex" ,
 "Sharing_of_sharp_materials" ,
 "Type_of_housing" ,
 "Housing_density" ,
 "Shared_bedding",
 "History_of_HBV_screening"
 )
 , by = HBsAg_status
 ) %>%
 add_overall() %>%
 add_p()
tbl

## Table printed with `knitr::kable()`, not {gt}. Learn why at
## https://www.danieldsjoberg.com/gtsummary/articles/rmarkdown.html
## To suppress this message, include `message = FALSE` in the code chunk header.

| **Characteristic** | **Overall**, N = 457 | **0**, N = 389 | **1**, N = 68 | **p-value** |
| --- | --- | --- | --- | --- |
| Genre |  |  |  | >0.9 |
| Femal | 109 (24%) | 93 (24%) | 16 (24%) |  |
| Male | 348 (76%) | 296 (76%) | 52 (76%) |  |
| Age | 24.0 (22.0, 25.0) | 24.0 (22.0, 26.0) | 23.5 (22.0, 25.0) | 0.2 |
| Age_group |  |  |  | 0.13 |
| less_25 | 343 (75%) | 287 (74%) | 56 (82%) |  |
| more_26 | 114 (25%) | 102 (26%) | 12 (18%) |  |
| Site |  |  |  | 0.004 |
| FLLAC | 75 (16%) | 54 (14%) | 21 (31%) |  |
| FSE | 99 (22%) | 86 (22%) | 13 (19%) |  |
| FSEA | 50 (11%) | 45 (12%) | 5 (7.4%) |  |
| FSEG | 100 (22%) | 92 (24%) | 8 (12%) |  |
| FSHS | 83 (18%) | 67 (17%) | 16 (24%) |  |
| UEK | 50 (11%) | 45 (12%) | 5 (7.4%) |  |
| Level |  |  |  | 0.2 |
| Bachelor1 | 141 (31%) | 113 (29%) | 28 (41%) |  |
| Bachelor2 | 143 (31%) | 123 (32%) | 20 (29%) |  |
| Bachelor3 | 168 (37%) | 148 (38%) | 20 (29%) |  |
| Master & PhD | 5 (1.1%) | 5 (1.3%) | 0 (0%) |  |
| Marital_Status |  |  |  | 0.8 |
| Maried | 109 (24%) | 92 (24%) | 17 (25%) |  |
| Single | 348 (76%) | 297 (76%) | 51 (75%) |  |
| History_of_surgical_procedure | 22 (4.8%) | 22 (5.7%) | 0 (0%) | 0.058 |
| History_of_blood_transfusion | 58 (13%) | 48 (12%) | 10 (15%) | 0.6 |
| Number_Sexual_partenar |  |  |  | 0.7 |
| more than one | 252 (55%) | 213 (55%) | 39 (57%) |  |
| one | 205 (45%) | 176 (45%) | 29 (43%) |  |
| Knowledge_of_HepatitisB | 248 (54%) | 210 (54%) | 38 (56%) | 0.8 |
| History_of_hospital_admission | 108 (24%) | 92 (24%) | 16 (24%) | >0.9 |
| Mother_HBV | 102 (22%) | 79 (20%) | 23 (34%) | 0.014 |
| Family_history_HBV |  |  |  | 0.3 |
| Ignore | 108 (24%) | 92 (24%) | 16 (24%) |  |
| no | 278 (61%) | 241 (62%) | 37 (54%) |  |
| yes | 71 (16%) | 56 (14%) | 15 (22%) |  |
| Transcutaneous_medical_examinations | 65 (14%) | 47 (12%) | 18 (26%) | 0.002 |
| History_of_injection_of_drugs | 15 (3.3%) | 14 (3.6%) | 1 (1.5%) | 0.7 |
| Risky_practice | 136 (30%) | 115 (30%) | 21 (31%) | 0.8 |
| Jail | 28 (6.1%) | 23 (5.9%) | 5 (7.4%) | 0.6 |
| Unprotected_sex | 297 (65%) | 253 (65%) | 44 (65%) | >0.9 |
| Sharing_of_sharp_materials | 186 (41%) | 155 (40%) | 31 (46%) | 0.4 |
| Type_of_housing |  |  |  | 0.004 |
| alone | 51 (11%) | 48 (12%) | 3 (4.4%) |  |
| Family | 353 (77%) | 303 (78%) | 50 (74%) |  |
| Housing_density |  |  |  | 0.5 |
| 1-2 | 212 (46%) | 178 (46%) | 34 (50%) |  |
| more than two | 245 (54%) | 211 (54%) | 34 (50%) |  |
| Shared_bedding | 280 (61%) | 240 (62%) | 40 (59%) | 0.7 |
| History_of_HBV_screening | 107 (23%) | 82 (21%) | 25 (37%) | 0.005 |

dependent = "HBsAg_status"
explanatory_full = c("Genre",
 "Age_group",
 "Marital_Status",
 "History_of_surgical_procedure",
 "History_of_blood_transfusion" ,
 "Number_Sexual_partenar",
 "Knowledge_of_HepatitisB",
 "History_of_hospital_admission",
 "Mother_HBV",
 "History_of_injection_of_drugs",
 "Jail",
 "Unprotected_sex" ,
 "Transcutaneous_medical_examinations",
 "Sharing_of_sharp_materials" ,
 "Type_of_housing",
 "Housing_density")

glm_full <- glm( HBsAg_status ~ Genre+
 Age_group +
 Marital_Status +
 History_of_surgical_procedure +
 History_of_blood_transfusion+
 Number_Sexual_partenar +
 Knowledge_of_HepatitisB+
 Number_Sexual_partenar +
 Knowledge_of_HepatitisB+
 History_of_hospital_admission +
 Mother_HBV +
 Transcutaneous_medical_examinations +
 Jail +
 History_of_injection_of_drugs +
 Unprotected_sex +
 Sharing_of_sharp_materials +
 Type_of_housing +
 Housing_density +
 Alcoolism+
 Share_clothing,
 data=BDE, family=binomial)

glm_multi_restreint <- stepAIC(glm_full, trace=FALSE)

Anova(glm_multi_restreint)

## Response: HBsAg_status
## LR Chisq Df Pr(>Chisq)
## Age_group 3.9793 1 0.046063 *
## History_of_surgical_procedure 6.7330 1 0.009464 **
## Mother_HBV 4.7907 1 0.028613 *
## Transcutaneous_medical_examinations 9.0142 1 0.002679 **
## History_of_injection_of_drugs 2.0211 1 0.155127
## Type_of_housing 6.2552 1 0.012383 *
## ---
## Signif. codes: 0 '***' 0.001 '**' 0.01 '*' 0.05 '.' 0.1 ' ' 1

explanatory_final = c("Age_group",
 "Marital_Status",
 "Knowledge_of_HepatitisB",
 "History_of_injection_of_drugs",
 "Mother_HBV",
 "Transcutaneous_medical_examinations",
 "Type_of_housing",
 "Housing_density")
res_summary <- BDE %>%
 summary_factorlist(dependent, explanatory_full, fit_id=TRUE)

res_uni <- BDE %>%
 glmmulti(dependent, explanatory_full) %>%
 fit2df(estimate_suffix="(COR)")

res_multi_full <- BDE %>%
 glmmulti(dependent, explanatory_full) %>%
 fit2df(estimate_suffix="(complet)")

res_glm_uni_multi <- BDE %>%
 finalfit(dependent, explanatory_full)

res_multi_final <- BDE %>%
 glmmulti(dependent, explanatory_final) %>%
 fit2df(estimate_suffix="(AOR)")

## Waiting for profiling to be done...

tab_res <- res_summary %>%
 finalfit_merge(res_uni) %>%
 finalfit_merge(res_multi_full) %>%
 finalfit_merge(res_multi_final) %>%
 dplyr::select(-levels, fit_id, -index)
knitr::kable(tab_res, row.names=FALSE, align=c("l", "l", "r", "r", "r", "r", "r"))

| fit_id | label | 0 | 1 | OR(COR) | OR(complet) | OR(AOR) |
| --- | --- | --- | --- | --- | --- | --- |
| GenreFemal | Genre | 93 (23.9) | 16 (23.5) | - | - | - |
| GenreMale |  | 296 (76.1) | 52 (76.5) | 1.09 (0.58-2.16, p=0.792) | 1.09 (0.58-2.16, p=0.792) | - |
| Age_group less_25 | Age_group | 287 (73.8) | 56 (82.4) | - | - | - |
| Age_group more_26 |  | 102 (26.2) | 12 (17.6) | 0.39 (0.17-0.82, p=0.018) | 0.39 (0.17-0.82, p=0.018) | 0.41 (0.18-0.85, p=0.022) |
| Marital_Status Maried | Marital_Status | 92 (23.7) | 17 (25.0) | - | - | - |
| Marital_Status Single |  | 297 (76.3) | 51 (75.0) | 0.58 (0.28-1.22, p=0.146) | 0.58 (0.28-1.22, p=0.146) | 0.65 (0.33-1.31, p=0.219) |
| History_of_surgical_procedure no | History_of_surgical_procedure | 367 (94.3) | 68 (100.0) | - | - | - |
| History_of_surgical_procedure yes |  | 22 (5.7) |  | 0.00 (0.00-1597502.09, p=0.984) | 0.00 (0.00-1597502.09, p=0.984) | - |
| History_of_blood_transfusion no | History_of_blood_transfusion | 341 (87.7) | 58 (85.3) | - | - | - |
| History_of_blood_transfusion yes |  | 48 (12.3) | 10 (14.7) | 1.50 (0.64-3.28, p=0.327) | 1.50 (0.64-3.28, p=0.327) | - |
| Number_Sexual_partenarmore than one | Number_Sexual_partenar | 213 (54.8) | 39 (57.4) | - | - | - |
| Number_Sexual_partenar one |  | 176 (45.2) | 29 (42.6) | 0.96 (0.53-1.73, p=0.896) | 0.96 (0.53-1.73, p=0.896) | - |
| Knowledge_of_HepatitisB no | Knowledge_of_HepatitisB | 179 (46.0) | 30 (44.1) | - | - | - |
| Knowledge_of_HepatitisB yes |  | 210 (54.0) | 38 (55.9) | 1.49 (0.83-2.70, p=0.186) | 1.49 (0.83-2.70, p=0.186) | 1.49 (0.84-2.66, p=0.172) |
| History_of_hospital_admission no | History_of_hospital_admission | 297 (76.3) | 52 (76.5) | - | - | - |
| History_of_hospital_admission yes |  | 92 (23.7) | 16 (23.5) | 1.07 (0.55-2.03, p=0.830) | 1.07 (0.55-2.03, p=0.830) | - |
| Mother_HBV no | Mother_HBV | 310 (79.7) | 45 (66.2) | - | - | - |
| Mother_HBV yes |  | 79 (20.3) | 23 (33.8) | 2.02 (1.09-3.71, p=0.024) | 2.02 (1.09-3.71, p=0.024) | 2.11 (1.16-3.78, p=0.013) |
| History_of_injection_of_drugs no | History_of_injection_of_drugs | 375 (96.4) | 67 (98.5) | - | - | - |
| History_of_injection_of_drugs yes |  | 14 (3.6) | 1 (1.5) | 0.27 (0.01-1.60, p=0.237) | 0.27 (0.01-1.60, p=0.237) | 0.29 (0.02-1.59, p=0.244) |
| Jail no | Jail | 366 (94.1) | 63 (92.6) | - | - | - |
| Jailyes |  | 23 (5.9) | 5 (7.4) | 0.85 (0.24-2.51, p=0.786) | 0.85 (0.24-2.51, p=0.786) | - |
| Unprotected_sex no | Unprotected_sex | 136 (35.0) | 24 (35.3) | - | - | - |
| Unprotected_sex yes |  | 253 (65.0) | 44 (64.7) | 0.95 (0.52-1.76, p=0.859) | 0.95 (0.52-1.76, p=0.859) | - |
| Transcutaneous_medical_examinations no | Transcutaneous_medical_examinations | 342 (87.9) | 50 (73.5) | - | - | - |
| Transcutaneous_medical_examinations yes |  | 47 (12.1) | 18 (26.5) | 3.13 (1.53-6.31, p=0.001) | 3.13 (1.53-6.31, p=0.001) | 2.97 (1.51-5.76, p=0.001) |
| Sharing_of_sharp_materials no | Sharing_of_sharp_materials | 234 (60.2) | 37 (54.4) | - | - | - |
| Sharing_of_sharp_materials yes |  | 155 (39.8) | 31 (45.6) | 1.32 (0.75-2.31, p=0.337) | 1.32 (0.75-2.31, p=0.337) | - |
| Type_of_housing alone | Type_of_housing | 48 (12.3) | 3 (4.4) | - | - | - |
| Type_of_housing Family |  | 341 (87.7) | 65 (95.6) | 4.49 (1.43-20.16, p=0.022) | 4.49 (1.43-20.16, p=0.022) | 4.63 (1.50-20.52, p=0.018) |
| Housing_density1-2 | Housing_density | 178 (45.8) | 34 (50.0) | 1.48 (0.82-2.65, p=0.192) | 1.48 (0.82-2.65, p=0.192) | 1.45 (0.81-2.58, p=0.210) |
| Housing_density>2 |  | 211 (54.2) | 34 (50.0) | - | - | - |
