# Supplementary material for: Prevalence of hepatitis B virus infection and its associated factors among students in N’Djamena, Chad
Source: PLoS One. 2024 Apr 18;19(4):e0273589. doi: 10.1371/journal.pone.0273589 (PMC11025733; doi:10.1371/journal.pone.0273589)
Supplement: Attachment — Submitted filename: PONE-D-22-22361-annotated.pdf [file pone.0273589.s006.pdf]

PLOS ONE

# Prevalence of hepatitis B virus infection and associated risk factors among students in N'Djamena, Chad

--Manuscript Draft--

| | |
|---|---|
| **Manuscript Number:** | PONE-D-22-22361 |
| **Article Type:** | Research Article |
| **Full Title:** | Prevalence of hepatitis B virus infection and associated risk factors among students in N'Djamena, Chad |
| **Short Title:** | Hepatitis B virus and associated risk factors among Chadian studens |
| **Corresponding Author:** | NALDA DEBSIKREO<br>Cheikh Anta Diop University of Dakar: Universite Cheikh Anta Diop de Dakar<br>Dakar, SENEGAL |
| **Keywords:** | hepatitis B; seroprevalence; student; Chad |
| **Abstract:** | Background:<br>Viral hepatitis B (HBV) is a major public health threat. In Chad, HBV screening is not systematic, due to the low interest of providers or frequent shortages of supplies. Thus, most people at risk such as students are unaware of their status. However, many persons chronically infected with HBV have no or low liver disease. This study aims to determine the prevalence of HBV and identify risk factors among students in N'Djamena, Chad.<br>Methods:<br>A cross-sectional study was conducted in July 2021 including 457 students randomly selected within the universities of N'Djamena and Emi Koussi. A structured questionnaire was used to collect data on sociodemographic characteristics and other risk factors. HBsAg testing was performed using the Determine HBsAg® Rapid Test Kit. Descriptive analyzes and binary logistic regressions were used to examine the relationship between dependent variables and sociodemographic including other risk factors. Odds ratios (ORs) and their 95% confidence intervals (CIs) were used as indicators of the association. A p value of 0.05 was used to indicate the statistical significance of the relationships.<br>Results:<br>The HBsAg seroprevalence was 14.87%. Students aged between 26-35 years (0R= 0.90, 95% CI: 0.42-1.80; p= 0.032), who were born from HBsAg positive mothers (OR= 0.48, 95% CI: 0.08-3.84; p = 0.027), who lived with a roommate (OR= 5.42, 95% CI: 1.55-25.6; p= 0.025) or with family (OR= 2.69, 95%CI: 0.90-11.7; p= 0.025) or had a history of hepatitis (OR= 0.46, 95% CI : 0.25-0.86, p=0.015) were less likely to contract HBV infection.<br>Conclusion:<br>The prevalence of hepatitis B is high among Chadian students. There is an urgent need to provide screening tests, HBV vaccination to improve the living conditions for students in Chad. This study highlights some solutions to improve the situation of hepatitis B in Chad and suggests policies to eliminate viral hepatitis. |
| **Order of Authors:** | NALDA DEBSIKREO |
| | Birwé Léon Mankréo |
| | Azoukalné Moukénet |
| | Merwa Ouangkake |
| | Nathan Mara |
| | Ali Mahamat Moussa |
| | Ndèye Coumba Toure-Kane |
| | Françoise Lunel-Fabiani |
| **Opposed Reviewers:** | |
| **Additional Information:** | |

Powered by Editorial Manager® and ProduXion Manager® from Aries Systems Corporation

| Question | Response |
| --- | --- |
| **Financial Disclosure**<br><br>Enter a financial disclosure statement that describes the sources of funding for the work included in this submission. Review the submission guidelines for detailed requirements. View published research articles from *PLOS ONE* for specific examples.<br><br>This statement is required for submission and **will appear in the published article** if the submission is accepted. Please make sure it is accurate.<br><br>**Unfunded studies**<br>Enter: *The author(s) received no specific funding for this work.*<br><br>**Funded studies**<br>Enter a statement with the following details:<br>• Initials of the authors who received each award<br>• Grant numbers awarded to each author<br>• The full name of each funder<br>• URL of each funder website<br>• Did the sponsors or funders play any role in the study design, data collection and analysis, decision to publish, or preparation of the manuscript?<br>• **NO** - Include this sentence at the end of your statement: *The funders had no role in study design, data collection and analysis, decision to publish, or preparation of the manuscript.*<br>• **YES** - Specify the role(s) played.<br><br>* typeset | The author(s) received no specific funding for this work |
| **Competing Interests**<br><br>Use the instructions below to enter a competing interest statement for this submission. On behalf of all authors, disclose any competing interests that could be perceived to bias this work—acknowledging all financial support and any other relevant financial or non-financial competing interests. | The authors have declared that no competing interests exist |

| | |
|---|---|
| This statement is required for submission and **will appear in the published article** if the submission is accepted. Please make sure it is accurate and that any funding sources listed in your Funding Information later in the submission form are also declared in your Financial Disclosure statement.<br><br>View published research articles from *PLOS ONE* for specific examples.<br><br>**NO authors have competing interests**<br><br>Enter: *The authors have declared that no competing interests exist.*<br><br>**Authors with competing interests**<br><br>Enter competing interest details beginning with this statement:<br><br>*I have read the journal's policy and the authors of this manuscript have the following competing interests: [insert competing interests here]*<br><br>* typeset | |
| **Ethics Statement**<br><br>Enter an ethics statement for this submission. This statement is required if the study involved:<br><br>• Human participants<br>• Human specimens or tissue<br>• Vertebrate animals or cephalopods<br>• Vertebrate embryos or tissues<br>• Field research<br><br>Write "N/A" if the submission does not require an ethics statement.<br><br>General guidance is provided below. Consult the submission guidelines for detailed instructions. **Make sure that all information entered here is included in the Methods section of the manuscript.** | The study was approved by the National Bioethics Committee of Chad (N° 201/PR/MESRI/DG/CNBT/2020, November 8, 2020) and the UCAD Research Ethics Committee (CER /UCAD/AD/MSN/050/2020, December 07, 2021). Prior to the study, informed consent was obtained from all participants in accordance with the approval of the Chad National Bioethics Committee and the UCAD Research Ethics Committee |

Powered by Editorial Manager® and ProduXion Manager® from Aries Systems Corporation

| | |
|---|---|
| **Format for specific study types**<br><br>**Human Subject Research (involving human participants and/or tissue)**<br>• Give the name of the institutional review board or ethics committee that approved the study<br>• Include the approval number and/or a statement indicating approval of this research<br>• Indicate the form of consent obtained (written/oral) or the reason that consent was not obtained (e.g. the data were analyzed anonymously)<br><br>**Animal Research (involving vertebrate animals, embryos or tissues)**<br>• Provide the name of the Institutional Animal Care and Use Committee (IACUC) or other relevant ethics board that reviewed the study protocol, and indicate whether they approved this research or granted a formal waiver of ethical approval<br>• Include an approval number if one was obtained<br>• If the study involved *non-human primates*, add *additional details* about animal welfare and steps taken to ameliorate suffering<br>• If anesthesia, euthanasia, or any kind of animal sacrifice is part of the study, include briefly which substances and/or methods were applied<br><br>**Field Research**<br><br>Include the following details if this study involves the collection of plant, animal, or other materials from a natural setting:<br>• Field permit number<br>• Name of the institution or relevant body that granted permission | |
| **Data Availability**<br><br>Authors are required to make all data underlying the findings described fully available, without restriction, and from the time of publication. PLOS allows rare exceptions to address legal and ethical concerns. See the PLOS Data Policy and FAQ for detailed information. | Yes - all data are fully available without restriction |

*Powered by Editorial Manager® and ProduXion Manager® from Aries Systems Corporation*

| | |
|---|---|
| A Data Availability Statement describing where the data can be found is required at submission. Your answers to this question constitute the Data Availability Statement and **will be published in the article**, if accepted.<br><br>**Important:** Stating 'data available on request from the author' is not sufficient. If your data are only available upon request, select 'No' for the first question and explain your exceptional situation in the text box.<br><br>Do the authors confirm that all data underlying the findings described in their manuscript are fully available without restriction? | |
| **Describe where the data may be found in full sentences. If you are copying our sample text, replace any instances of XXX with the appropriate details.**<br><br>• If the data are **held or will be held in a public repository**, include URLs, accession numbers or DOIs. If this information will only be available after acceptance, indicate this by ticking the box below. For example: *All XXX files are available from the XXX database (accession number(s) XXX, XXX.).*<br>• If the data are all contained **within the manuscript and/or Supporting Information files**, enter the following: *All relevant data are within the manuscript and its Supporting Information files.*<br>• If neither of these applies but you are able to provide **details of access elsewhere**, with or without limitations, please do so. For example:<br><br>*Data cannot be shared publicly because of [XXX]. Data are available from the XXX Institutional Data Access / Ethics Committee (contact via XXX) for researchers who meet the criteria for access to confidential data.*<br><br>*The data underlying the results presented in the study are available* | All relevant data are within the manuscript and its Supporting Information files. |

| | |
|---|---|
| *from (include the name of the third party and contact information or URL).*<br>• This text is appropriate if the data are owned by a third party and authors do not have permission to share the data.<br><br>* typeset | |
| Additional data availability information: | Tick here if the URLs/accession numbers/DOIs will be available only after acceptance of the manuscript for publication so that we can ensure their inclusion before publication. |

*Powered by Editorial Manager® and ProduXion Manager® from Aries Systems Corporation*

To Prof. Emily Chenette

Editor-in-Chief

PLOS ONE

August 09, 2022

Submission of a research paper for publication in PLOS ONE Journal

Dear Emily,

We would like to submit our research article entitled "Prevalence of hepatitis B virus infection and associated risk factors among students in N'Djamena, Chad" for publication in the journal PLOS ONE.

Hepatitis B is endemic in Chad and in many sub-Saharan African countries. It is transmitted vertically from mother to child at birth or by horizontal transmission especially during the first 5 years of life, putting these populations at increased risk of contracting hepatitis B.

A recent study in Chad in 2021 on patients with acute febrile jaundice found an HBsAg prevalence of 10.3%. Another study conducted in 2017 in the southwestern region of Chad reported an HBsAg prevalence of 22.9% in the general population. Although students are generally young, apparently healthy, and sexually active. They are at risk of hepatitis B infection because they had not received the hepatitis B vaccine which has been introduced into the National Immunization Program only in 2008.

There is limited data on the prevalence and risk factors associated with hepatitis B among students in Chad. Therefore, determining the prevalence of HBsAg and identifying risk factors associated with hepatitis B infection will inform the Chadian Ministry of Health and its partners in tailoring interventions for students towards the ultimate goal of eliminating hepatitis B in Chad.

We believe that this article will be of interest to your readers and contribute to the scientific debate, like the one by Tesfa.T et al (2021) published on hepatitis B virus infection and associated risk factors among medical students in eastern Ethiopia, 2021.

We confirm that this work is original, it has not been published elsewhere and is not under review for publication elsewhere. We have no conflicts of interest to disclose. All authors have agreed to submit our article to the journal PLOS ONE.

We hope you will consider this article for publication.

Yours sincerely

Nalda Debsikreo (on behalf of all authors)

Manuscript
Click here to access/download;Manuscript;Hepatitis B virus and associated risk factors among.docx

# Title: Prevalence of hepatitis B virus infection and associated risk factors among students in N'Djamena, Chad.

## Short title: Hepatitis B virus and associated risk factors among Chadian studens

**Authors:** Nalda Debsikréo[12]*, Birwé Léon Mankréo[1], Azoukalné Moukénet[13], Merwa Ouangkake[4], Nathan Mara[4], Ali Mahamat Moussa[35], Ndèye Coumba Toure-Kane[12], Françoise Lunel-Fabiani[6]

## Affiliations :

[1]Cheikh Anta Diop University, Dakar, Senegal; [2]Institut de Recherche en Santé, de Surveillance Épidémiologique et de Formation, Dakar, Senegal; [3]University of N'Djamena, N'Djamena, Chad; [4]Hôpital de la Paix, N'Djamena, Chad; [5]Centre Hospitalier Universitaire la Référence, N'Djamena, Chad; [6] Centre Hospitalier Universitaire Angers, BAT IBS - 4 rue Larrey- 49000 ANGERS, Laboratoire HIFIH, UFR Santé département Médecine, SFR 4208-UPRES EA3859, Université d'Angers, 49045 Angers Cedex 01, France

**Corresponding author:** * ndaldaeliane@gmail.com (ND)

# Abstract

## Background:

Viral hepatitis B (HBV) is a major public health threat. In Chad, HBV screening is not systematic, due to the low interest of providers or frequent shortages of supplies. Thus, most people at risk such as students are unaware of their status. However, many persons chronically infected with HBV have no or low liver disease. This study aims to determine the prevalence of HBV and identify risk factors among students in N'Djamena, Chad.

## Methods:

A cross-sectional study was conducted in July 2021 including 457 students randomly selected within the universities of N'Djamena and Emi Koussi. A structured questionnaire was used to collect data on sociodemographic characteristics and other risk factors. HBsAg testing was performed using the Determine HBsAg® Rapid Test Kit. Descriptive analyzes and binary logistic regressions were used to examine the relationship between dependent variables and sociodemographic including other risk factors. Odds ratios (ORs) and their 95% confidence intervals (CIs) were used as indicators of the association. A p value of 0.05 was used to indicate the statistical significance of the relationships.

## Results:

The HBsAg seroprevalence was 14.87%. Students aged between 26-35 years (0R= 0.90, 95% CI: 0.42-1.80; p= 0.032), who were born from HBsAg positive mothers (OR= 0.48, 95% CI: 0.08-3.84; p = 0.027), who lived with a roommate (OR= 5.42, 95% CI: 1.55-25.6; p= 0.025) or with family (OR= 2.69, 95%CI: 0.90-11.7; p= 0.025) or had a history of hepatitis (OR= 0.46, 95% CI : 0.25-0.86, p=0.015) were less likely to contract HBV infection.

## Conclusion:

The prevalence of hepatitis B is high among Chadian students. There is an urgent need to provide screening tests, HBV vaccination to improve the living conditions for students in Chad. This study highlights some solutions to improve the situation of hepatitis B in Chad and suggests policies to eliminate viral hepatitis.

**KEY WORDS:** hepatitis B seroprevalence, student, Chad

# Introduction

Viral hepatitis is a major public health threat and one of the leading causes of death worldwide. Around 900.000 deaths per year are due to hepatitis B virus infection [1]. The global seroprevalence of hepatitis B surface antigen (HBsAg) has been estimated as high as 3.6%, with the highest endemicity areas located in the African and Western Pacific regions respectively 8.8% and 5.3% [2].

In Africa where hepatitis B is a silent epidemic, more than 90 million infected people live in the sub-Saharan region, which represent 26% of the total number of people affected in the world [3] . Most people chronically infected with HBV have no active liver disease and are defined as inactive carriers or as chronic infection, according to the European Association for the Study of the Liver (EASL) classification [4]. However, subjects with persistent infection may develop cirrhosis, liver failure and/or hepatocellular carcinoma [5]. More than 124,000 Africans die each year from the consequences of undetected and untreated hepatitis [3].

In Chad, data on HBV prevalence are only available among specific population subgroups and regions due to lack of systematic research or frequent shortages of supplies [6]. A study conducted in Chad reported a prevalence of HBsAg of 22.9% in the southwestern Chad [7] and 13.5% in N'Djamena [8]. A recent study of patients with acute jaundice in Chad found an HBsAg prevalence of 10.3% [9]. HBV infection is therefore an important public health problem that requires active surveillance in Chad. However, there are few data on the epidemiology of HBV in the general population.

In highly endemic countries, hepatitis B is commonly transmitted vertically from mother to child at birth or horizontally during the first five years of life [10]. Infants infected during the perinatal period have 3.74 times (OR=3.74, 95% CI: 0.97–14.39) risk of becoming chronic carriers [11]. Parenteral transmission occurs through blood transfusion, blood exposure accidents among health professionals and use of non-sterile equipment for tattooing, piercing, acupuncture, and intravenous drug injections. These risk factors are the main causes and most frequent routes of transmission [12]. Moreover, due to the lack of systematic screening among patients attending in health care facilities [6] , most people,

including people at risk such as students, are unaware of their HBV infection serological status and do not benefit of appropriate management and treatment. Indeed, students are generally young, in good apparent health and sexually active, which puts them at risk of HBV infection. In addition, they spend more time in a crowded environment (amphitheater, dwellings, etc.) and HBV transmission may occur via saliva and skin exudates [13] .

Since the 2000s, the HBV vaccine has been introduced into the expanded immunization program (EPI) in several countries in sub-Saharan Africa to prevent HBV transmission during childhood. In Chad, the HBV vaccine was introduced into the National EPI in 2008 [14]. However, despite this policy, HBV infection prevalence continues to grow, particularly in the population over 15 years of age (the incidence in subjects over 15 years old is 26.46% versus a national incidence of 13.62%) [6]**.** This observation may inhibit the achievement of the objectives of the National Health Plan which is to eliminate the main neglected tropical diseases such as viral hepatitis, water-borne diseases and other communicable diseases by 2030 [14] . Moreover, few studies have been performed on the burden of liver disease in at-risk populations such as students in Chad. Thus, the aim of the study was to determine the prevalence of HBV and to identify the risk factors associated with HBV infection among students of the Universities of Ndjamena and Emi Koussi. Identifying these risk factors for HBsAg carriage with appropriate management would reduce the disease burden. Knowledge of the HBV infection risk factors would also guide policies aimed at reducing the HBV burden.

# Materials and Methods

## Study design and framework

A cross-sectional study was conducted in the universities of Ndjamena and Emi Koussi from July 3 to 23, 2021. The study setting included public and private institutions as well as universities. The universities of N'Djamena and Emi Koussi were selected because they have the largest number of students and N'Djamena is the city with the highest incidence of HBV (42.16%)[6] in Chad. The

University of N'Djamena is also the largest in the country with 16,142 students spread over 04 campuses and 7 faculties [15] .

## Sampling

The sample size was calculated using the single population following formula $n = (Z\alpha/2)^2 P(1-P) / i^2$ [16]. Given that there has been no similar study in a student environment in Chad, a prevalence of 50% (close to 42.12%, the incidence of HBV in N'djamena), a confidence interval of 95% ($Z\alpha/2 = 1.96$), an error margin of 5% and a provision for non-responses of 10% were used. Thus, the sample size obtained was 422 students.

The sample was distributed proportionally to faculties selected purposely based on the number of students with Faculty of Languages, Letters, Arts and Communication (75 students), Faculty of Educational Sciences (80 students), Faculty of Exact Sciences and Applied (50 students), Faculty of Economics and Management (88 students), Faculty of Human and Social Sciences (75 students) and University of Emi Koussi (50 students).

Within each faculty, a random sampling technique was applied to select study participants until the required number was reached. A first student was selected for each faculty then gradually other volunteers were enrolled until the required number was reached.

All volunteer students from the universities of N'Djamena and Emi Koussi were included in the study until the required sample was reached. Students who were not from these universities and who were unable to give informed consent were excluded from the study.

## Data collection method

A structured questionnaire was used to collect information on socio-demographic characteristics (age, sex, marital status, place of origin, faculty and level of study) and risk behaviors such as: the number of sexual partners, blood transfusion, surgery, hospitalization, HBsAg status of the mother, HBsAg status of family members, transcutaneous exposure such as acupuncture, drug use, prisoners,

unprotected sex, alcohol, sharp object wounds, mode of residence, density of residence, cloths sharing, sleeping arrangements sharing.

## Blood sample collection

A 5ml venous blood sample was collected by a lab technician in an EDTA tube after obtaining informed consent from the study participants. The blood samples were labeled with unique identification numbers and coagulated. Serum was separated by centrifugation at 3000 rpm for 5 min and placed into Eppendorf tubes.

## Selection of variables

We assessed HBV seroprevalence as blood sample tested positive for the HBV test and considered as HBsAg positive. Regarding the risk factors, potential variables retained were the student life history including number of sexual partners, blood transfusion, surgery, hospitalization, HBsAg status of mother, HBsAg status of family members, transcutaneous exposure, acupuncture, drug use, prisoners, unprotected sex, alcohol, sharp objects wounds, living arrangements, living density, shared clothing, shared sleeping arrangements, history of hepatitis B. In addition to these variables, sociodemographic characteristics of participants including their medical/not medical history were included as risk factors.

## Biological analysis

For the detection of HBsAg, the ALERE Determine® HBsAg test was used. Its principle is based on a rapid sandwich-type reaction for the detection of HBsAg. The plastic protection of each test was removed. Then, 50 µl of serum was dispensed onto the sample deposit area using a pipette. After 15 min, results were interpreted based on the appearance of color bands. The red bars appear in the control window (labeled “Control”) and the patient window (labeled “Patient”) on the strip. Any red color visible in the patient window was interpreted as a positive result (a bar). A red bar appears in the control window (annotated “Control”), the red bar in the patient window (annotated “Patient”) not appearing on the strip was interpreted as a negative result. If the red bar did not appear in the strip

control window and even if a red bar appeared in the strip patient window, the result was considered as invalid, and the test was repeated.

## Data analysis

Data were entered into an Excel database and analyzed using R version 4.1.2 (2021-11-01) software. A descriptive analysis was performed on participants' social and demographic characteristics and others risk factors stratified by HBV serological status. A Chi–square test and a Student t-test were performed to assess any significant difference in socio demographics characteristics between HBV students' status. As multivariate analysis, a logistic regression analysis was conducted to identify the factors associated with HBV serological status among Chadian students. Odds ratios (ORs) were calculated to check statistical associations between the dependent and independent variable. All variables in the study were initially tested for association with HBV status using a binary logistic regression model. Variables with $P < 0.3$ in the univariate analysis were included in the multivariate logistic regression analysis. Those showing a significant statistical association were added to the multivariable analysis model to assess whether the association existed after controlling against all the rest of the variables. A $P < 0.05$ was considered statistically significant. A 95% confidence interval were calculated for all odds ratios.

# Ethics approval and ~~consent to participate~~

The study was approved by the National Bioethics Committee of Chad (N° 201/PR/MESRI/DG/CNBT/2020, November 8, 2020) and the UCAD Research Ethics Committee (CER /UCAD/AD/MSN/050/2020, December 07, 2021). Prior to the study, informed consent was obtained from all participants in accordance with the approval of the Chad National Bioethics Committee and the UCAD Research Ethics Committee. The objectives of the survey were presented to the deans of the faculties concerned. Each participant was informed of their serological results and advised on the possible consequences of HBV infection and, if necessary, measures to be taken.

# Inclusivity in global research

Additional information regarding the ethical, cultural, and scientific considerations specific to inclusivity in global research is included in the Supporting Information (S2 Checklist).

# Results

## Sample characteristics

A total of 457 students were enrolled in this study. The socio-demographic characteristics of the participants are presented in Table 1. Seventy-six percent of participants were male, 75% were under 25 years old and 76% were single. There was no association between these sociodemographic characteristics and the HBV serology status (Table 1).

**Table 1: Socio-demographic characteristics of study participants according to their serological status, frequency (%)**

| *Variables* | | *Overall, N = 457*[1] | *HBV -, N = 389*[1] | *HBV +, N = 68*[1] | *p-value*[2] |
|---|---|---|---|---|---|
| **Age** | | 24.0 (22.0, 25.0) | 24.0 (22.0, 26.0) | 23.5 (22.0, 25.0) | 0.2 |
| | <25 | 343 (75%) | 287 (74%) | 56 (82%) | |
| | 26-35 | 94 (21%) | 82 (21%) | 12 (18%) | |
| | >35 | 20 (4.4%) | 20 (5.1%) | 0 (0%) | |
| **Sex** | | | | | >0.9 |
| | Female | 109 (24%) | 93 (24%) | 16 (24%) | |
| | Male | 348 (76%) | 296 (76%) | 52 (76%) | |
| **Marital Status** | | | | | >0.9 |
| | Single | 348 (76%) | 297 (77%) | 51 (76%) | |
| | Married | 107 (24%) | 91 (23%) | 16 (24%) | |
| | Unknown | 2 | 1 | 1 | |
| **Level of study** | | | | | 0.2 |
| | Bachelor 1 | 141 (31%) | 113 (29%) | 28 (41%) | |
| | Bachelor 2 | 143 (31%) | 123 (32%) | 20 (29%) | |
| | Bachelor 3 | 168 (37%) | 148 (38%) | 20 (29%) | |
| | Master & PhD | 5 (1.1%) | 5 (1.3%) | 0 (0%) | |

[1]Median (IQR); n (%), [2]Wilcoxon rank sum test; Fisher's exact test; Pearson's Chi-squared test

Note: FLLAC (Faculty of Languages, Letters, Arts and Communication), FSE (Faculty of Educational Sciences), FSEA (Faculty of Exact and Applied Sciences), FSEG (Faculty of Economics and Management), FSHS (Faculty of Humanities and Social Sciences), UEK (University of Emi-Koussi).

## Prevalence and risk factors associated with HBsAg ~~carriage~~

Of all the students participating in the study, 14.87% were tested positive for HBsAg (Table 2). Regarding the history of the students participating in the study, among students who had HBsAg-positive mothers, 29% were affected by HBV infection, as were students who lived in roommates (28%) and students with a known history of hepatitis B testing (23%).

Overall, few students who participated in the study had risk factors such as blood transfusion (16%), hospitalization (14%), family members carrying HBsAg+ (21%), transcutaneous exposure (14%). Moreover, the prevalence of HBV does not vary significantly with these risk factors (Table 2).

**Table 2: life history of study participants and their serological status, frequency (%)**

| Variables | Category | HBV - N (%) | HBV + N (%) | Overall, N= 457[1] | p-value[2] |
|---|---|---|---|---|---|
| **Numbers of sexual partners** | | | | | 0.7 |
| | Many | 213 (85%) | 39 (15%) | 252 (100%) | |
| | One | 176 (86%) | 29 (14%) | 205 (100%) | |
| **Knowledge of hepatitis B** | | | | | 0.9 |
| | No | 179 (86%) | 30 (14%) | 209 (100%) | |
| | Yes | 180 (86%) | 29 (14%) | 209 (100%) | |
| **Transfusion** | | | | | 0.8 |
| | No | 341 (85%) | 58 (15%) | 399 (100%) | |
| | Yes | 37 (84%) | 7 (16%) | 44 (100%) | |
| **Surgery** | | | | | 0.056 |
| | No | 367 (84%) | 68 (16%) | 435 (100%) | |
| | Yes | 21 (100%) | 0 (0%) | 21 (100%) | |
| **Hospitalization** | | | | | 0.8 |
| | No | 297 (85%) | 52 (15%) | 349 (100%) | |
| | Yes | 88 (86%) | 14 (14%) | 102 (100%) | |
| **HBsAg+Mother** | | | | | 0.037 |
| | Unknown | 73 (78%) | 20 (22%) | 93 (100%) | |
| | No | 310 (87%) | 45 (13%) | 355 (100%) | |
| | Yes | 5 (71%) | 2 (29%) | 7 (100%) | |
| **HBsAg+Family member** | | | | | 0.3 |
| | Unknown | 89 (85%) | 16 (15%) | 105 (100%) | |
| | No | 241 (87%) | 37 (13%) | 278 (100%) | |
| | Yes | 56 (79%) | 15 (21%) | 71 (100%) | |
| **Transcutaneous exposure** | | | | | 0.7 |
| | No | 342 (87%) | 50 (13%) | 392 (100%) | |
| | Yes | 12 (86%) | 2 (14%) | 14 (100%) | |

| | | | | | |
|---|---|---|---|---|---|
| **Dope** | | | | | 0.6 |
| | No | 375 (85%) | 67 (15%) | 442 (100%) | |
| | Yes | 7 (100%) | 0 (0%) | 7 (100%) | |
| **Risky practices** | | | | | 0.8 |
| | No | 274 (85%) | 47 (15%) | 321 (100%) | |
| | Yes | 115 (85%) | 21 (15%) | 136 (100%) | |
| **Prison** | | | | | 0.6 |
| | No | 366 (85%) | 63 (15%) | 429 (100%) | |
| | Yes | 21 (81%) | 5 (19%) | 26 (100%) | |
| **Unprotected sex** | | | | | >0.9 |
| | No | 136 (85%) | 24 (15%) | 160 (100%) | |
| | Yes | 253 (85%) | 44 (15%) | 297 (100%) | |
| **Sharp object Wounds** | | | | | 0.6 |
| | No | 234 (86%) | 37 (14%) | 271 (100%) | |
| | Yes | 147 (84%) | 28 (16%) | 175 (100%) | |
| | Unknown | 5 (83%) | 1 (17%) | 6 (100%) | |
| **Housing style** | | | | | 0.004 |
| | Roommate | 38 (72%) | 15 (28%) | 53 (100%) | |
| | Family | 303 (86%) | 50 (14%) | 353 (100%) | |
| | Alone | 48 (94%) | 3 (5.9%) | 51 (100%) | |
| **Housing density** | | | | | 0.5 |
| | 1-2 persons | 178 (84%) | 34 (16%) | 212 (100%) | |
| | >2 persons | 211 (86%) | 34 (14%) | 245 (100%) | |
| **Sharing clothes** | | | | | 0.5 |
| | No | 324 (85%) | 58 (15%) | 382 (100%) | |
| | Yes | 65 (88%) | 9 (12%) | 74 (100%) | |
| **Sleeping sharing** | | | | | 0.3 |
| | No | 149 (84%) | 28 (16%) | 177 (100%) | |
| | Yes | 239 (86%) | 38 (14%) | 277 (100%) | |
| **History of hepatitis B** | | | | | 0.005 |
| | No | 307 (88%) | 43 (12%) | 350 (100%) | |
| | Yes | 82 (77%) | 25 (23%) | 107 (100%) | |

# Factors associated with HBV infection among students

~~Variable with P-value less than 0.3 in the bivariate analysis~~

Binary logistic regression highlighted the factors associated with HBV carriage, such as age, HBsAg+ mother, history of Hepatitis B tests, level, family members, dope and type of housing. (Table 3).

In multivariate logistic regression, after adjustment of the regression considering other risk factors significantly associated with HBV carriage, the regression showed that only the age, mother's serological status, the mode of residence and the history of hepatitis B remained significantly associated with HBV carriage. Students aged between 26-35 years were 1.11 times less likely to be

HBsAg positive (OR= 0.90, 95% CI: 0.42-1.80; p= 0.032). Participants with HBV-uninfected mothers were 2.08 times less likely to be HBsAg positive (OR=0.48, 95% CI: 0.10-4.26; p= 0.027). Students who lived with roommates or family had respectively 5.41 times (OR=5.41, 95% CI: 1.55- 25.6; p= 0.025) and 2.69 times (OR=2.69, 95% CI: 0.90-11.7; p= 0.025) risk to be HBsAg carriers than those who lived. Students with no history of Hepatitis B test were 2.17 times less likely to be HBsAg positive (OR= 0.46, 95% CI: 0.25-0.86, p=0.015) (Table 3).

**Table 3: Factors associated with HIV status of study participants**

| | | Univariate regression | | | Multivariate regression | | |
|---|---|---|---|---|---|---|---|
| **Variables** | **categories** | **OR[1]** | **95% CI[1]** | **p-value** | **OR[1]** | **95% CI[1]** | **p-value** |
| **Age** | | | | 0.025 | | | 0.032 |
| | < 25 | — | — | | — | — | |
| | 26-35 | 0.75 | 0.37-1.42 | | 0.90 | 0.42-1.80 | |
| | >35 | 00 | | | 00 | | |
| **Level** | | | | 0.14 | | | 0.16 |
| | Bachelor 1 | | — | — | | — | — |
| | Bachelor 2 | 0.66 | 0.35-1.22 | | 0.54 | 0.27-1.06 | |
| | Bachelor 3 | 0.55 | 0.29-1.01 | | 0.52 | 0.26-1.02 | |
| | Master & PhD | 0.00 | | | 0.00 | | |
| **Mother HBsAg+** | | | | 0.075 | | | 0.027 |
| | Yes | — | — | | — | — | |
| | Unknown | 0.68 | 0.14- 5.03 | | 1.61 | 0.23-14.6 | |
| | No | 0.36 | 0.08-2.59 | | 0.48 | 0.08-3.84 | |
| **HBsAg+Family member** | | | | 0.28 | 0.28 | | 0,23 |
| | Yes | | — | — | | — | — |
| | Unknown | 0.67 | 0.31-1.47 | | 0.42 | 0.14-1.16 | |
| | No | 0.57 | 0.30-1.14 | | 0.84 | 0.40, 1.82 | |
| **Dope** | | | | | 0.13 | | |
| | Yes | | — | — | | — | — |
| | No | 0.00, NA | 2,796-376 | | 0.00, NA | 9,892-022 | |
| **Housing style** | | | | 0.005 | | | 0.025 |
| | Alone | — | — | | — | — | |
| | Roommate | 6.32 | 1.91-28.7 | | 5.41 | 1.55-25.6 | |
| | Family | 2.64 | 0.92-11.1 | | 2.69 | 0.90-11.7 | |
| **History of hepatitis B examination** | | | | 0.007 | | | 0.015 |
| | Yes | — | — | | — | — | |
| | No | 0.46 | 0.27-0.80 | | 0.46 | 0.25-0.86 | |

Note: Note: 95% CI = 95% Confidence Interval, NA = Not applicable (not retained in the model), OR = Odd Ratio.

## Discussion

To inform policies aiming to reduce the burden of HBV in Chad and to achieve the objectives of the national health plan 2016-2030, this study was designed to determine the prevalence of HBV and to identify the HBV infection risk factors, among students at the Universities of N'djamena and Emi Koussi. The HBsAg seroprevalence was 14.87%, among the included students. As already shown, the HBsAg+ serological status of the mother, the mode of residence and the history of hepatitis B were associated with the carriage of HBV among the students of the universities of N'djamena and Emi Koussi. On the other hand, the presence of family members carrying HBV and the number of sexual partners were not significantly associated with HBV carriage.

Regarding the seroprevalence of HBsAg, these results are similar to those of the study conducted in N'djamena which showed a prevalence of 13.5% ( [8] and are higher than in the study on patients with acute febrile jaundice in Chad, where the prevalence reached 10.3% [9]. These differences might be related to socio-cultural, demographic, economic and behavioral factors. Most of the students were young, of different origins and cultures, with many sexual partners and various sexual practices. These are factors that may contribute to increase the rate of infection. Other studies conducted elsewhere have reported lower or higher prevalence than in our study. The seroprevalence of HBsAg found in this study is higher than that reported in studies conducted in Togo (4.6%) [17], Cameroon (5.6%) [18] and Ethiopia (11.5%)[19], while it is lower than that of Nigeria (31.5%) (20]) . While our results are consistent with those reported in Central African Republic (15.5%) (21]. The high seroprevalence in this study implies an improvement in awareness, particularly among young people and students. It implies as well as a generalization of HBV vaccination and particularly catch-up vaccination of children at school, before, and screening HBsAg and antiHBc and HBs antibodies of university or high school students before the beginning of the studies.

On the other hand, 23.4% of all students had already been screened for hepatitis B and the seroprevalence of HBsAg was 23% among these students. However, the seroprevalence of HBsAg was 12% among students who had not been previously tested. This result corroborates the WHO study

which highlighted that more than 124,000 Africans die each year from the consequences of undetected and untreated hepatitis [3]. This finding could be explained by the low effectiveness of awareness-raising activities. Many people in Africa are screened following a medical consultation. However, in Chad, this screening is not systematic and not required by clinicians [6]. Therefore, asymptomatic patients remain a potential source of HBV infection.

Our results are similar to other studies that have reported no association between HBV infection and risk factors such as history of surgery, acupuncture and blood transfusion, smoking and alcohol consumption [18,21,22] . These results could be explained by the low proportion of participants with this background risks in the current study. Students aged between 25-35 years of age were less likely to contract HBV. A study conducted among Senegalese military personnel showed that participants aged 27-37 years were less likely to contract HBV (OR=0,16 95% CI =0,04-0,68; p=0,012)[23]. This result could be explained by clearance of HBsAg leading to spontaneous healing of hepatitis B or occult hepatitis.

Students with HBsAg-negative mothers were statistically unlikely to acquire HBV infection. These results support findings from the literature reporting that sub-Saharan Africa is a highly endemic area where perinatal infection is common[24]. The same findings were observed in Gambia in 2007, where mother-to-child transmission was responsible for 16% of chronic infections [25] and in Cameroon, where children born from HBsAg positive mothers had a substantial risk of mother-to-child transmission [26]. These results highlight the importance of a policy aimed at preventing mother-to-child transmission of HBV.

Our study also found that students who lived in shared accommodation are more likely to contract HBV infection. This suggests a decent home in addition to a protective factor against HBV infection. Therefore, our findings suggest that authorities could help students to improve student's life conditions.

The study also found that participants who knew their HBV serological status were twice as likely to be HBV carriers. Indeed, this result could illustrate a limitation of the study so far as participation in the

study was voluntary. Thus, students who know they are already infected could be tempted to participate in the study hoping to benefit from the study in terms of clinical management and care or hoping to verify their serological status.

## Strengths and limitations of the study

This is a multicenter study bringing together a diverse population of students from the universities of N'djamena and Emi-Koussi. This reflects the epidemiology of HBV within this community. The study did not include an assay for anti-HBc and HBeAg IgM markers. This could have made it possible to distinguish between acutely infected and chronic carriers. Quantification of HBV DNA would also have allowed the identification of cases of occult hepatitis.

## Conclusions

HBV is one of the neglected tropical diseases of major concern in Chad and health authorities plan to eliminate it by 2030. This study conducted in the universities setting revealed a prevalence of 14.87%. HBsAg+ mother and roommate status were risk factors associated with HBsAg carriage. These results suggest the need for improved awareness, screening and increasing vaccination coverage among young people and particularly among women. This study can also serve as a basis for other subsequent medical and scientific studies on neglected tropical diseases in young people.

## List of abbreviations

Anti-HBs: Hepatitis B surface antibody; AOR: Adjusted odd ratio; CI: Confidence interval; COR: Crude odd ratio; EPI: Expanded Program of Immunization; HBsAg: Hepatitis B surface antigen; HBV: Hepatitis B virus; HCC: Hepatocellular carcinoma; WHO: World Health Organization.

## Acknowledgements

We are thankful to laboratory technicians of Hôpital de la Paix in N'djamena who have performed the blood sampling and tests. We express our gratitude to data collectors and supervisors of the survey. We thank the deans of Cheikh Anta Diop University for their guidance. We are thankful to the

secretaries of the students who have facilitated the sensitization of students to participate to the study and the students who participated in the study.

### Availability of data and materials

All relevant data are within the paper and supporting information files.

## Conflicts of interest

The authors have declared that no competing interests exist.

## Funding

The author(s) received no specific funding for this work.

## Author Contributions

Conceptualization: Nalda Debsikréo, Françoise Lunel-Fabiani.

Data curation: Nalda Debsikréo, Birwé Léon Mankréo, Azoukalné Moukénet.

Formal analysis: Nalda Debsikréo, Birwé Léon Mankréo, Azoukalné Moukénet.

Funding acquisition: Nalda Debsikréo.

Investigation: Nalda Debsikréo, Merwa Ouangkake, Nathan Mara, Ali Mahamat Moussa.

Methodology: Nalda Debsikréo, Birwé Léon Mankréo, Azoukalné Moukénet,Ali Mahamat Moussa Ndèye Coumba Toure-Kane, Françoise Lunel-Fabiani.

Project administration: Nalda Debsikréo, Merwa Ouangkake, Nathan Mara, Ali Mahamat Moussa.

Software: Nalda Debsikréo, Birwé Léon Mankréo, Azoukalné Moukénet.

Laboratory: Nalda Debsikréo, Merwa Ouangkake, Nathan Mara.

Validation: Nalda Debsikréo ;Birwé Léon Mankréo, Azoukalné Moukénet, Merwa Ouangkake, Nathan Mara, Ali Mahamat Moussa, Ndèye Coumba Toure-Kane, Françoise Lunel-Fabiani.

Visualization: Nalda Debsikréo, Birwé Léon Mankré, Azoukalné Moukénet.

Writing – original draft: Nalda Debsikréo

Writing – review & editing: Nalda Debsikréo, Birwé Léon Mankré, Azoukalné Moukénet, Merwa Ouangkake, Nathan Mara, Ali Mahamat Moussa, Ndèye Coumba Toure-Kane, Françoise Lunel-Fabiani.

# References


1. WHO. World Hepatitis Day 2020 [Internet]. [cité 22 juin 2022]. Disponible sur: https://www.who.int/campaigns/world-hepatitis-day/2020
2. Schweitzer A, Horn J, Mikolajczyk RT, Krause G, Ott JJ. Estimations of worldwide prevalence of chronic hepatitis B virus infection: a systematic review of data published between 1965 and 2013. Lancet Lond Engl. 17 oct 2015; 386(10003):1546‑55.
3. WHO. World Hepatitis Day, 28 July 2021 [Internet]. WHO | Regional Office for Africa. [cité 22 juin 2022]. Disponible sur: https://www.afro.who.int/regional-director/speeches-messages/world-hepatitis-day-28-july-2021
4. Koffas A, Kumar M, Gill US, Jindal A, Kennedy PTF, Sarin SK. Chronic hepatitis B: the demise of the 'inactive carrier' phase. Hepatol Int. 1 avr 2021; 15(2):290‑300.
5. Ferreira MS. Diagnosis and treatment of hepatitis B. Rev Soc Bras Med Trop. août 2000; 33:389‑400.
6. Tcd_rpt_annuairesstatistiquessanitaires_ministeresante_2017.pdf [Internet]. [cité 13 mai 2022]. Disponible sur: https://www.humanitarianresponse.info/sites/www.humanitarianresponse.info/files/documents/files/tcd_rpt_annuairesstatistiquessanitaires_ministeresante_2017.pdf
7. Suesstrunk J, Djongali FB. Hepatitis B virus prevalence in rural areas in south-west Chad. Trop Doct. oct 2017;47(4):374‑7.
8. Bessimbaye N, Moussa AM, Mbanga D, Tidjani A, Mahamat SO, Ngawara MN, et al. Séroprévalence de l'Ag HBs et de l'anticorps Anti VHC chez les personnes infectées par le VIH1 à N'Djamena, Tchad. Bull Société Pathol Exot. 1 déc 2014; 107(5):327‑31.
9. Yandai FH, Traore KA, Moussa AM, Ouoba BL, Ouoba JB, Bolti MA, et al. Prevalence and Diversity of Hepatitis Virus Markers among Patients with Acute Febrile Jaundice in Chad. Microbiol Res. déc 2021; 12(4):878‑87.
10. Zampino R, Boemio A, Sagnelli C, Alessio L, Adinolfi LE, Sagnelli E, et al. Hepatitis B virus burden in developing countries. World J Gastroenterol. 14 nov 2015; 21(42):11941‑53.
11. Tharmaphornpilas P, Rasdjarmrearnsook A orn, Plianpanich S, Sa-nguanmoo P, Poovorawan Y. Increased risk of developing chronic HBV infection in infants born to chronically HBV infected mothers as a result of delayed second dose of hepatitis B vaccination. Vaccine. 19 oct 2009; 27(44):6110‑5.
12. Mast EE, Alter MJ, Margolis HS. Strategies to prevent and control hepatitis B and C virus infections: a global perspective. Vaccine. 26 mars 1999; 17(13‑14):1730‑3.
13. Gomes C, Wong RJ, Gish RG. Global Perspective on Hepatitis B Virus Infections in the Era of Effective Vaccines. Clin Liver Dis. août 2019; 23(3):383‑99.

14. Tchad-Politique-Nationale-de-Sante-2016-2030.pdf [Internet]. [cité 13 mai 2022]. Disponible sur: https://www.prb.org/wp-content/uploads/2020/06/Tchad-Politique-Nationale-de-Sante-2016-2030.pdf

15. La formation à l'université de N'Djaména.Disponible sur: https://archive.wikiwix.com/cache/index2.php?url=https%3A%2F%2Fwww.universite-ndjamena.td%2Fformation#federation=archive.wikiwix.com

16. Charan J, Biswas T. How to Calculate Sample Size for Different Study Designs in Medical Research? Indian J Psychol Med. 2013; 35(2):121‑6.

17. Ekouevi DK, Thomas A, Sewu D, Lawson-Ananissoh L, Tchounga B, Salou M, et al. Prevalence of Hepatitis B among Students from the University of Lomé, Togo in 2015. Open J Epidemiol. 8 août 2017; 7(3):262‑72.

18. servais albert fiacre E. Statut Vaccinal Contre le Virus de l'Hépatite Virale B et portage de l'Antigène Hbs chez les Etudiants en Médecine et en Pharmacie de l'Université de Douala au Cameroun. Health Sci Dis. 1 janv 2014;

19. Tesfa T, Hawulte B, Tolera A, Abate D. Hepatitis B virus infection and associated risk factors among medical students in eastern Ethiopia. PLOS ONE. 19 févr 2021; 16(2):e0247267.

20. Tula MY, Iyoha O. A Cross-sectional Study on the Sero-prevalence of Hepatitis B Surface Antigen (HBsAg) among Apparently Healthy Students of a Tertiary Institution in North-Eastern Nigeria. Int J Trop Dis Health. 10 janv 2015; 7:102‑8.

21. Komas NP, Baï-Sepou S, Manirakiza A, Léal J, Béré A, Le Faou A. The prevalence of hepatitis B virus markers in a cohort of students in Bangui, Central African Republic. BMC Infect Dis. 29 juill 2010; 10:226.

22. Mutocheluh M, Owusu M, Kwofie TB, Akadigo T, Appau E, Narkwa PW. Risk factors associated with hepatitis B exposure and the reliability of five rapid kits commonly used for screening blood donors in Ghana. BMC Res Notes. 4 déc 2014; 7(1):873.

23. Diop M, Diouf A, Seck SM, Lo G, Ka D, Massaly A, et al. [Prevalence of hepatitis B surface antigen and its associated factors in Senegalese military personnel sent on mission to Darfur]. Pan Afr Med J. 2017; 26:154.

24. Shimakawa Y, Toure-Kane C, Mendy M, Thursz M, Lemoine M. Mother-to-child transmission of hepatitis B in sub-Saharan Africa. Lancet Infect Dis. 1 janv 2016; 16(1):19‑20.

25. Shimakawa Y, Lemoine M, Njai HF, Bottomley C, Ndow G, Goldin RD, et al. Natural history of chronic HBV infection in West Africa: a longitudinal population-based study from The Gambia. Gut. 1 déc 2016; 65(12):2007‑16.

26. Shimakawa Y, Veillon P, Birguel J, Pivert A, Sauvage V, Guillou-Guillemette HL, et al. Residual risk of mother-to-child transmission of hepatitis B virus infection despite timely birth-dose vaccination in Cameroon (ANRS 12303): a single-centre, longitudinal observational study. Lancet Glob Health. 1 avr 2022; 10(4):e521‑9.

# Supporting information

S1 File. Questionnaire (docx)

S2 File. Checklist (docx)

S3 File. Data_base R (XLS)

S4 File code R

S3 File. Data_base

Click here to access/download
**Supporting Information**
BDE.xlsx

S4 File code R

Click here to access/download
**Supporting Information**
ANALYSE-FIANALE.docx

S1 File. Questionnaire

Click here to access/download
**Supporting Information**
S1 Questionnaire.docx

S2 File. Checklist

Click here to access/download
**Supporting Information**
S2 Check list.docx
