# Supplementary material for: Prevalence of hepatitis B virus infection and its associated factors among students in N’Djamena, Chad
Source: PLoS One. 2024 Apr 18;19(4):e0273589. doi: 10.1371/journal.pone.0273589 (PMC11025733; doi:10.1371/journal.pone.0273589)
Supplement: Attachment — Submitted filename: PONE-D-22-22361_R1_Annotated_July2023.pdf [file pone.0273589.s009.pdf]

PLOS ONE

Prévalence et facteurs associés de l'infection par le virus de l'hépatite B chez les étudiants à N'Djamena, Tchad

--Manuscript Draft--

**Manuscript Number:**	PONE-D-22-22361R1
**Article Type:**	Research Article
**Full Title:**	Prévalence et facteurs associés de l'infection par le virus de l'hépatite B chez les étudiants à N'Djamena, Tchad
**Short Title:**	Hepatitis B virus and associated factors among Chadian studens
**Corresponding Author:**	NALDA DEBSIKREO<br>Cheikh Anta Diop University of Dakar: Universite Cheikh Anta Diop de Dakar<br>Dakar, SENEGAL
**Keywords:**	KEY WORDS: hepatitis B, HBsAg, prevalence, associated factors, student, Chad.
**Abstract:**	Abstract<br><br>Background<br>Hepatitis B virus (HBV) is a major public health problem. In Chad, HBV screening is not systematic, due to the low interest among health care providers or frequent shortages of medical supplies. Thus, most people at risk such as students are unaware of their status. Many people with chronic HBV infection are asymptomatic. This study aimed to determine the prevalence of HBV and identify factors among students in N'Djamena, Chad.<br>Methods<br>A cross-sectional survey of students at the Universities of N'Djamena and Emi Koussi was conducted from 3 to 23 July 2021. Students were included in the study using a consecutive sampling. Blood samples were collected, and serum was tested for hepatitis B surface antigen (HBsAg) using the Determine HBsAg® rapid test kit and confirmed by Abbott Architect i1000SR analyzer based on chemiluminescent microparticle immunoassay (CMIA). Descriptive analysis, binary and multivariate logistic regressions were used to examine the relationship between dependent variables and socio-demographic factors including other associated factors.<br>Results A total of 457 students aged between 19 to 59 years were included in the study. The prevalence of HBV infection was 14.87% (95% CI =13,9–21,7%). Most students, 91%, were in the 20–30-year age group, 64.9% reported unprotected sex, 53.6% reported multiple sexual partners and 45.7% of students had no knowledge of hepatitis B. Having a HBsAg+ mother (AOR: 2.60; 95%CI: 1.18-5.80, p=0.018), a transcutaneous procedure history (AOR: 2.63 95%CI: 1.32-5.10, p=0.005), being in a roommate (AOR: 7.19, 95%CI 2.08-33.79, p=0.004) and having history of HBV screening (AOR: 2.03, 95%CI: 1.11-3.65, p=0.019) were associated with a higher risk of HBV infection. Conclusion<br>The 14.87% prevalence rates found in our study confirm that Chad is a highly endemic country. Having a HBsAg+ mother, living in a shared flat, having a history of transcutaneous procedure and a history of HBV screening were factors independently associated with HBV infection among students. Policies, such as routine screening of the population, birth dose vaccine and access to treatment are needed to reduce the incidence and mortality of HBV related diseases.
**Order of Authors:**	NALDA DEBSIKREO
	Birwé Léon Mankréo
	Azoukalné Moukénet
	Merwa Ouangkake
	Nathan Mara
	Ali Mahamat Moussa
	Ndèye Coumba Toure-Kane

Powered by Editorial Manager® and ProduXion Manager® from Aries Systems Corporation

	Françoise Lunel-Fabiani
**Opposed Reviewers:**	
**Response to Reviewers:**	RESPONSE TO REVIEWERS<br>Reviewer #1: The study covers a significant problem especially in Africa and other third world countries.<br>Comments<br>Grammatical typo errors should not be overlooked Page 5 Sampling<br>We thank reviewer 1 for comments. We answered to all comments and correct typo (p 5) or grammatical errors.<br>Line 104-105: how are the students selected, are they self-selected? Randomly selected, systematic random sampling? It is not clear.<br>Thanks for the comment, based on number of students in faculties of the two largest universities of N'Djamena, students who volunteered to participate in the study were enrolled according to consecutive sampling. Line: 121-123.<br>Data collection method: how did you prepare the structured tool? Was it standard? Did you refere some other works (cite).<br>A structured questionnaire was used to collect data. Line: 135.<br>Who collected the data? How many data collectors, their profession…<br>Laboratory technicians collected the data under the supervision of the hepatitis focal point in Chad. There were five collectors.<br>Biological analysis what is the sensitivity and specificity of the kit?<br>The kit had a sensitivity of 99.7% and a specificity of (99.5%) Reference: Bottero and t al J Hepatol Performance of rapid tests for detection of HBsAg and anti-HBsAb in a large cohort, France 2013<br><br>Table 2: the table is long (more than one page), can break it down:<br>Thank you, we changed this table according to the other reviewers comments. Line: 255-256.<br><br>Last row: history of HBV? It is not clear how the test turns negative from previous positive test? Are they cured?<br>Thanks for the comment: we were meaning history of hepatitis, or jaundice, we have clarified. 347-348.<br><br>Line 198-201: it is not clear how the interpretation is made Participants 197 with HBV-uninfected mothers were 198 2.08 times less likely to be HBsAg positive (OR=0.48, 95% CI: 0.10-4.26; p= 0.027). Students who lived with roommates or family had respectively 5.41 times (OR=5.41, 95% CI: 1.55- 25.6; p= 0.025) and 2.69 times (OR=2.69, 95% CI: 0.90-11.7; p= 0.025) risk to be HBsAg carriers than those who lived alone. Students who had a no history of Hepatitis B screening test were 2.17 times more????? likely to be HBsAg positive (OR= 0.46, 95% CI0.25-0.86, p=0.015) (Table 3)<br><br>Thank you, we have clarified the results section in the new version. Line: 36-44<br>Table 3: please revise the interpretation by considering the confidence interval what do you mean by OR1 two times? How did you deal with variables which do not fulfill the chi square criteria?<br>The independent t-test was used to compare the means of continuous variables. When normal distribution or equality of variance could not be assumed, the Mann-Whitney U test was used. The comparison of proportions was assessed by means of the Chi-square test. Line: 175<br>line 224: how does high prevalence imply improvement in awareness?<br>Thank you, we have changed the sentence "and removed "this result corroborates….<br>Discussion: please revise following the correct result interpretation<br>Thank you, we have revised the interpretation and comments. Line: 268-368.<br>Reviewer #2:<br>Dear Author, I am wishing to acknowledge author's work entitled: 'Prevalence of hepatitis B virus infection and associated risk factors among students in N'Djamena, Chad'. The results illustrated the prevalence of hepatitis B virus among students. Therefore, this study will be useful for future prevention and control of hepatitis infection through the knowledge of prevalence HBV and risk factors among students. The manuscript is more or less in line with the vision, objectives and instructions of PLoS One. Despite its potential, however, this manuscript contains major errors in data

presentation and interpretation, grammatical errors which need to be corrected (see the attached file).
General Comments
1. Although what authors are reporting is not a novelty, they must however be commended for their efforts in reporting what is prevalence of HBV among students in their locality. But there are so many researches worldwide which were done by more advanced lab techniques like ELISA and molecular techniques (PCR).
We thank reviewer for comments, we carried out additional tests by ARCHITECT HBsAg Quantitative confirming the results. Line: 162-168
2. Use of English language is poor in certain sections and would require a detailed revision.
Thank you for raising the point about grammatical errors, we carried out copyediting of the manuscript to improve the quality of its English.

3. Based on PLoS One authors' guideline, figures must be uploaded separately in tif or other form.
Thanks for reminding us about the respect of style requirement of PLOS One related to figures. However, we have not a figure to integrate into this paper.

Title
• The title was good. But why you removed the area name 'Emi Koussi' from the title? You talked more about this study area in the methods part.
Thank you, "Emi Koussi" is the name of one of the universities whereas the study area itself is N'djamena.

Abstract
• The punctuation colon (:) in each subheading was not necessary.
Thank you for the remark. We removed it accordingly.
• Line 17; read as 'Viral hepatitis B (HBV) is a major public health threat'. It is better written as 'Viral hepatitis B virus (HBV) is a major public health problem'. Here the word 'threat' is not the appropriate word.
Thank you for mentioning this. We modified accordingly in -- line 20; "Hepatitis B virus (HBV) is a major public health problem. Thank you, we changed the sentence according to your suggestion.
• Line 20-21; 'This study aims to determine the prevalence-------------' It is better written as, 'This study aimed to determine the prevalence--------------------'
Thank you, we changed the sentence according to your suggestion Line: 24-25.
• Line 23; '-------- conducted in July 2021 including 457 students randomly selected-------------'. Here a specific length of time should be mentioned. Similarly, which random sampling technique was used? Simple or systematic?
Thank you for your comment; we reviewed the methodology section. Line 27-28 " A cross-sectional survey of students at the Universities of N'Djamena and Emi Koussi was conducted from 3 to 23 July 2021. Students were included in the study using a consecutive sampling". Line 28-29. Simple random sampling technique was used to calculate sample size. Participants were selected consecutively but based on the volunteering to participate into the study.

• Which specimen type you have processed? It should be described before you talk about the method.
•Thanks, the type of sample taken was serum. Line 29.
• Line 26; 'Descriptive analyzes and binary logistic regressions were used to examine the relationship between dependent variables and sociodemographic including other risk factors'. It is better you say, 'Descriptive analysis and binary logistic regressions were used to examine the relationship between dependent variables and socio-demographic factors including other risk factors.'
Thank you for mentioning this. We replaced the sentence as suggested: line170-171; 'Descriptive analysis and binary logistic regressions were used to examine the relationship between dependent variables and socio-demographic factors including other risk factors.
• Among how many study participants the study conducted?
Thank you, Line 127-134 "A minimum sample size of 422 students was calculated. Nevertheless, we have interviewed and collected sample from 457 students." The University of N'Djamena has 16,142 students. line: 115-116
• In the result part, it is better you include both the numerator and denominator together

	with percentage in presenting prevalence, for example A/B (14.87%).<br>Thank you for this comment, however we are afraid the tables will be too clumpy, although we put, in the narrative, after percentage the numerator and denominator between bracket to help the reader to understand the underlining calculation of percentages and proportions.<br>• Too short and shallow result presentation<br>Your result completely contrasts the fact. Are you interpreting the result inversely? See the data, 'Students aged between 26-35 years (0.032), who were born from HBsAg positive mothers (0.027), who lived with a roommate (0.025) or had a history of hepatitis (p=0.015) were morelikely to contract HBV infection'. For example, A participant who lived with a roommate has a probability of acquiring HBV 4.5 times than those living lonely (OR= 5.42, 95% CI: 1.55-25.6; p= 0.025). The author better go through the analysis and correct mistakes that appeared.<br>"Students aged between 26-35 years (0.032), who were born from HBsAg positive mothers (0.027), who lived with a roommate (0.025) or had a history of hepatitis (p=0.015) were more likely to contract HBV infection'<br>Thank you, we reviewed the analysis accordingly and we changed the result section and add more details and modified the interpretation which was wrong. Students were more likely…line:238-243.<br><br>• What is your baseline to say 'high prevalence of HBV infection'?<br>Thank you for this remark. By the way, we would rather say that this prevalence, higher than 8%, places our study population in the high endemicity group according to the WHO classification. We changed the sentence in the manuscript Line: 272-273.<br><br>• Which type of conclusion is it? 'This study highlights some solutions to improve the situation of hepatitis B in Chad and suggests policies to eliminate viral hepatitis'. : This study highlights that HBV prevalence is still high in young adults i.e students in Chad.Thus, It is time to improve national health policies in Chad like hepatitis B prevention measures such as HBs Ag screening in pregnant women, vaccination at birth, catch up vaccination in children and students; and also to provide tests for screening the population, and, finally improve the management and treatment of infected subjects.<br>Thank you, you are right this study does highlight that HBV prevalence is still high in young adults i.e. students in Chad. Thus, we changed the sentence to line: 45-51.<br><br>• Additional key words:-HBsAg, risk factor<br><br>Thank you we added the suggested key words. Line: 52<br>• It should be re-written and make adjustments based on the comments in the methods and results part that follows.<br>Thank you, we took this remark into account. Lin: 26-34.<br>Introduction<br>• The introduction seems like literature review, so it is better modified.<br>Thank you, we changed the introduction section, according to your suggestion Line 53-107.<br>• The word threat is not the appropriate word; please replace by other word.<br>Thank you, we changed the sentence according to your suggestion. Line 54.<br>• The introduction described the burden among clinically ill participants, not apparently healthy individuals (e.g. students). Therefore, the author better show the burden of HBV in similar or related study populations.<br>Thank you, we changed the introduction according to your suggestion, by changing some references. Line 91-94.<br>• There was no description about the microbiology of HBV.<br>Thank you, we added a paragraph about the microbiology of HBV according to your suggestion. LINE 55-58.<br>• I do not think the importance of writing values for P-value, 95%CI and OR. For example, 'Infants infected during the perinatal period 62 have 3.74 times (OR=3.74, 95% CI: 0.97–14.39) risk of becoming chronic carriers [11]'.<br>Thank you, we rewrote this sentence.<br>• Line 82-84 should be deleted. 'Identifying these risk factors for HBsAg 83 carriage with appropriate management would reduce the disease burden. Knowledge of the HBV 84 infection risk factors would also guide policies aimed at reducing the HBV burden'.

Powered by Editorial Manager® and ProduXion Manager® from Aries Systems Corporation

	Thank you, we deleted the sentence.<br><br>Materials and Methods<br>• Line 93; the subheading should be ‘Sample size determination and sampling technique’<br>Thank you, we considered the title ‘Sample size determination and sampling technique’: line119.<br><br>• About the sampling technique, please see the comment in the abstract<br>Thanks, Students were included in the study using a consecutive sampling”. Line 28-29. Simple random sampling technique was used to calculate sample size. Participants were selected consecutively but based on the volunteering to participate into the study.<br>• The ‘selection of variables’ subheading better be replaced by ‘Socio-demographic data collection’<br><br>Thank you, we considered the title ‘Socio-demographic data collection’: line 143.<br><br>• The ‘Biological analysis’ also should be replaced by ‘Laboratory data processing’<br>Thank you, we considered the title ‘Laboratory data processing’: line 153.<br><br>• In the ethical clearance part, were all participants above the age of 18 years old? If not, why did not you took assent from participants below the age of 18 years old?<br><br>Thank you, all participants were above the age of 18 years old. Age ranged from 19 to 59 years. Line 209.<br>• Your participants were apparently healthy individuals (students). There should be a mechanism in place to link those who need treatment with the healthcare system. HBsAg positive students were managed by the hepatitis B focal point line: 199-201. How do you manage the psychological and potential social harm on HBV positives that do not need treatment? So ethical issues were not well described.<br>Thank you for raising this point, HBV-positive people were referred to the focal point of the program “fight against hepatitis in Chad” for follow-up and advice. line 199-201.<br>• Specimen collection and processing needs to be well written.<br>After HBs Ag screening test, sera samples were frozen and kept at …°C in the…laboratory.<br>After HBs Ag screening test, serum samples were frozen and kept at -80°C in the biobank laboratory of the national reference hospital Line: 166-167.<br>Results<br>• Your subheading ‘Sample characteristics’ was not the appropriate one. It is better you replace by, ‘Socio-demographic characteristics.<br>•Thank you, we considered the title ‘Socio-demographic characteristics’: line 207.<br>• Based on your sample size determination your sample size was 422. Why you included 457 in the result part?<br>Thank for raising this point. We provided details to ease the understanding of the readers Line : 127-134 “A minimum sample size of 422 students was calculated. Nevertheless, we interviewed and collected samples from 457 students”.<br>• You mix up the socio-demographic characteristics of participants with prevalence of HBV as well as risk factors. I recommend you present, first the socio-demographic characteristics and then prevalence of HBV, finally risk factors for HBV.<br>Thank you, we took this comment into account. the socio-demographic characteristics line: 207, prevalence of HBV line: 234 and Factors associated with HBV infection among students line: 244.<br>• What is your base to classify the age group? Have you associated with possible risks of interest? It is better again re-group and perform the analysis.<br>Thank you, we reclassified the Age category: <20, 20 – 30, 30- 40 and > 40, referring to the article “bdoul Rahamane Njigou Mawouma, Amadou Hapsatou Djoulatou, Eliane Ornella Komnang, Etienne Omolomo Kimessoukie. Facteurs associés à l´infection de l´hépatite B chez les femmes enceintes dans les formations sanitaires du district de santé de Mokolo/Région de l´Extrême-Nord Cameroun. PAMJ. 21 Jan 2022. 41(61)”<br><br>• Is there any difference between the two subheadings? ‘Factors associated with HBV infection among students’ and ‘Prevalence and risk factors associated with HBsAg carriage’. For me it was not clear.

	Thank you, we changed the titles. Line: 1<br><br>• It was difficult to understand/catch up the concepts of the tables. They lacked OR and 95%CI. Yet you presented in the text. From where you get it (OR and 95%CI)?<br><br>Thank you, we improved the reading of the tables and bring out the odds ratios and their 95% confidence intervals. Line 263-264.<br>• Similarly, it is better you represent the crude odds ratio by COR and the adjusted odds ratio by AOR than simply by OR.<br><br>Thank you, we took into consideration your suggestion, we represented the crude odds ratio by COR and the adjusted odds ratio by AOR. Line263-264.<br><br>• All table titles should be complete in description, area, and period of study.<br><br>Thank you for this advice, we took them into account: Table 1: Socio-demographic characteristics of study participants (line219-220), Table 2: Estimated prevalence of HBsAg (line 255-256) and Table 3: Factors associated with HBV status of study participants (line263-264).<br><br>Discussions<br>• Since the necessity of doing this work was already described in the introduction part. So here writing about it was not necessary. Therefore, delete the following. Line 206 'To inform policies aiming to reduce the burden of HBV in Chad and to achieve the objectives of the 207 national health plan 2016-2030, this study was designed to determine the prevalence of HBV and to 208 identify the HBV infection risk factors, among students at the Universities of N'djamena and Emi Koussi'.<br><br>Thank you, we deleted these sentences.<br><br>• Authors tried to compare their findings with different reports from worldwide. They also better justify the reason for variation among results of different research findings with respect to theirs' based on actual situation; most of the reason for variation mentioned in the discussion part were already intensively described by other authors.<br><br>Thank you, we improved the discussion accordingly. Line: 268-357<br><br>Discussion<br>• Most of the literatures used for comparisons were researches on patients. In this case these literatures were incomparable to this study.<br><br>Thank you, we rewrote the discussion: line268-357.<br><br>Conclusions<br>• Normally, conclusion emanates from your findings of the research.<br><br>Thank you for the remark, the conclusion has been rewritten. line: 368-378.<br><br>• Revise it and accommodate recommendation of community/government importance.<br>Thank you for the remark, the recommendation has been rewritten. Line: 379-387.<br><br>Reviewer #3:<br>In the submitted manuscript "Prevalence of hepatitis B virus infection and associated risk factors among students in N'Djamena, Chad" (Manuscript Number: PONE-D-22-22361), the authors wrote an original research article describing the prevalence of HBsAg positivity and factors associated for being HBsAg-positivity among university students in N'Djamena, Chad.<br><br>HBV infection is still a major health problem worldwide, especially in Africa including Chad. In Chad, HBV screening is not systematic. Most of infected people are unaware of their status. This study aims to determine the prevalence of HBsAg positivity and to identify factors associated with being HBsAg-positivity. The authors conducted a cross-sectional study among 457 university students in N'Djamena, Chad in July 2021.

HBsAg seroprevalence determined by the Determine HBsAg rapid test was 15%. After logistic regression analyses, several factors associated with being HBsAg-positivity were identified. The study results indicate that there is an urgent need to test HBV and provide vaccination in student population in Chad.
However, in my point-of-view, there are some major and minor points that need to be improved or clarified:
1. The “Title” and “Abstract” section,
a. Please consider to use the word “Associated factors” rather than the word “Risk factors” for this cross-sectional study (REF: Antay-Bedregal D, Camargo-Revello E, Alvarado GF. Associated factors vs risk factors in cross-sectional studies. Patient Prefer Adherence. 2015 Nov 13;9:1635-6. doi: 10.2147/PPA.S98023), and also for the entire article.

Thank you for highlighting this comment, we have considered "associated factors".

b. Texts in the results part of the abstract is confused.
• “Students aged between 26-35 years (0R=0.90, 95% CI: 0.42-1.80; p= 0.032), who were born from HBsAg positive mothers (OR=0.48, 95% CI: 0.08-3.84; p = 0.027), who lived with a roommate (OR= 5.42, 95% CI:1.55-25.6; p= 0.025) or with family (OR= 2.69, 95%CI: 0.90-11.7; p= 0.025) or had a history of hepatitis (OR= 0.46, 95% CI : 0.25-0.86, p=0.015) were less likely to contract HBV infection.” In general, if OR > 1 means “they were more likely to contract HBV infection, as compared to the reference”. In contrast, if OR < 1 means “they were less likely to contract HBV infection, as compared to the reference”
Thank you, the result section has been changed. Line: 36-44.
2. In the “Introduction” section,
a. In line 56, the authors should specify the population of each study, for example, Ref# 7 was conducted in general population, Ref# 8 was conducted in HIV population.
Thank you, we specified the study population. Line 77-79.
b. Line 61-62, authors wrote “Infants infected during the perinatal period have 3.74 times (OR=3.74, 95% CI: 0.97–14.39) risk of becoming chronic carriers [11]”. This sentence may mislead the meaning of the origin article. Ref#11 indicated that “Children having intervals longer than 10 weeks between their first two HB vaccine doses were at greater risk of developing chronic HBV infection. Their risk was 3.74 times higher than that of those with intervals shorter than 10 weeks (95% CI = 0.97–14.39).
Thanks, these sentences have been deleted.
c. Line 62-65, “Parenteral transmission occurs through blood transfusion, blood exposure accidents among health professionals and use of non-sterile equipment for tattooing, piercing, acupuncture, and intravenous drug injections. These risk factors are the main causes and most frequent routes of transmission [12].” Currently, this sentence may not be all correct. Please also update the reference.
Thanks, we updated the reference.
d. Line 68-71, “Indeed, students are generally young, in good apparent health and sexually active, which puts them at risk of HBV infection. In addition, they spend more time in a crowded environment (amphitheater, dwellings, etc.) and HBV transmission may occur via saliva and skin exudates [13]”. Ref #13 is not relevant to the sentence.
Thanks, the reference "13" was deleted.
e. Line 79-80, “Moreover, few studies have been performed on the burden of liver disease in at-risk populations such as students in Chad.” Please add the references. Or the authors would like to mention that “Study on the burden of HBV infection among student population in Chad is still limited.”, Please clarify.
We wanted in fact to mention that “There is no studies on the burden of HBV infection among students’ population in Chad.” Line: 103-104.
3. In “Material and Methods” section,
a. Line 95-96, “a prevalence of 50% (close to 42.12%, the incidence of HBV in N'djamena)” Please add the reference.
Thanks, this sentence was deleted.
b. In part “Biological analysis” (line 131-140), it is already a well-known information, the paragraph should be removed and be referred to the manufacturer’s recommendation.
Thanks for the remark, the paragraph has been deleted and be referred to the manufacturer’s recommendation. Line: 161.
4. In “Results” section, I would like to suggest the authors to re-analyze the data again with an advice from statisticians.
a.In Table 1, superscripted number#1 does not make sense, as well as the Table’s

Powered by Editorial Manager® and ProduXion Manager® from Aries Systems Corporation

footnote (line 176-178)
Thanks, superscripted number#1 and the Table's footnote were deleted.
b.Indeed, Table 1 should demonstrate "Baseline sociodemographic, socioeconomic and other characteristics of the study population". No HBsAg results needed.
Thank you, we took this comment into account in the new version. Line: 219-220.
c.Table 2 should demonstrate "HBsAg seroprevalence among the study population, probably describe in detail according to the baseline characteristics."
Thank you, we took this comment into account; Line: 255-256.

d. Table 3 should demonstrate "Univariate and multivariable analysis of factors associated for HBsAg positivity among the study population". The authors did not indicate the reference group (OR=1) in the table. Table 3 Indeed, table 2 can probably be merged with table 3.
Thank you, we cannot merge table 2 and table 3 because table 2 talks about prevalence and table 3 about logistic regression.
C.Data with missing value should not be taken to bivariate analysis, univariate and multivariable logistic regression analyses.
Thank you for the suggestion.
D. Indeed, a 95% confidence interval for the odds ratio also provides a test of the null hypothesis that the odds ratio is 1 at the 5% significance level. If the confidence interval does not include 1, we reject H0 and conclude that the odds for the two groups are different; if the interval does include 1, the data do not provide enough evidence to distinguish the groups in this way. The authors could not conclude that "Students aged between 26-35 years were 1.11 times less likely to be HBsAg positive (0R= 0.90, 95% CI: 0.42-1.80; p= 0.032)" (Line 196). Also, it is happened with other variables such as students with HBV-uninfected mothers and who lived with HBsAg+ family.
Thanks, we performed the analysis again. Line: 249-254.
g. There are many good samples available for this kind of analysis, for example:
• Bancha B, Kinfe AA, Chanko KP, Workie SB, Tadese T (2020) Prevalence of hepatitis B viruses and associated factors among pregnant women attending antenatal clinics in public hospitals of Wolaita Zone, South Ethiopia. PLoS ONE 15(5): e0232653.
Thank you, we followed this model.
• Argaw, B., Mihret, A., Aseffa, A. et al. Sero-prevalence of hepatitis B virus markers and associated factors among children in Hawassa City, southern Ethiopia. BMC Infect Dis 20, 528 (2020).
• Kassaw B, Abera N, Legesse T, Workineh A, Ambaw G. Sero-prevalence and associated factors of hepatitis B virus among pregnant women in Hawassa city public hospitals, Southern Ethiopia: Cross-sectional study design. SAGE Open Med. 2022 Dec 6;10:20503121221140778.
• Makuza, J.D., Rwema, J.O.T., Ntihabose, C.K. et al. Prevalence of hepatitis B surface antigen (HBsAg) positivity and its associated factors in Rwanda. BMC Infect Dis 19, 381 (2019).
• Etc.
5. In "Discussion" section,
a. Need more improvement after re-analysis.
The discussion has been improved after the new analysis Line: 268-357.
b. The author compared their results with other studied which conducted in different population, likes in HIV-infected people, in hospitalized patients…
We modified this section. Line: 277-278.
c. How the authors ensure on data of HBsAg-positive family members? Questionnaires survey may provide a wrong result.
We have based our work on Questionnaires survey.
Reviewer #4:
Methods
1) Kindly describe the randomization procedure. Was it done at the faculty level? or was the random number generation for the whole population.
Thanks for this comment, A list of all faculties of the two largest universities of N'Djamena were obtained from its respective administrations. Based on number of students in faculties of the two largest universities of N'Djamena, students who volunteered to participate in the study were enrolled according to consecutive sampling. It does not concern the whole population.
Results
2) If the majority of students are less than 25, should your age categories be re-classified? If so, please consider it.

	Thank you, we reclassified the Age category: <20, 20 – 30, 30- 40 and > 40. referring to the article "bdoul Rahamane Njigou Mawouma, Amadou Hapsatou Djoulatou, Eliane Ornella Komnang, Etienne Omolomo Kimessoukie. Facteurs associés à l´infection de l´hépatite B chez les femmes enceintes dans les formations sanitaires du district de santé de Mokolo/Région de l´Extrême-Nord Cameroun. PAMJ. 21 Jan 2022. 41(61)"<br>3) the only sexual history is single/many partners. Do you have any additional data?<br>Thank you, we also have the data on unprotected sex : line 226-227.<br>4) The first table overall, HbsAg+ and HbsAg-; and second table HbsAg+, HbsAg-, and overall. Please be consistent in presentation of tables.<br>Thank you, we have redone the table1. Line: 219-220.<br>5) How did you build univariate/multivariate models? You have only p value for each group. How come? Kindly explain.<br>Thank you, please see the methods section.181-187.
**Additional Information:**	
**Question**	**Response**
**Financial Disclosure**<br><br>Enter a financial disclosure statement that describes the sources of funding for the work included in this submission. Review the submission guidelines for detailed requirements. View published research articles from *PLOS ONE* for specific examples.<br><br>This statement is required for submission and **will appear in the published article** if the submission is accepted. Please make sure it is accurate.<br><br>**Unfunded studies**<br>Enter: *The author(s) received no specific funding for this work.*<br><br>**Funded studies**<br>Enter a statement with the following details:<br>• Initials of the authors who received each award<br>• Grant numbers awarded to each author<br>• The full name of each funder<br>• URL of each funder website<br>• Did the sponsors or funders play any role in the study design, data collection and analysis, decision to publish, or preparation of the manuscript?<br>• **NO** - Include this sentence at the end of your statement: *The funders had no role in study design, data collection and analysis, decision to publish, or preparation of the manuscript.*<br>• **YES** - Specify the role(s) played.<br><br>* typeset	The author(s) received no specific funding for this work

Powered by Editorial Manager® and ProduXion Manager® from Aries Systems Corporation

**Competing Interests**<br><br>Use the instructions below to enter a competing interest statement for this submission. On behalf of all authors, disclose any competing interests that could be perceived to bias this work—acknowledging all financial support and any other relevant financial or non-financial competing interests.<br><br>This statement is required for submission and **will appear in the published article** if the submission is accepted. Please make sure it is accurate and that any funding sources listed in your Funding Information later in the submission form are also declared in your Financial Disclosure statement.<br><br>View published research articles from *PLOS ONE* for specific examples.<br><br>**NO authors have competing interests**<br><br>Enter: *The authors have declared that no competing interests exist.*<br><br>**Authors with competing interests**<br><br>Enter competing interest details beginning with this statement:<br><br>*I have read the journal's policy and the authors of this manuscript have the following competing interests: [insert competing interests here]*<br><br>* typeset	The authors have declared that no competing interests exist
**Ethics Statement**<br><br>Enter an ethics statement for this submission. This statement is required if the study involved:<br><br>• Human participants<br>• Human specimens or tissue<br>• Vertebrate animals or cephalopods	The study was approved by the National Bioethics Committee of Chad (N° 201/PR/MESRI/DG/CNBT/2020, November 8, 2020) and the UCAD Research Ethics Committee (CER /UCAD/AD/MSN/050/2020, December 07, 2021). Prior to the study, informed consent was obtained from all participants in accordance with the approval of the Chad National Bioethics Committee and the UCAD Research Ethics Committee

• Vertebrate embryos or tissues<br>• Field research<br><br>Write "N/A" if the submission does not require an ethics statement.<br><br>General guidance is provided below. Consult the submission guidelines for detailed instructions. **Make sure that all information entered here is included in the Methods section of the manuscript.**	

Powered by Editorial Manager® and ProduXion Manager® from Aries Systems Corporation

**Format for specific study types**<br><br>**Human Subject Research (involving human participants and/or tissue)**<br>• Give the name of the institutional review board or ethics committee that approved the study<br>• Include the approval number and/or a statement indicating approval of this research<br>• Indicate the form of consent obtained (written/oral) or the reason that consent was not obtained (e.g. the data were analyzed anonymously)<br><br>**Animal Research (involving vertebrate animals, embryos or tissues)**<br>• Provide the name of the Institutional Animal Care and Use Committee (IACUC) or other relevant ethics board that reviewed the study protocol, and indicate whether they approved this research or granted a formal waiver of ethical approval<br>• Include an approval number if one was obtained<br>• If the study involved *non-human primates*, add *additional details* about animal welfare and steps taken to ameliorate suffering<br>• If anesthesia, euthanasia, or any kind of animal sacrifice is part of the study, include briefly which substances and/or methods were applied<br><br>**Field Research**<br><br>Include the following details if this study involves the collection of plant, animal, or other materials from a natural setting:<br>• Field permit number<br>• Name of the institution or relevant body that granted permission	
**Data Availability**<br><br>Authors are required to make all data underlying the findings described fully available, without restriction, and from the time of publication. PLOS allows rare exceptions to address legal and ethical concerns. See the PLOS Data Policy and FAQ for detailed information.	Yes - all data are fully available without restriction

A Data Availability Statement describing where the data can be found is required at submission. Your answers to this question constitute the Data Availability Statement and **will be published in the article**, if accepted.<br><br>**Important:** Stating 'data available on request from the author' is not sufficient. If your data are only available upon request, select 'No' for the first question and explain your exceptional situation in the text box.<br><br>Do the authors confirm that all data underlying the findings described in their manuscript are fully available without restriction?	
**Describe where the data may be found in full sentences. If you are copying our sample text, replace any instances of XXX with the appropriate details.**<br><br>• If the data are **held or will be held in a public repository**, include URLs, accession numbers or DOIs. If this information will only be available after acceptance, indicate this by ticking the box below. For example: *All XXX files are available from the XXX database (accession number(s) XXX, XXX.).*<br>• If the data are all contained **within the manuscript and/or Supporting Information files**, enter the following: *All relevant data are within the manuscript and its Supporting Information files.*<br>• If neither of these applies but you are able to provide **details of access elsewhere**, with or without limitations, please do so. For example:<br><br>*Data cannot be shared publicly because of [XXX]. Data are available from the XXX Institutional Data Access / Ethics Committee (contact via XXX) for researchers who meet the criteria for access to confidential data.*<br><br>*The data underlying the results presented in the study are available from (include the name of the third party*	All relevant data are within the manuscript and its Supporting Information files.

and contact information or URL).<br>• This text is appropriate if the data are owned by a third party and authors do not have permission to share the data.<br><br>* typeset	
Additional data availability information:	Tick here if the URLs/accession numbers/DOIs will be available only after acceptance of the manuscript for publication so that we can ensure their inclusion before publication.

Powered by Editorial Manager® and ProduXion Manager® from Aries Systems Corporation

To Prof. Emily Chenette
Editor-in-Chief
PLOS ONE
Submission of a revised version of a research paper for publication in PLOS ONE Journal

Dear Emily,
Please, find enclosed the revised version of our research article entitled "Prevalence of hepatitis B virus infection and associated factors among students in N'Djamena, Chad" for publication in the journal PLOS ONE, modified according to Editor and reviewers comments.
We have corrected typos and grammatical errors in the whole paper. We have also provided details about the sampling design, and a context explanation of the high prevalence of hepatitis B in Chadian students.
We have modified the conclusion according to the Editor and reviewers comments and added explanations about the variable “history of hepatitis in the family member(s) and housing style.
We hope this new version is now suitable for publication in your journal.

Yours sincerely
Nalda Debsikreo (on behalf of all authors)
Cover Letter

Manuscrit

Click here to access/download;Manuscript;Manuscript reviewed.doc

Title: Prevalence and associated factors of hepatitis B virus infection among students in N'Djamena, Chad.

Short title: Hepatitis B virus and associated factors among Chadian students

Authors: Nalda Debsikréo[12]*, Birwé Léon Mankréo[1], Azoukalné Moukénet[13], Merwa Ouangkake[4], Nathan Mara[4], Ali Mahamat Moussa[35], Ndèye Coumba Toure-Kane[12], Françoise Lunel-Fabiani[6]

Affiliations:

[1]Cheikh Anta Diop University, Dakar, Senegal.

[2]Institut de Recherche en Santé, de Surveillance Épidémiologique et de Formation, Dakar, Sénégal ;

[3]University of N'Djamena, N'Djamena, Chad.

[4]Hôpital de la Paix, N'Djamena, Chad ;

[5]Centre Hospitalier Universitaire la Référence, N'Djamena, Chad ;

[6] Centre Hospitalier Universitaire Angers, BAT IBS-4 rue Larrey-49000 ANGERS, Laboratoire HIFIH, UFR Santé département Médecine, SFR 4208-UPRES EA3859, Université d'Angers, 49045 Angers Cedex 01, France

Corresponding author: * ndaldaeliane@gmail.com (ND)

Abstract

Background

Hepatitis B virus (HBV) is a major public health problem. In Chad, HBV screening is not systematic, due to the low interest among health care providers or frequent shortages of medical supplies. Thus, most people at risk such as students are

unaware of their status. Many people with chronic HBV infection are asymptomatic. This study aimed to determine the prevalence of HBV and identify factors among students in N'Djamena, Chad.

Methods

A cross-sectional survey of students at the Universities of N'Djamena and Emi Koussi was conducted from 3 to 23 July 2021. Students were included in the study using a consecutive sampling. Blood samples were collected, and serum was tested for hepatitis B surface antigen (HBsAg) using the Determine HBsAg® rapid test kit and confirmed by Abbott Architect i1000SR analyzer based on chemiluminescent microparticle immunoassay (CMIA). Descriptive analysis, binary and multivariate logistic regressions were used to examine the relationship between dependent variables and ~~socio-demographic factors including other~~ associated factors**.**

Results

A total of 457 students aged between 19 to 59 years were included in the study. The prevalence of HBV infection was 14.87% (95% CI =13,9–21,7%). Most students, 91%, were in the 20–30-year age group, 64.9% reported unprotected sex, 53.6% reported multiple sexual partners and 45.7% of students had no knowledge of hepatitis B. Having a HBsAg+ mother (AOR: 2.60; 95%CI: 1.18-5.80, p=0.018), a transcutaneous procedure history (AOR: 2.63 95%CI: 1.32-5.10, p=0.005), being in a roommate (AOR: 7.19, 95%CI 2.08-33.79, p=0.004) and having history of HBV screening (AOR: 2.03, 95%CI: 1.11-3.65, p=0.019) were associated with a higher risk of HBV infection**.**

Conclusion

The 14.87% prevalence rates found in our study confirm that Chad is a highly endemic country. Having a HBsAg+ mother, living in a shared flat, having a history of

transcutaneous procedure and a history of HBV screening were factors independently associated with HBV infection among students. Policies, such as routine screening of the population, birth dose vaccine and access to treatment are needed to reduce the incidence and mortality of HBV related diseases.

KEY WORDS: hepatitis B, HBsAg, prevalence, associated *factors, student*, Chad.

Introduction

Hepatitis B is a major public health problem, despite the availability of a prophylactic vaccine, and one of the leading causes of death worldwide (1). Hepatitis B virus (HBV) is a DNA, partially circular virus belonging to the Hepadnaviridae family. It is a small, enveloped virus responsible for acute, sometimes fulminant hepatitis and for chronic hepatitis. It has also an oncogenic potential (2). Chronic HBV infection is widespread, and 900,000 deaths per year are related to HBV infection (3) and its prevalence is 3.6% (4).

Despite a lack of accurate data on viral hepatitis B in Africa, it is estimated that its prevalence is one of the highest in the world. Sub-Saharan Africa has a high prevalence of HBsAg exceeding 8% in general population (5), and more than 90 million people are HBV infected, representing 26 % of the total number of affected people worldwide (6). Since chronic hepatitis B infection remains silent for a long time ~~and~~ ~~is unnoticed~~, the population remains under-informed about possible prevention and about the risks and consequences of hepatitis B infection. This contributes to lower health coverage and an increased risk of HBV infection in the population (7). with the risk of developing cirrhosis or hepatocellular carcinoma (HCC) (8).

Low-income countries have a high burden of HBV infections, however testing and access to treatment remain limited in these countries. More than 124.000 Africans

die each year as a result of undetected and untreated hepatitis (6). Therefore, screening people who have not yet been diagnosed is a major challenge in eliminating hepatitis B (9).

In Chad, HBV prevalence data are available only for specific population subgroups and regions, due to the lack of systematic research (10). A study conducted in Chad reported a HBsAg prevalence of 22.9% in the general population of southwestern of the country (11) and 13.5% in the HIV population of N'Djamena (12). A recent study of patients with acute jaundice in Chad found a prevalence of HBsAg of 10.3%. (13). HBV infection is therefore a significant public health concern that requires active surveillance in Chad.

HBV can be transmitted from biological fluids of infected individuals, especially blood and sexual secretions. Transmission of HBV is vertical or horizontal in the perinatal period in areas of high endemicity (14). Parenteral transmission occurs through blood transfusion, blood exposure accident in health professionals and the use of non-sterile equipment for tattooing, piercing, acupuncture, intravenous drug injection and transmission may also occur through unprotected sexual intercourse (3) (15).

Students, who are typically the youngest, apparently healthy, and sexually active, engage in risky behaviours such as alcohol abuse, multiple sexual partners and are exposed to sexually transmitted diseases. In addition, some studies found that family history of HBV, sexual activity and socioeconomic conditions were the main risk factors for HBV infection among students in Bangui (16) and asymptomatic adolescents in Nigeria (17).

Since the 2000s, the HBV vaccine has been introduced into the Expanded Program on Immunization (EPI) of several sub-Saharan African countries to prevent

transmission of HBV during childhood. In Chad, the HBV vaccine was introduced into the national plan in 2008 (18). Despite this policy, the prevalence of HBV infection continues to increase, especially in the population over 15 years of age (incidence in subjects over 15 years of age is 26.46% versus a national incidence of 13.62%) (19). This could hamper the achievement of the National Health Plan targets to eliminate major tropical diseases such as viral hepatitis, water-borne diseases and other communicable diseases by 2030 (20). Studies on the burden of HBV infection among the student population and factors associated with hepatitis B are limited in the community, making it necessary to conduct studies to guide policies in preventing this scourge in Chad. Therefore, this study aimed to determine the prevalence of HBV and to identify associated factors among students in N'Djamena, Chad.

Materials and Methods

Study design and framework

A cross-sectional study was conducted in the universities of N'djamena and Emi Koussi from July 3 to 23, 2021. The study setting included public and private universities. The universities of N'Djamena and Emi Koussi were selected because they have the largest number of students and N'Djamena is the city with the highest incidence of HBV (42.16%) in Chad (19). The University of N'Djamena is also the largest public university in the country with 16,142 students ~~spread over~~ 4 campuses and 7 faculties (20) and The Emi Koussi university is one of largest private universities with around 3,000 students and 02 faculties spread over two campuses (22).

Sample size determination and sampling technique

A list of all faculties of the two largest universities of N'Djamena were obtained from its respective administrations. Based on number of students in faculties of the two

largest universities of N'Djamena, students who volunteered to participate in the study were enrolled according to consecutive sampling.

The sample size was calculated using the single population following formula $n= (Z\alpha/2)^2 P (1-P) / i^2$. Given that there is no similar study in a student environment in Chad, a prevalence of 50%, a Z score value of 1.96 with an alpha level of 0.05 (95% confidence) and a relative 10% precision. A minimum sample size of 422 students was calculated. Nevertheless, we interviewed and collected sample from 457 students. The sample size calculated was distributed proportionally to the number of students in faculties. (Faculty of Languages, Letters, Arts and Communication (75 students), Faculty of Educational Sciences (80 students), Faculty of Exact and Applied Sciences (50 students), Faculty of Economics and Management (88 students), Faculty of Human and Social Sciences (75 students) and University of Emi Koussi (50 students)).

Data collection method

A structured questionnaire was used to collect information on socio-demographic characteristics (age, sex, marital status, place of origin, faculty and study level) and risk behaviors such as: number of sexual partners, blood transfusion, surgery, hospitalization, HBsAg status of the mother, family history of HBV infection, HBsAg status of family members, transcutaneous exposure such as acupuncture, drug use, prison stay, unprotected sex, alcohol, sharp object wounds, mode of residence, density of residence, clothes sharing, sleeping arrangements.

Socio-demographic data collection

HBV prevalence was calculated on the number of blood samples which tested positive for HBsAg test. Regarding the risk factors, potential variables retained were the student life history including number of sexual partners, a history of blood

transfusion, surgery, hospitalization, the HBsAg status of mother, a family history of HBV infection, transcutaneous exposure, acupuncture, drug use, prison stay, unprotected sex, alcohol, sharp objects wounds, living arrangements, living density, shared clothing, shared sleeping arrangements, a history of hepatitis B. In addition to these variables, sociodemographic characteristics of participants including their medical/non-medical history were included as risk factors.

Laboratory data processing

A 5ml venous blood sample was collected by a lab technician in an EDTA tube after obtaining informed consent from the study participants. The blood samples were labeled with unique identification numbers and coagulated. Serum was separated by centrifugation at 3000 rpm for 5 min and placed into Eppendorf tubes. HBsAg was searched in sera samples using ALERE Determine® HBsAg test, which is based on the principle of immunochromatography. This is a qualitative test for the rapid detection of HBsAg, with very good sensitivity and specificity (100%) [20]. The analyses were performed in accordance with the manufacturers' instructions.

Positive results were confirmed by Abbott Architect i1000SR analyzer (Abbott Diagnostics, Abbott Park, IL, USA) based on chemiluminescent microparticle immunoassay (CMIA) for the qualitative detection of HBsAg. HBsAg testing was done an ARCHITECT HBsAg Qualitative assay according to the manufacturer's instructions. After HBs Ag screening test, sera samples were frozen and kept at -80°C in the biobank laboratory of the national reference hospital.

Data analysis

Data were entered into an Excel database and analyzed using R version 4.1.2 (2021-11-01) software. Descriptive analysis and logistic regressions were used to examine

the relationship between dependent variables and socio-demographic factors including other risk factors. Quantitative variables were expressed as the mean ± SD. Categorical variables were expressed as numbers (percentages). The independent t-test was used to compare the means of continuous variables. When normal distribution or equality of variance could not be assumed, the Mann-Whitney U test was used. The comparison of proportions was assessed by means of the Chi-square test. In both cases, the level of significance was set at p < 5%. As multivariate analysis, a logistic regression analysis was conducted to identify the factors associated with HBV serological status among students. Adjusted Odds ratios (AORs) were calculated to check statistical associations between the dependent and independent variable after controlling against all the rest of the variables. All variables that were less than 20% significant (i.e., with a p-value < 20%) in univariate analysis were included in the initial multivariate logistic regression model. The forced variables, i.e., age and sex, were also included in the multivariate analysis, even though they did not have a p-value < 20% in the univariate analysis. To obtain the final multivariate model, a logistic regression was then performed. Variables were selected according to the statistical criteria of selection by descending stepwise procedure. The final model was selected with the exclusion of the variables that were not significant, using Akaike's information criterion as an index of parsimony. Variables with a p-value < 5% were considered statistically significant. A 95% confidence interval were calculated for all odds ratios.

Ethical consideration

The study was approved by the National Bioethics Committee of Chad (N° 201/PR/MESRI/DG/CNBT/2020, November 8, 2020) and the UCAD Research Ethics Committee (CER /UCAD/AD/MSN/050/2020, December 07, 2021). written informed

consent was obtained from all participants for inclusion in the study. The objectives of the survey were presented to the deans of the faculties concerned. Each participant was informed of their serological results and advised on the possible consequences of HBV infection and, if necessary, measures to be taken. HBV-positive people were referred to the focal point of the program fight against hepatitis in Chad for follow-up and advice.

Inclusivity in global research

Additional information regarding the ethical, cultural, and scientific considerations specific to inclusivity in global research is included in the Supporting Information (S2 Checklist).

Results

Socio-demographic characteristics

Out of the total of 457 students who participated in the study, 348 were male and 109 were female with a sex ratio (M / F) 3.19. Age ranged from 19 to 59 years. The mean age of the students was 24 ±3 SD years and 91% of student were between the age group of 20 and 30 years. Most of participants to the study were from University of N'djamena (407, 89%). Around three out of four study participants were single (76%). **(Table 1).**

Table 1: Socio-demographic characteristics of study participants

Characteristic	Category	Frequency	Percent (%)
**Age**	<20 Ans	8	1.8
	20 - 30	417	91
	30- 40	17	3.7
	> 40	15	3.3
**Level of study**	Bachelor1	141	31
	Bachelor2	143	31
	Bachelor3	168	37
	Master & PhD	5	1.1
**Marital Status**	Married	109	24
	Single	348	76
**Sex**	Female	109	24
	Male	348	76
**Site**	FLLAC	75	16
	FSE	99	22
	FSEA	50	11
	FSEG	100	22
	FSHS	83	18
	UEK	50	11

Note: FLLAC (Faculty of Languages, Letters, Arts and Communication), FSE (Faculty of Educational Sciences), FSEA (Faculty of Exact and Applied Sciences), FSEG (Faculty of Economics and Management), FSHS (Faculty of Humanities and Social Sciences), UEK (University of Emi-Koussi).

Socio-cultural and behavioral exposure status among participants

Of the 457 participants, 64.9% (297/457) reported having unprotected sex, 53.6% (245/457) reported having multiple sexual partners, while 45.7% (209/457) of participants were unaware of hepatitis B and 76.5% (350/457) had no history of HBV screening.

Most of the participants - 77.2% (353/457) - lived in a family environment, 53.6% (245/457) lived in a dwelling with more than two persons per room and 60.6% (277/457) reported sharing a bed. Furthermore, 38.2% (175/457) also shared sharp objects **(Table 2).**

Prevalence of HBV

Of the total students participating in the study, 68/457 (14.87% (95% CI = 13.9-21.7%)) were positive for HBsAg. The proportion of HBsAg positive cases was significantly higher among students with an HBsAg positive mother than among those with an HBsAg negative mother (29 vs 13%). There was a significant difference (p= 0.004) between students who lived alone (5.9%) and those who lived with roommates (28 %). Students with a history of HBV screening were also significantly more often HBsAg positive (2 vs 12%) followed by those with a family history of HBV infection (21 vs 13 %), or a history of jail stay (19 vs 15 %) and those who had transfusion (16 vs 15 %) (**Table 2).**

Factors associated with HBV infection among students.

Logistic regression was used to test the association between HBV infection and the independent variables and to identify risk factors. By considering P-value <0.20 in bivariate analysis, having a HBsAg positive mother, a family history of HBV infection, a history of transcutaneous procedure, Housing style and a history of HBV screening were identified as candidate variables for a multivariate analysis. Having HBsAg positive mother (AOR: 2.60; 95%CI: 1.18-5.80, p=0.018), Having a history of transcutaneous procedure (AOR: 2.63 95%CI: 1.32-5.10, p=0.005), having a flat share (AOR: 7.19, 95%CI 2.08-33.79, p=0.004) and having a history of HBV screening (AOR: 2.03, 95%CI: 1.11-3.65, p=0.019) were significantly associated with HBV status **(Table 3).**

Table 2: Estimated prevalence of HBsAg

Variable	Category	Overall n (%)	HBsAg negative n (%)	HBsAg positive n (%)	p
**Sex**	Female	109 (23,8%)	93(85,3%)	16(14,6%)	>0.9
	Male	348 (76,1%)	296(85,05%)	52(14,9%)	
**Numbers of sexual partners**	more than one	252 (55.1%)	213 (85%)	39 (15%)	0.7
	One	205 (44.8%)	176 (86%)	29 (14%)	
**Knowledge of hepatitis B**	No	209(45.7 %)	179 (86%)	30 (14%)	0.9
	Yes	209(45.7%)	180 (86%)	29 (14%)	
**Transfusion**	No	372(81.4%)	341 (85%)	58 (15%)	0.8
	Yes	44(9.62%)	37 (84%)	7 (16%)	
**History of surgical procedure**	No	435 (95.1%)	367 (84%)	68 (16%)	0.056
	Yes	21(4.5%)	21 (100%)	0 (0%)	
**History of hospital admission**	No	349(76.3%)	297 (85%)	52 (15%)	0.8
	Yes	102(22.3%)	88 (86%)	14 (14%)	
**HBsAg + mother**	No	355(77.6%)	310 (87%)	45 (13%)	0.037
	Yes	7(2.7%)	5 (71%)	2 (29%)	
**Family history of HBV infection**	No	278 (60.8%)	241 (87%)	37 (13%)	0.3
	Yes	71 (15.5%)	56 (79%)	15 (21%)	
**History of Transcutaneous procedure**	No	392(85.7%)	342 (87%)	50 (13%)	0.7
	Yes	14(3.06%)	12 (86%)	2 (14%)	
**History of injection of drugs**	No	442(96.7%)	375 (85%)	67 (15%)	0.6
	Yes	7 (1.5%)	7 (100%)	0 (0%)	
**Risky practices**	No	321(70.2%)	274 (85%)	47 (15%)	0.8
	Yes	136 (29.7%)	115 (85%)	21 (15%)	
**Prison stays**	No	429 (98.8%)	366 (85%)	63 (15%)	0.6
	Yes	22(5.6%)	21 (81%)	5 (19%)	
**Unprotected sex**	No	160(35%)	136 (85%)	24 (15%)	>0.9
	Yes	297(64.9%)	253 (85%)	44 (15%)	
**Sharing of sharp materials**	No	271 (59.2%)	234 (86%)	37 (14%)	0.6
	Yes	175 (38.2%)	147 (84%)	28 (16%)	
**Housing style**	Alone	51 (11.1%)	48 (94%)	3 (5.9%)	0.004
	Roommate	53 (11.5%)	38 (72%)	15 (28%)	
	Family	353 (77.2%)	303 (86%)	50 (14%)	
**Housing density**	1-2 persons	212 (46.3%)	178 (84%)	34 (16%)	0.5
	>2 persons	245 (53.6%)	211 (86%)	34 (14%)	
**Sharing clothes**	No	382(83.5%)	324 (85%)	58 (15%)	0.5
	Yes	74(16.1%)	65 (88%)	9 (12%)	
**Sharing bed**	No	177(38.7%)	149 (84%)	28 (16%)	0.3
	Yes	277(60.6%)	239 (86%)	38 (14%)	
**History of HBV screening**	No	350(76.5%)	307 (88%)	43 (12%)	0.005
	Yes	107(23.4%)	82 (77%)	25 (23%)	

Table 3: Factors associated with HBV status of study participants.

Variable	Univariate regression		Multivariate regression	
	COR (95% CI)	P	AOR (95% CI)	P
**HBsAg positive mother**				
**No**	1		1	
**Yes**	2.01 (1.13-3.48)	0.015	2.60 (1.18-5.80)	0.018*
**Family history of hepatitis B**				
**No**	1		1	
**Yes**	1.74 (0.88-3.35)	0.102	1.03 (0.47-2.14)	0.936
**Transcutaneous procedure**				
**No**	1		1	
**Yes**	2.62 (1.39-4.81)	0.002	2.63 (1.32-5.10)	0.005*
**Housing style**				
**Alone**	1		1	
**Family**	2.64 (0.92-11.15)	0.114	2.94 (0.98-12.78)	0.089
**Roommate**	6.32 (1.91-28.71)	0.006	7.19 (2.08-33.79)	0.004*
**History of HBV screening**				
**No**	1		1	
**Yes**	2.18 (1.24-3.75)	0.006	2.03 (1.11-3.65)	0.019*

Note: 95% CI = 95% Confidence Interval, model), COR: crude odds ratio, 1; reference category, *; significantly associated factors p-value <0.05; AOR and adjusted odds ratio.

Discussion

We found, in a population of 457 students from the University of N'Djamena and the University of Emi Koussi, Chad, a prevalence of HBV infection (HBsAg positivity) of 14.87% (95%CI: 13.9%, 21.7%).

This prevalence exceeds the threshold of 8%, and places our study population in the high prevalence group according to the WHO classification (24). Risk factors independently associated with HBV infection were subjects with HBsAg+ mothers, history of transcutaneous procedures, sharing flat lifestyle, and history of HBV testing.

These figures are lower than that reported in studies carried out in the general population of south-west Chad, which were 22.9% (11) and estimate of 19% by the WHO (25). This may be due to the students' younger age. Most HBV infections in high-prevalence (>8%) countries are the result of mother-to-child transmission (MTCT) or early childhood household horizontal transmission (26) (27). However, sexual, and parenteral HBV infection may occur. Other factors, including percutaneous routes of transmission (barbers, piercing, or traditional practices like excision or circumcision), may also be incriminated in HBV transmission. Of sure, students are less exposed than adults and general population.

HBV infection is still poorly covered by the national health systems, and it is poorly known among the population, as educational campaigns are rare. The lack of universal health coverage (UHC), in Chad and the lack of a national program for the management of hepatitis B infection is a real obstacle to control the epidemic. According to available data, in Chad, about 60,000 people are covered by health insurance, mainly in the private sector (28). In fact, the Chadian government has recently committed a national program to fight against viral hepatitis, but this has not yet been adequately funded.

The prevalence of HBV among students found in this study is higher than that reported in studies from Togo (4.6%) (29), Cameroon (5.6%) (30) and Ethiopia (11.5%) (31), but lower than that from Nigeria (31.5%) (32), while our results are consistent with those reported in Central African Republic (15.5%)(16). As well as birth dose followed by three doses of pentavalent vaccine as part of a national immunization programme, other prevention measures adapted to students (i.e., information about sexuality, sexually transmitted diseases, and risky behavior) are not still implemented in Chad. The student environment may be risky (i.e., drug,

alcohol, and sex temptation etc.). Therefore, in addition to the extreme poverty of this population, students are tempted due to frequent inappropriate environments. Moreover, in the Chadian context, sexual education in the family remains a taboo that has not yet been broken. This lack of education makes students particularly vulnerable and exposes them to major risks such as unprotected sexual activity.

Intra-familial transmission as well as other factors have been mentioned elsewhere as associated with hepatitis B among students (16)(17). In our study, we found no association between a history of HBV infection in the family and HBV positivity. However, although not significant, a family history of HBV infection corresponded to a higher proportion of infected participants (21%).

We also found a significant association between students HBV infection and notion of HBs Ag + mother. The association between HBV infection and the HBV status of mother is coherent with results reported elsewhere. Sub-Saharan Africa is a highly endemic area where perinatal infection is common (33). Same findings were observed in Gambia in 2007, where mother-to-child transmission was responsible for 16% of chronic infections(34) and in Cameroon, where children born from HBsAg positive mothers had a substantial risk of mother-to-child transmission, even despite birth dose vaccination (35). In 2019, the vaccination program of the Ministry of Health reported that less than 50% of children are fully vaccinated. Thus, hepatitis B mother to child transmission is a factor which can favor the spreading of HBV infection. To date, there is no program in place to prevent the transmission of HBV from mother to child In Chad i.e., birth dose HBV vaccination of newborns.

In this study, participants living in roommate accommodation were seven times more likely to acquire HBV infection, this underlines the importance of changing room accommodations habits and built new housing for students in N'Djamena, as

universities in Chad do not have student accommodation. The roommate is an economic solution as it allows sharing daily expenses besides renting. Despite its abundant natural resources, Chad remains one of the poorest countries in the world (36) ; with most of its inhabitants living in poverty, with a national incidence of 46.7% [32]. HBV can survive on environmental surfaces for more than a week. Indirect exposure to the virus can occur through contaminated objects (38). The risk of sharing objects for personal use, e.g., hairbrushes, combs, razors, can cause damage to the skin or mucous membranes and transmit HBV. This type of horizontal transmission is most common in areas of high endemicity, where hygiene is poor(39).

Students who had a history of transcutaneous medical examinations were almost three times more likely to acquire HBV infection than their counterparts [AOR = 2.63, 95% CI = 1.32-5.10, p = 0.005]. In fact, several factors determine the risk of HBV nosocomial transmission. These include invasive surgical procedures [35], failure to follow aseptic techniques and recommended control practices to prevent postoperative infections due to cross-contamination of medical equipment and devices. HBV is the most highly transmissible of the blood-borne viruses that are important in the health care setting. Available data suggest that the risk of transmitting HBV from infected healthcare workers to patients is much higher than for other viruses such as hepatitis C or HIV (41).

The study also showed that the likelihood of being an HBV carrier was twice among students who had history of HBV screening. Our findings are consistent with data reported by Halatoko and al in Togo, who found a statistical association between knowledge of one's HBV status and hepatitis B infection (42).

The participation in the study was voluntary, this may be a limitation of the study. Students who knew they were already infected might be tempted to participate in the study, hoping to receive treatment or to check their serological status. There is a need to focus on management and clinical follow-up for student who were tested positive for HBsAg, and to encourage those who were tested negative to complete the vaccination course. In the current study, all participants were sensitized about HBV and those tested positive were referred to health facilities for follow up.

Strengths and limitations of the study

This is a multicenter study bringing together a mixed population of students from the universities of N'djamena and Emi-Koussi. This reflects the epidemiology of HBV within this community. The study did not include assays for the detection of total anti-HBc antibodies (Ab) and anti-HBc IgM Ab. This could have made it possible to distinguish between acutely infected subjects (with anti-HBc IgM Ab), chronic carriers, and subjects who have recovered from acute or chronic infection (anti-HBc Ab positive).

Conclusion

HBV is an infectious disease of great concern in Chad, and health authorities plan to eliminate it by 2030. The prevalence of HBV found in our study confirm that Chad is a highly endemic country. Having a HBsAg+ mother, living in shared flats, having a history of transcutaneous procedure and of HBV screening were factors independently associated with HBV infection among students. Policies are needed to reduce the incidence and mortality of VHB disease.

Our results highlight the need to reinforce prevention, screening, management, and treatment measures, such as MTCT prevention through pregnant women HBV, birth

dose vaccination of newborns, vaccination part of EPI or catch-up vaccination of children or students and screening the general population. Moreover, as drugs active against HBV, like nucleotide analogs, are now available at low cost, subjects with signs of liver disease should be treated according to WHO recommendations.

Recommendations

In view of our results, a policy to fight against this disease should include the sensitization of at-risk population such as students on the mode of transmission of HBV, introducing hepatitis B vaccination at birth, making full hepatitis vaccination status mandatory before a child starts school, promoting mass screening to identify and treat chronic HBV carriers to reduce transmission in the community, reducing the vaccination cost. The policy should promote a free of charge of HBV screening for pregnant women and the improving of the living conditions the population through decent accommodation including those of students.

List of abbreviations

Anti-HBs: Hepatitis B surface antibody, AOR: Adjusted odd ratio; CI: Confidence interval, COR: Crude odd ratio, EPI: Expanded Program of Immunization, HBsAg: Hepatitis B surface antigen, HBV: Hepatitis B virus; HCC: Hepatocellular carcinoma, WHO: World Health Organization and UHC: Universal health coverage.

Acknowledgements

We are thankful to laboratory technicians of Hôpital de la Paix in N'djamena who performed the blood sampling and tests. We express our gratitude to data collectors and supervisors of the survey. We thank the deans of Cheikh Anta Diop University in Dakar for their guidance. We are thankful to the secretaries of the students who have

facilitated the sensitization of students to participate in the study and the students who participated in the study.

Availability of data and materials

The datasets generated and/or analyzed during the current study are available in the Mendeley repository here .

Conflicts of interest

The authors have declared that no competing interests exist.

Funding

The author(s) received no specific funding for this work.

Author Contributions

Conceptualization: Nalda Debsikréo, Françoise Lunel-Fabiani.

Data curation: Nalda Debsikréo, Birwé Léon Mankréo, Azoukalné Moukénet.

Formal analysis: Nalda Debsikréo, Birwé Léon Mankréo, Azoukalné Moukénet.

Funding acquisition: Nalda Debsikréo.

Investigation: Nalda Debsikréo, Merwa Ouangkake, Nathan Mara, Ali Mahamat Moussa.

Methodology: Nalda Debsikréo, Birwé Léon Mankréo, Azoukalné Moukénet, Ali Mahamat Moussa Ndèye Coumba Toure-Kane, Françoise Lunel-Fabiani.

Project administration: Nalda Debsikréo, Merwa Ouangkake, Nathan Mara, Ali Mahamat Moussa.

Software: Nalda Debsikréo, Birwé Léon Mankréo, Azoukalné Moukénet.

Laboratory: Nalda Debsikréo, Merwa Ouangkake, Nathan Mara.

Validation: Nalda Debsikréo; Birwé Léon Mankréo, Azoukalné Moukénet, Merwa Ouangkake, Nathan Mara, Ali Mahamat Moussa, Ndèye Coumba Toure-Kane, Françoise Lunel-Fabiani.

Visualization: Nalda Debsikréo, Birwé Léon Mankréo, Azoukalné Moukénet.

Writing – original draft: Nalda Debsikréo

Writing – review & editing: Nalda Debsikréo, Birwé Léon Mankréo, Azoukalné Moukénet, Merwa Ouangkake, Nathan Mara, Ali Mahamat Moussa, Ndèye Coumba Toure-Kane, Françoise Lunel-Fabiani.

References

1. Stanaway JD, Flaxman AD, Naghavi M, Fitzmaurice C, Vos T, Abubakar I, et al. The global burden of viral hepatitis from 1990 to 2013: findings from the Global Burden of Disease Study 2013. The Lancet. 10 sept 2016;388(10049):1081‑8.

2. Selzer L, Zlotnick A. Assembly and Release of Hepatitis B Virus. Cold Spring Harb Perspect Med. déc 2015;5(12): a021394.

3. Global hepatitis report, 2017 https://www.who.int/publications-detail-redirect/9789241565455

4. Schweitzer A, Horn J, Mikolajczyk RT, Krause G, Ott JJ. Estimations of worldwide prevalence of chronic hepatitis B virus infection: a systematic review of data published between 1965 and 2013. Lancet Lond Engl. 17 oct 2015;386(10003):1546‑55.

5. Polaris Observatory Collaborators. Global prevalence, treatment, and prevention of hepatitis B virus infection in 2016: a modelling study. Lancet Gastroenterol Hepatol. juin 2018;3(6):383‑403.

6. World Hepatitis Day, 28 July 2021. WHO | Regional Office for Africa. 2023 https://www.afro.who.int/regional-director/speeches-messages/world-hepatitis-day-28-july-2021

7. Lu P jun, O'Halloran AC, Williams WW, Nelson NP. Hepatitis B vaccination coverage among adults aged ≥ 18 years traveling to a country of high or intermediate endemicity, United States, 2015. Vaccine. 25 avr 2018;36(18):2471‑9.

8. Janevska D, Chaloska-Ivanova V, Janevski V. Hepatocellular Carcinoma: Risk Factors, Diagnosis and Treatment. Open Access Maced J Med Sci. 15 déc 2015;3(4):732‑6.

9. Abbas Z, Abbas M. Challenges in Formulation, and Implementation of Hepatitis B Elimination Programs. Cureus. 24 avr. 2021 ;13(4) : e14657.

10. Tchad - Plan national de développement sanitaire (2018-2021) (PNDS3). https://www.ilo.org/dyn/natlex/natlex4.detail?p_lang=fr&p_isn=111135&p_country=TCD&p_count=155

11. Suesstrunk J, Djongali FB. Hepatitis B virus prevalence in rural areas in south-west Chad. Trop Doct. oct 2017;47(4):374‑7.

12. Bessimbaye N, Moussa AM, Mbanga D, Tidjani A, Mahamat SO, Ngawara MN, et al. [Seroprevalence of HBs Ag and of anti-HCV antibodies among HIV infected people in N'Djamena, Chad]. Bull Soc Pathol Exot 1990. déc 2014;107(5):327‑31.

13. Yandai FH, Traore KA, Moussa AM, Ouoba BL, Ouoba JB, Bolti MA, et al. Prevalence and Diversity of Hepatitis Virus Markers among Patients with Acute Febrile Jaundice in Chad. Microbiol Res. Déc 2021 ;12(4) :878‑87.

14. Zampino R, Boemio A, Sagnelli C, Alessio L, Adinolfi LE, Sagnelli E, et al. Hepatitis B virus burden in developing countries. World J Gastroenterol. 14 nov. 2015 ;21(42) :11941‑53.

15. Tao I, Compaoré TR, Diarra B, Djigma F, Zohoncon TM, Assih M, et al. Seroepidemiology of Hepatitis B and C Viruses in the General Population of Burkina Faso. Hepat Res Treat. 2014; 2014:781843.

16. Komas NP, Baï-Sepou S, Manirakiza A, Léal J, Béré A, Le Faou A. The prevalence of hepatitis B virus markers in a cohort of students in Bangui, Central African Republic. BMC Infect Dis. 29 juill 2010;10(1):226.

17. Njoku OO. Prevalence of assymptomatic hepatitis B virus among sexually active youths in a rural community of Ebonyi state, Southeast Nigeria. Int J Infect Dis. 1 avr 2016; 45:450‑1.

18. Ferrinho P, Dramé M, Tumusiime P. Perceptions of the usefulness of external support to immunization coverage in Chad: an analysis of the GAVI-Alliance cash-based support. Pan Afr Med J. 7 juin 2013 ;15 :44.

19. tcd_rpt_annuairesstatistiquessanitaires_ministeresante_2017.pdf https://www.humanitarianresponse.info/sites/www.humanitarianresponse.info/files/documents/files/tcd_rpt_annuairesstatistiquessanitaires_ministeresante_2017.pdf

20. WATHI. Politique nationale de santé 2016-2030, ministère de la Santé publique du Tchad, 2016. WATHI. 2021 https://www.wathi.org/wathinote-election-tchad-situation-sanitaire/politique-nationale-de-sante-2016-2030-2016/

21. La formation à l'université de N'Djaména [https://archive.wikiwix.com/cache/index2.php?url=https%3A%2F%2Fwww.universite-ndjamena.td%2Fformation#federation=archive.wikiwix.com

22. L'Université. Université Emikoussi. 2022 https://universite-emikoussi.net/luniversite/

23. Dembele B, Affi-Aboli R, Kabran M, Sevede D, Goha V, Adiko AC, et al. Evaluation of Four Rapid Tests for Detection of Hepatitis B Surface Antigen in Ivory Coast. J Immunol Res. 26 juin 2020; 2020:6315718.

24. Organization WH. Guidelines for the prevention care and treatment of persons with chronic hepatitis B infection: Mar-15. World Health Organization ; 2015.

25. Journée mondiale contre l'Hépatite : l'OMS lance un appel pour des actions de lutte et de prévention permanentes. OMS | Bureau régional pour l'Afrique. 2022

https://www.afro.who.int/fr/countries/chad/news/journee-mondiale-contre-lhepatite-loms-lance-un-appel-pour-des-actions-de-lutte-et-de-prevention

26. Kiros KG, Goyteom MH, Tesfamichael YA, Mekonen HH, Gebru TH, Gebrehiwot TG, et al. Seroprevalence of Hepatitis B Virus Infection, Mother-To-Child Transmission, and Associated Risk Factors Among Delivering Mothers in Tigray Region, Northern Ethiopia: a Cross-Sectional Study. Infect Dis Ther. déc 2020;9(4):901‑11.

27. Lok ASF, McMahon BJ. Chronic hepatitis B: Update 2009. Sept 2009 [cité 25 mars 2023] ; http://deepblue.lib.umich.edu/handle/2027.42/63599

28. La Couverture santé universelle, une des priorités de l'agenda politique des autorités nationales tchadiennes. OMS | Bureau régional pour l'Afrique. 2023 https://www.afro.who.int/fr/news/la-couverture-sante-universelle-une-des-priorites-de-lagenda-politique-des-autorites

29. Ekouevi DK, Thomas A, Sewu D, Lawson-Ananissoh L, Tchounga B, Salou M, et al. Prevalence of Hepatitis B among Students from the University of Lomé, Togo in 2015. Open J Epidemiol. 8 août 2017 ;7(3) :262‑72.

30. servais albert fiacre E. Statut Vaccinal Contre le Virus de l'Hépatite Virale B et portage de l'Antigène Hbs chez les Etudiants en Médecine et en Pharmacie de l'Université de Douala au Cameroun. Health Sci Dis. 1 janv 2014.

31. Tesfa T, Hawulte B, Tolera A, Abate D. Hepatitis B virus infection and associated risk factors among medical students in eastern Ethiopia. PLOS ONE. 19 févr 2021;16(2): e0247267.

32. Tula MY, Iyoha O. A Cross-sectional Study on the Sero-prevalence of Hepatitis B Surface Antigen (HBsAg) among Apparently Healthy Students of a Tertiary Institution in North-Eastern Nigeria. Int J Trop Dis Health. 10 janv 2015; 7:102‑8.

33. Shimakawa Y, Toure-Kane C, Mendy M, Thursz M, Lemoine M. Mother-to-child transmission of hepatitis B in sub-Saharan Africa. Lancet Infect Dis. 1 janv 2016;16(1):19‑20.

34. Shimakawa Y, Lemoine M, Njai HF, Bottomley C, Ndow G, Goldin RD, et al. Natural history of chronic HBV infection in West Africa: a longitudinal population-based study from The Gambia. Gut. 1 déc 2016 ;65(12) :2007‑16.

35. Shimakawa Y, Veillon P, Birguel J, Pivert A, Sauvage V, Guillou-Guillemette HL, et al. Residual risk of mother-to-child transmission of hepatitis B virus infection despite timely birth-dose vaccination in Cameroon (ANRS 12303): a single-centre, longitudinal observational study. Lancet Glob Health. 1 avr 2022;10(4): e521‑9.

36. Tchad - Vue ensemble. World Bank. https://www.banquemondiale.org/fr/country/chad/overview

37. INSEED-TCHAD - Pauvreté. https://www.inseed.td/index.php/blog-with-right-sidebar/cle-economique/258-pauvrete

38. Bond WalterW, Favero MartinS, Petersen NormanJ, Gravelle CliftonR, Ebert JamesW, Maynard JamesE. SURVIVAL OF HEPATITIS B VIRUS AFTER DRYING AND STORAGE FOR ONE WEEK. The Lancet. 7 mars 1981;317(8219):550‑1.

39. Meheus A. Teenagers' lifestyle and the risk of exposure to hepatitis B virus. Vaccine. 18 févr 2000;18 Suppl 1: S26-29.

40. Sugimoto S, Nagakubo S, Ito T, Tsunoda Y, Imamura S, Tamura T, et al. A case of acute hepatitis B related to previous gynecological surgery in Japan. J Infect Chemother Off J Jpn Soc Chemother. juin 2013;19(3):524‑9.

41. Elseviers MM, Arias-Guillén M, Gorke A, Arens HJ. Sharps injuries amongst healthcare workers: review of incidence, transmissions, and costs. J Ren Care. sept 2014;40(3):150‑6.

42. Halatoko WA, Patassi A, Yanogo P, Banla LI, Koba A, Issa Z, et al. Risk factors of hepatitis B virus surface antigen carriage and serological profile of HBsAg carriers in Lomé Togo, 2016. BMC Public Health. 8 janv 2019;19(1):32.

Supporting information

S1 File. Questionnaire (docx)

381 S2 File. Checklist (docx)

S1 File. Questionnaire

Click here to access/download
Supporting Information
S1 Questionnaire.docx

S2 File. Checklist

Click here to access/download

Supporting Information

S2 Check list.docx

Title: Prevalence of hepatitis B virus infection and associated ~~risk~~ factors among students in N'Djamena, Chad.

Short title: Hepatitis B virus and associated ~~risk~~ factors among Chadian studens

Authors: Nalda Debsikréo[1,2]*, Birwé Léon Mankréo[1], Azoukalné Moukénet[1,3], Merwa Ouangkake[4], Nathan Mara[4], Ali Mahamat Moussa[3,5], Ndèye Coumba Toure-Kane[1,2], Françoise Lunel-Fabiani[6]

Affiliations :

[1]Cheikh Anta Diop University, Dakar, Senegal; [2]Institut de Recherche en Santé, de Surveillance Épidémiologique et de Formation, Dakar, Senegal; [3]University of N'Djamena, N'Djamena, Chad; [4]Hôpital de la Paix, N'Djamena, Chad; [5]Centre Hospitalier Universitaire la Référence, N'Djamena, Chad; [6] Centre Hospitalier Universitaire Angers, BAT IBS - 4 rue Larrey- 49000 ANGERS, Laboratoire HIFIH, UFR Santé département Médecine, SFR 4208-UPRES EA3859, Université d'Angers, 49045 Angers Cedex 01, France

Corresponding author: * ndaldaeliane@gmail.com (ND)

Abstract

Background~~:~~

Hepatitis B virus ~~Viral hepatitis B~~ (HBV) is a major public health ~~threat~~. In Chad, HBV screening is not systematic, due to the low interest among health care ~~of~~ providers or frequent shortages of ~~supplies~~ medical supplies. Thus, most people at risk such as students are unaware of their status. ~~However~~ Many people with chronic HBV infection are asymptomatic~~, many persons chronically infected with HBV have no or low liver disease~~. This study ~~aims~~ aimed to determine the prevalence of HBV and identify ~~risk~~ factors among students in N'Djamena, Chad.

Methods:

A cross-sectional survey study at the Universities of N'Djamena and Emi Koussi was conducted in from 3 to 23 July 2021. Students were including a consecutive sampling . 457 students randomly selected within the universities of N'Djamena and Emi Koussi. A structured questionnaire was used to collect data on sociodemographic characteristics and other risk factors. Blood samples were collected and serum was tested for hepatitis B surface antigen (HBsAgHBsAg testing was performed using the Determine HBsAg® Rapid Test Kit and confirmed by Abbott Architect i1000SR analyzer based on chemiluminescent microparticle immunoassay (CMIA).. Descriptive analyzes and binary and multivariate logistic regressions were used to examine the relationship between dependent variables and sociodemographic including other risk associated factors. Odds ratios (ORs) and their 95% confidence intervals (CIs) were used as indicators of the association. A p value of 0.05 was used to indicate the statistical significance of the relationships.

Formatted: Font: Not Bold

Results~~:~~

~~The HBsAg seroprevalence was 14.87%. Students aged between 26-35 years (OR= 0.90, 95% CI: 0.42-1.80; p= 0.032), who were born from HBsAg positive mothers (OR= 0.48, 95% CI: 0.08-3.84; p = 0.027), who lived with a roommate (OR= 5.42, 95% CI: 1.55-25.6; p= 0.025) or with family (OR= 2.69, 95%CI: 0.90-11.7; p= 0.025) or had a history of hepatitis (OR= 0.46, 95% CI : 0.25-0.86, p=0.015) were less likely to contract HBV infection.~~A total of 457 students aged between 19 to 59 years were included in the study. The prevalence of HBV infection was 14.87% (95% CI =13,9–21,7%). Most students, 91%, were in the 20–30-year age group, 64.9% reported unprotected sex, 53.6% reported multiple sexual partners and 45.7% of students had no knowledge of hepatitis B. Having a HBsAg+ mother (AOR: 2.60; 95%CI: 1.18-5.80, p=0.018), a transcutaneous procedure history (AOR: 2.63 95%CI: 1.32-5.10, p=0.005), being in a roommate (AOR: 7.19, 95%CI 2.08-33.79, p=0.004) and having history of HBV screening (AOR: 2.03, 95%CI: 1.11-3.65, p=0.019) were associated with a higher risk of HBV infection.

Conclusion~~:~~

The 14.87 ~~prevalence~~% prevalence rates found in our study confirm that Chad is a highly endemic country. ~~of hepatitis B is high among Chadian students~~. ~~There is an urgent need to provide screening tests, HBV vaccination to improve the living conditions for students in Chad. This study highlights some solutions to improve the situation of hepatitis B in Chad and suggests policies to eliminate viral hepatitis~~ ~~.~~. Having a HBsAg+ mother, living in a shared flat, having a history of transcutaneous procedure and a history of HBV screening were factors independently associated with HBV infection among students. Policies, such as routine screening of the population, birth dose vaccine and access to treatment are needed to reduce the incidence and mortality of HBV related diseases.

Formatted: Font: Not Bold

KEY WORDS: hepatitis B, HBsAg, -seroprevalence, associated factors student, Chad.

Formatted: Font: Not Italic

Introduction

~~Viral H~~hepatitis B is a major public health, ~~threat~~ despite the availability of a prophylactic vaccine, and one of the leading causes of death worldwide (1). Hepatitis B virus (HBV) is a DNA, partially circular virus belonging to the Hepadnaviridae family. It is a small, enveloped virus responsible for acute, sometimes fulminant hepatitis and for chronic hepatitis. It has also an oncogenic potential (2)~~and one of the leading causes of death worldwide~~. Chronic HBV infection is widespread ~~Around~~ 900.000 deaths per year are related to HBV infection ~~due to hepatitis B virus infection~~(3) ~~[1]. The global seroprevalence of hepatitis B surface antigen (HBsAg) has been estimated as high as~~ and it prevalence is 3.6%~~, with the highest endemicity areas located in the African and Western Pacific regions respectively 8.8% and 5.3% [2].~~

Despite a lack of accurate data on viral hepatitis B ~~I~~n Africa, it is estimated that its prevalence is one of the highest in the world. Sub-Saharan Africa has a high prevalence of HBsAg exceeding 8% in general population (5), and ~~where hepatitis B is a silent epidemic,~~ more than 90 million~~ infected~~ people are HBV infected , ~~live in the sub-Saharan region, which~~ representing 26% of the total number of affected people ~~affected in the~~ worldwide ~~[3]~~ . (6).

Since chronic hepatitis B infection remains silent for a long time and is unnoticed, the population remains under-informed about possible prevention and about the risks and consequences of hepatitis B infection. This contributes to lower health coverage and an increased risk of HBV infection in the population (7). with the risk of developing cirrhosis or hepatocellular carcinoma (HCC) (8).

~~Most people chronically infected with HBV have no active liver disease and are defined as inactive carriers or as chronic infection, according to the European Association for the Study of the Liver (EASL) classification [4]. However, subjects with persistent infection may develop cirrhosis, liver failure and/or hepatocellular carcinoma [5].~~ Low-income countries have a higher burden of HBV infection, however testing and access to treatment remain limited in these countries~~-~~More than 124,000 Africans die each year as a result ~~from the consequences~~ of

undetected and untreated hepatitis ~~[3]~~. (6). Therefore, screening people who have not yet been diagnosed is a major challenge in eliminating hepatitis B (9).

In Chad, ~~data on~~ HBV prevalence data are available only ~~available~~ for ~~among~~ specific population subgroups and regions due to lack of systematic research (10) ~~or frequent shortages of supplies [6]~~. A study conducted in Chad reported a prevalence of HBsAg of 22.9% in the general population of southwestern Chad (11) ~~[7]~~ and 13.5% in the HIV population of N'Djamena (12) ~~[8]~~. A recent study of patients with acute jaundice in Chad found an HBsAg prevalence of 10.3% (13) ~~[9]~~. HBV infection is therefore a significant public ~~an important~~ public health problem that requires active surveillance in Chad.

Formatted: Space After: 0 pt, Line spacing: Double

Formatted: Font: (Default) Calibri, 11 pt

~~However, there are few data on the epidemiology of HBV in the general population.~~

HBV can be transmitted from biological fluids of infected individuals, especially blood and sexual secretions. ~~In highly endemic countries, hepatitis B is commonly T~~transmitted of HBV is vertically ~~from mother to child at birth~~ or horizontally in the perinatal period in areas of high endemicity (14).~~during the first five years of life [10]. Infants infected during the perinatal period have 3.74 times (OR=3.74, 95% CI: 0.97–14.39) risk of becoming chronic carriers [11]~~. Parenteral transmission occurs through blood transfusion, blood exposure accidents among health professionals and the use of non-sterile equipment for tattooing, piercing, acupuncture, and intravenous drug injections and transmission may also occur through unprotected sexual intercourse (3) (15).

Students, who are typically the youngest, apparently healthy, and sexually active, engage in risky behaviours such as alcohol abuse, multiple sexual partners and are exposed to sexually transmitted diseases. In addition, some studies found that family history of HBV, sexual activity and socioeconomic conditions were the main risk factors for HBV infection among students in Bangui (16) and asymptomatic adolescents in Nigeria (17).

~~. These risk factors are the main causes and most frequent routes of transmission [12]. Moreover, due to the lack of systematic screening among patients attending in health care facilities [6] , most people, including people at risk such as students, are unaware of their HBV infection serological~~

~~status and do not benefit of appropriate management and treatment. Indeed, students are generally young, in good apparent health and sexually active, which puts them at risk of HBV infection. In addition, they spend more time in a crowded environment (amphitheater, dwellings, etc.) and HBV transmission may occur via saliva and skin exudates [13] .~~

Since the 2000s, the HBV vaccine has been introduced into the expanded immunization program (EPI) of ~~in~~ several ~~countries~~ in sub-Saharan Africa countries to prevent HBV transmission during childhood. In Chad, the HBV vaccine was introduced into the National EPI in 2008 (18) [~~1~~4]. ~~However,~~ D~~d~~espite this policy, the prevalence of HBV infection ~~prevalence~~ continues to increase, especially ~~grow, particularly~~ in the population over 15 years of age (~~the~~ incidence in subjects over 15 years of age ~~old~~ is 26.46% versus a national incidence of 13.62%) (19). ~~[6]~~.

This could hamper ~~observation may inhibit~~ the achievement ~~of the objectives~~ of the National Health Plan targets ~~which is~~ to eliminate major ~~the main neglected~~ tropical diseases such as viral hepatitis, water-borne diseases and other communicable diseases by 2030 (20) ~~[14]~~ . ~~Moreover, few~~ S~~s~~tudies ~~have been performed~~ on the burden of HBV infection among the ~~liver disease in at-risk populations such as~~ student ~~s~~ population and factors associated with hepatitis B are limited in the community, making it necessary to conduct studies to guide policies in preventing this scourge in Chad. Therefore, Th~~i~~us study~~, the~~ aimed ~~of the study was~~ to determine the prevalence of HBV and to identify the ~~risk~~ factors associated with HBV infection among students in~~of the Universities of~~ Ndjamena~~–~~, Chad~~and Emi Koussi. Identifying these risk factors for HBsAg carriage with appropriate management would reduce the disease burden. Knowledge of the HBV infection risk factors would also guide policies aimed at reducing the HBV burden~~.

Materials and Methods

Study design and framework

A cross-sectional study was conducted in the universities of Ndjamena and Emi Koussi from July 3 to 23, 2021. The study setting included public and private ~~institutions as well as~~ universities. The universities of N'Djamena and Emi Koussi were selected because they have the largest number of students and N'Djamena is the city with the highest incidence of HBV (42.16%)(19)~~[6]~~ in Chad. The University of N'Djamena is also the largest in the country with 16,142 students spread over 04 campuses and 7 faculties and The Emi Koussi university is one of largest private universities with around 3,000 students and 02 faculties spread over two campuses (22). ~~[15] .~~

~~Sampling~~ Sample size determination and sampling technique

A list of all faculties of the two largest universities of N'Djamena were obtained from its respective administrations. Based on number of students in faculties of the two largest universities of N'Djamena, students who volunteered to participate in the study were enrolled according to consecutive sampling.

The sample size was calculated using the single population following formula $n= (Z\alpha/2)^2 P(1-P) / i^2$ [16]. Given that there is ~~has been~~ no similar study in a student environment in Chad, a prevalence of 50% ~~(close to 42.12%, the incidence of HBV in N'djamena),~~ a confidence interval of 95% ($Z\alpha/2$ = 1.96), an error margin of 5% and a provision for non-responses of 10% were used. Thus, the sample size obtained was 422 students. The sample size calculated was distributed proportionally to the number of students in faculties. (Faculty of Languages, Letters, Arts and Communication (75 students), Faculty of Educational Sciences (80 students), Faculty of Exact and Applied Sciences (50 students), Faculty of Economics and Management (88 students), Faculty of Human and Social Sciences (75 students) and University of Emi Koussi (50 students)).

Formatted: Heading 2, Left, Line spacing: 1.5 lines

Formatted: Font: (Default) Cambria, 13 pt, Not Bold, Font color: Custom Color(RGB(46,117,181))

The sample size calculated was distributed proportionally to the number of students in faculties (Faculty of Languages, Letters, Arts and Communication (75 students), Faculty of Educational Sciences (80 students), Faculty of Exact and Applied Sciences (50 students), Faculty of Economics and Management (88 students), Faculty of Human and Social Sciences (75 students) and University of Emi Koussi (50 students)).

~~selected purposely based on the number of students with Faculty of Languages, Letters, Arts and Communication (75 students), Faculty of Educational Sciences (80 students), Faculty of Exact Sciences and Applied (50 students), Faculty of Economics and Management (88 students), Faculty of Human and Social Sciences (75 students) and University of Emi Koussi (50 students).~~

~~Within each faculty, a random sampling technique was applied to select study participants until the required number was reached. A first student was selected for each faculty then gradually other volunteers were enrolled until the required number was reached.~~

~~All volunteer students from the universities of N'Djamena and Emi Koussi were included in the study until the required sample was reached. Students who were not from these universities and who were unable to give informed consent were excluded from the study.~~

Data collection method

A structured questionnaire was used to collect information on socio-demographic characteristics (age, sex, marital status, place of origin, faculty and level of study) and risk behaviors such as: the number of sexual partners, blood transfusion, surgery, hospitalization, HBsAg status of the mother, HBsAg status of family members, transcutaneous exposure such as acupuncture, drug use, prisoners, unprotected sex, alcohol, sharp object wounds, mode of residence, density of residence, cloths sharing, sleeping arrangements sharing.

~~Blood sample collection~~ Laboratory data processing

A 5ml venous blood sample was collected by a lab technician in an EDTA tube after obtaining informed consent from the study participants. The blood samples were labeled with unique identification numbers and coagulated. Serum was separated by centrifugation at 3000 rpm for 5 min

and placed into Eppendorf tubes. HBsAg was searched in sera samples using ALERE Determine® HBsAg test, which is based on the principle of immunochromatography. This is a qualitative test for the rapid detection of HBsAg, with very good sensitivity and specificity (100%) [20]. The analyses were performed in accordance with the manufacturers' instructions.

Positive results were confirmed by Abbott Architect i1000SR analyzer (Abbott Diagnostics, Abbott Park, IL, USA) based on chemiluminescent microparticle immunoassay (CMIA) for the qualitative detection of HBsAg. HBsAg testing was done an ARCHITECT HBsAg Qualitative assay according to the manufacturer's instructions. After HBs Ag screening test, sera samples were frozen and kept at -80°C in the biobank laboratory of the national reference hospital.

Formatted: Space Before: Auto, After: Auto, Line spacing: 1.5 lines, Pattern: Clear (White)

Formatted: Font: (Default) Cambria, 12 pt

~~Selection of variables~~ Socio-demographic data collection

Formatted: Font: (Default) Cambria, 13 pt, Not Bold, Font color: Custom Color(RGB(46,117,181))

Formatted: Heading 2, Left, Line spacing: 1.5 lines

~~We assessed~~ HBV ~~sero~~prevalence was calculated on the number of ~~as~~ blood sample tested positive for the HBV test. ~~and considered as HBsAg positive.~~ Regarding the risk factors, potential variables retained were the student life history including number of sexual partners, blood transfusion, surgery, hospitalization, HBsAg status of mother, HBsAg status of family members, transcutaneous exposure, acupuncture, drug use, prisoners, unprotected sex, alcohol, sharp objects wounds, living arrangements, living density, shared clothing, shared sleeping arrangements, history of hepatitis B. In addition to these variables, sociodemographic characteristics of participants including their medical/not medical history were included as risk factors.

~~Biological analysis~~

~~For the detection of HBsAg, the ALERE Determine® HBsAg test was used. Its principle is based on a rapid sandwich-type reaction for the detection of HBsAg. The plastic protection of each test was removed. Then, 50 µl of serum was dispensed onto the sample deposit area using a pipette. After 15 min, results were interpreted based on the appearance of color bands. The red bars appear in the control window (labeled "Control") and the patient window (labeled "Patient") on the strip. Any red color visible in the patient window was interpreted as a positive result (a bar). A red bar appears in~~

the control window (annotated "Control"), the red bar in the patient window (annotated "Patient") not appearing on the strip was interpreted as a negative result. If the red bar did not appear in the strip control window and even if a red bar appeared in the strip patient window, the result was considered as invalid, and the test was repeated.

Data analysis

Data were entered into an Excel database and analyzed using R version 4.1.2 (2021-11-01) software. A descriptive analysis and logistic regressions were used to examine the relationship between dependent variables and was performed on participants' social and demographic characteristics and others risk factors including other risk factors. Quantitative variables were expressed as the mean ± SD. Categorical variables were expressed as numbers (percentages). The independent t-test was used to compare the means of continuous variables. When normal distribution or equality of variance could not be assumed, the Mann-Whitney U test was used. stratified by HBV serological status. The comparison of proportions was assessed by means of the Chi-square test. In both cases, the level of significance was set at $p < 5\%$. As multivariate analysis, a logistic regression analysis was conducted to identify the factors associated with HBV serological status among students. A Chi-square test and a Student t-test were performed to assess any significant difference in socio demographics characteristics between HBV students' status. As multivariate analysis, a logistic regression analysis was conducted to identify the factors associated with HBV serological status among Chadian students. Odds ratios (ORs) were calculated to check statistical associations between the dependent and independent variable. All variables in the study were initially tested for association with HBV status using a binary logistic regression model. Variables with $P < 0.3$ in the univariate analysis were included in the multivariate logistic regression analysis. Those showing a significant statistical association were added to the multivariable analysis model to assess whether the association existed after controlling against all the rest of the variables. Adjusted Odds ratios (AORs) were calculated to check statistical associations between the dependent and independent variable after controlling against all the rest of the variables. All variables that were less than 20% significant (i.e., with a p-value < 20%) in univariate analysis were included in the initial multivariate logistic regression model. The forced variables, i.e., age and sex, were also included in the multivariate analysis,

even though they did not have a p-value < 20% in the univariate analysis. To obtain the final multivariate model, a logistic regression was then performed. Variables were selected according to the statistical criteria of selection by descending stepwise procedure. The final model was selected with the exclusion of the variables that were not significant, using Akaike's information criterion as an index of parsimony. Variables with a p-value ~~A~~ $P < 0.05$ were~~as~~ considered statistically significant. A 95% confidence interval were calculated for all odds ratios.

~~Ethics approval and consent to participate~~ Ethical consideration

The study was approved by the National Bioethics Committee of Chad (N° 201/PR/MESRI/DG/CNBT/2020, November 8, 2020) and the UCAD Research Ethics Committee (CER /UCAD/AD/MSN/050/2020, December 07, 2021). ~~Prior to the study,~~ written ~~-~~informed consent was obtained from all participants for inclusion in the study .~~in accordance with the approval of the Chad National Bioethics Committee and the UCAD Research Ethics Committee~~. The objectives of the survey were presented to the deans of the faculties concerned. Each participant was informed of their serological results and advised on the possible consequences of HBV infection and, if necessary, measures to be taken. HBV-positive people have been referred to the focal point of the program fight against hepatitis in Chad for follow-up and advice.

Inclusivity in global research

Additional information regarding the ethical, cultural, and scientific considerations specific to inclusivity in global research is included in the Supporting Information (S2 Checklist).

Results

~~Sample characteristics~~

Out of the ~~A~~ total of 457 students who participated ~~were enrolled~~ in this study. ~~The socio-demographic characteristics of the participants are presented in Table 1. Seventy-six percent of participants were male, 75% were under 25 years old and 76% were single. There was no association between these sociodemographic characteristics and the HBV serology status~~ Age ranged from 19 to 59 years. The mean age of the students was 24 ±3 SD years and 91% of student were between the age group of 20 and 30 years. Most of participants to the study were from University of N'djamena (407, 89%). Around three out of four study participants were single (76%). (Table 1).

Table 1: Socio-demographic characteristics of study participants ~~according to their serological status, frequency (%)~~

~~*Variables*~~		~~*Overall, N = 457*~~[1]	~~*HBV -, N = 389*~~[1]	~~*HBV +, N = 68*~~[1]	~~*p-value*~~[2]
~~Age~~		~~24.0 (22.0, 25.0)~~	~~24.0 (22.0, 26.0)~~	~~23.5 (22.0, 25.0)~~	~~0.2~~
	~~<25~~	~~343 (75%)~~	~~287 (74%)~~	~~56 (82%)~~	
	~~26-35~~	~~94 (21%)~~	~~82 (21%)~~	~~12 (18%)~~	
	~~>35~~	~~20 (4.4%)~~	~~20 (5.1%)~~	~~0 (0%)~~	
~~Sex~~					~~>0.9~~
	~~Female~~	~~109 (24%)~~	~~93 (24%)~~	~~16 (24%)~~	
	~~Male~~	~~348 (76%)~~	~~296 (76%)~~	~~52 (76%)~~	
~~Marital Status~~					~~>0.9~~
	~~Single~~	~~348 (76%)~~	~~297 (77%)~~	~~51 (76%)~~	
	~~Married~~	~~107 (24%)~~	~~91 (23%)~~	~~16 (24%)~~	
	~~Unknown~~	~~2~~	~~1~~	~~1~~	
~~Level of study~~					~~0.2~~
	~~Bachelor 1~~	~~141 (31%)~~	~~113 (29%)~~	~~28 (41%)~~	
	~~Bachelor 2~~	~~143 (31%)~~	~~123 (32%)~~	~~20 (29%)~~	
	~~Bachelor 3~~	~~168 (37%)~~	~~148 (38%)~~	~~20 (29%)~~	
	~~Master & PhD~~	~~5 (1.1%)~~	~~5 (1.3%)~~	~~0 (0%)~~	

~~[1]Median (IQR); n (%), [2]Wilcoxon rank sum test; Fisher's exact test; Pearson's Chi-squared test~~

Characteristic	Category	Frequency	Percent (%)

**Age**	<20 Ans	8	1.8
	20 - 30	417	91
	30- 40	17	3.7
	> 40	15	3.3
**Level of study**	Bachelor1	141	31
	Bachelor2	143	31
	Bachelor3	168	37
	Master & PhD	5	1.1
**Marital Status**	Married	109	24
	Single	348	76
**Sex**	Female	109	24
	Male	348	76
**Site**	FLLAC	75	16
	FSE	99	22
	FSEA	50	11
	FSEG	100	22
	FSHS	83	18
	UEK	50	11

Note: FLLAC (Faculty of Languages, Letters, Arts and Communication), FSE (Faculty of Educational Sciences), FSEA (Faculty of Exact and Applied Sciences), FSEG (Faculty of Economics and Management), FSHS (Faculty of Humanities and Social Sciences), UEK (University of Emi-Koussi).

Socio-cultural and behavioral exposure status among participants

Of the 457 participants, 64.9% (297/457) reported having unprotected sex, 53.6% (245/457) reported having multiple sexual partners, while 45.7% (209/457) of participants were unaware of hepatitis B and 76.5% (350/457) had no history of HBV screening.

Most of the participants - 77.2% (353/457) - lived in a family environment, 53.6% (245/457) lived in a dwelling with more than two persons per room and 60.6% (277/457) reported sharing a bed. Furthermore, 38.2% (175/457) also shared sharp objects **(Table 2).**

Prevalence of HBV and risk factors associated with HBsAg carriage

Of all the total students participating in the study, 68/457 (14.87%(95% CI = 13.9-21.7%)) were tested positive for hepatitis B surface antigen(HBsAg)) (Table 2). The proportion of HBsAg positive cases was significantly higher among students with an HBsAg positive

mother than among those with an HBsAg negative mother (29 vs 13%). There was a significant difference (p= 0.004) between students who lived alone (5.9%) and those who lived with roommates (28 %). Students with a history of HBV screening were also significantly more often HBsAg positive (2 vs 12%) followed by those with a family history of HBV infection (21 vs 13 %), or a history of jail stay (19 vs 15 %) and those who had transfusion (16 vs 15 %) Regarding the history of the students participating in the study, among students who had HBsAg-positive mothers, 29% were affected by HBV infection, as were students who lived in roommates (28%) and students with a known history of hepatitis B testing (23%).

Overall, few students who participated in the study had risk factors such as blood transfusion (16%), hospitalization (14%), family members carrying HBsAg+ (21%), transcutaneous exposure (14%). Moreover, the prevalence of HBV does not vary significantly with these risk factors (Table 2).

Table 2: **Estimated prevalence of HBsAg**

life history of study participants and their serological status, frequency (%)

Variables	Category	HBV - N (%)	HBV + N (%)	Overall, N= 457[1]	p-value[2]
**Numbers of sexual partners**					0.7
	Many	213 (85%)	39 (15%)	252 (100%)	
	One	176 (86%)	29 (14%)	205 (100%)	
**Knowledge of hepatitis B**					0.9
	No	179 (86%)	30 (14%)	209 (100%)	
	Yes	180 (86%)	29 (14%)	209 (100%)	
**Transfusion**					0.8
	No	341 (85%)	58 (15%)	399 (100%)	
	Yes	37 (84%)	7 (16%)	44 (100%)	
**Surgery**					0.056
	No	367 (84%)	68 (16%)	435 (100%)	
	Yes	21 (100%)	0 (0%)	21 (100%)	
**Hospitalization**					0.8
	No	297 (85%)	52 (15%)	349 (100%)	
	Yes	88 (86%)	14 (14%)	102 (100%)	
**HBsAg+Mother**					0.037
	Unknown	73 (78%)	20 (22%)	93 (100%)	
	No	310 (87%)	45 (13%)	355 (100%)	
	Yes	5 (71%)	2 (29%)	7 (100%)	
**HBsAg+Family**					0.3

member	Unknown	89 (85%)	16 (15%)	105 (100%)	
	No	241 (87%)	37 (13%)	278 (100%)	
	Yes	56 (79%)	15 (21%)	71 (100%)	
Transcutaneous exposure					0.7
	No	342 (87%)	50 (13%)	392 (100%)	
	Yes	12 (86%)	2 (14%)	14 (100%)	
Dope					0.6
	No	375 (85%)	67 (15%)	442 (100%)	
	Yes	7 (100%)	0 (0%)	7 (100%)	
Risky practices					0.8
	No	274 (85%)	47 (15%)	321 (100%)	
	Yes	115 (85%)	21 (15%)	136 (100%)	
Prison					0.6
	No	366 (85%)	63 (15%)	429 (100%)	
	Yes	21 (81%)	5 (19%)	26 (100%)	
Unprotected sex					>0.9
	No	136 (85%)	24 (15%)	160 (100%)	
	Yes	253 (85%)	44 (15%)	297 (100%)	
Sharp object Wounds					0.6
	No	234 (86%)	37 (14%)	271 (100%)	
	Yes	147 (84%)	28 (16%)	175 (100%)	
	Unknown	5 (83%)	1 (17%)	6 (100%)	
Housing style					0.004
	Roommate	38 (72%)	15 (28%)	53 (100%)	
	Family	303 (86%)	50 (14%)	353 (100%)	
	Alone	48 (94%)	3 (5.9%)	51 (100%)	
Housing density					0.5
	1-2 persons	178 (84%)	34 (16%)	212 (100%)	
	>2 persons	211 (86%)	34 (14%)	245 (100%)	
Sharing clothes					0.5
	No	324 (85%)	58 (15%)	382 (100%)	
	Yes	65 (88%)	9 (12%)	74 (100%)	
Sleeping sharing					0.3
	No	149 (84%)	28 (16%)	177 (100%)	
	Yes	239 (86%)	38 (14%)	277 (100%)	
History of hepatitis B					0.005
	No	307 (88%)	43 (12%)	350 (100%)	
	Yes	82 (77%)	25 (23%)	107 (100%)	

Variable	Category	Overall n (%)	HBsAg negative n (%)	HBsAg positive n (%)	p
**Sex**	Female	109 (23,8%)	93(85,3%)	16(14,6%)	>0.9
	Male	348 (76,1%)	296(85,05%)	52(14,9%)	
**Numbers of sexual partners**	more than one	252 (55.1%)	213 (85%)	39 (15%)	0.7
	One	205 (44.8%)	176 (86%)	29 (14%)	
**Knowledge of hepatitis B**	No	209(45.7 %)	179 (86%)	30 (14%)	0.9
	Yes	209(45.7%)	180 (86%)	29 (14%)	
**Transfusion**	No	372(81.4%)	341 (85%)	58 (15%)	0.8
	Yes	44(9.62%)	37 (84%)	7 (16%)	
**History of surgical procedure**	No	435 (95.1%)	367 (84%)	68 (16%)	0.056
	Yes	21(4.5%)	21 (100%)	0 (0%)	

**History of hospital admission**	No	349(76.3%)	297 (85%)	52 (15%)	0.8
	Yes	102(22.3%)	88 (86%)	14 (14%)	
**HBsAg + mother**	No	355(77.6%)	310 (87%)	45 (13%)	0.037
	Yes	7(2.7%)	5 (71%)	2 (29%)	
**Family history of HBV infection**	No	278 (60.8%)	241 (87%)	37 (13%)	0.3
	Yes	71 (15.5%)	56 (79%)	15 (21%)	
**History of Transcutaneous procedure**	No	392(85.7%)	342 (87%)	50 (13%)	0.7
	Yes	14(3.06%)	12 (86%)	2 (14%)	
**History of injection of drugs**	No	442(96.7%)	375 (85%)	67 (15%)	0.6
	Yes	7 (1.5%)	7 (100%)	0 (0%)	
**Risky practices**	No	321(70.2%)	274 (85%)	47 (15%)	0.8
	Yes	136 (29.7%)	115 (85%)	21 (15%)	
**Prison stays**	No	429 (98.8%)	366 (85%)	63 (15%)	0.6
	Yes	22(5.6%)	21 (81%)	5 (19%)	
**Unprotected sex**	No	160(35%)	136 (85%)	24 (15%)	>0.9
	Yes	297(64.9%)	253 (85%)	44 (15%)	
**Sharing of sharp materials**	No	271 (59.2%)	234 (86%)	37 (14%)	0.6
	Yes	175 (38.2%)	147 (84%)	28 (16%)	
**Housing style**	Alone	51 (11.1%)	48 (94%)	3 (5.9%)	0.004
	Roommate	53 (11.5%)	38 (72%)	15 (28%)	
	Family	353 (77.2%)	303 (86%)	50 (14%)	
**Housing density**	1-2 persons	212 (46.3%)	178 (84%)	34 (16%)	0.5
	>2 persons	245 (53.6%)	211 (86%)	34 (14%)	
**Sharing clothes**	No	382(83.5%)	324 (85%)	58 (15%)	0.5
	Yes	74(16.1%)	65 (88%)	9 (12%)	
**Sharing bed**	No	177(38.7%)	149 (84%)	28 (16%)	0.3
	Yes	277(60.6%)	239 (86%)	38 (14%)	
**History of HBV screening**	No	350(76.5%)	307 (88%)	43 (12%)	0.005
	Yes	107(23.4%)	82 (77%)	25 (23%)	

Factors associated with HBV infection among students

Variable with P-value less than 0.3 in the bivariate analysis

Binary logistic regression highlighted the factors associated with HBV carriage, such as age, HBsAg+ mother, history of Hepatitis B tests, level, family members, dope and type of housing. (Table 3).

In multivariate logistic regression, after adjustment of the regression considering other risk factors significantly associated with HBV carriage, the regression showed that only the age, mother's serological status, the mode of residence and the history of hepatitis B remained significantly associated with HBV carriage. Students aged between 26-35 years were 1.11 times less likely to be HBsAg positive (OR= 0.90, 95% CI: 0.42-1.80; p= 0.032). Participants with HBV-uninfected mothers were 2.08 times less likely to be HBsAg positive (OR=0.48, 95% CI: 0.10-4.26; p= 0.027). Students who lived with roommates or family had respectively 5.41 times (OR=5.41, 95% CI: 1.55-25.6; p= 0.025) and 2.69 times (OR=2.69, 95% CI: 0.90-11.7; p= 0.025) risk to be HBsAg carriers than those who lived. Students with no history of Hepatitis B test were 2.17 times less likely to be HBsAg positive (OR= 0.46, 95% CI: 0.25-0.86, p=0.015) (Table 3).

Logistic regression was used to test the association between HBV infection and the independent variables and to identify risk factors. By considering P-value <0.20 in bivariate analysis, HBsAg positive mother, family history of HBV infection, history of transcutaneous procedure, type of housing and history of HBV screening were identified as candidate variables for a multivariate analysis. Having HBsAg positive mother (AOR: 2.60; 95%CI: 1.18-5.80, p=0.018), Having a history of transcutaneous procedure (AOR: 2.63 95%CI: 1.32-5.10, p=0.005), having a flat share (AOR: 7.19, 95%CI 2.08-33.79, p=0.004) and having a history of HBV screening (AOR: 2.03, 95%CI: 1.11-3.65, p=0.019) were significantly associated with HBV status **(Table 3).**

Table 3: Factors associated with HB~~I~~V status of study ~~participants~~participants.

Variables	categories	Univariate regression OR[1]	95% CI[1]	p-value	Multivariate regression OR[1]	95% CI[1]	p-value
Age				0.025			0.032
	< 25	—	—		—	—	
	26-35	0.75	0.37-1.42		0.90	0.42-1.80	
	>35	00			00		
Level				0.14			0.16
	Bachelor 1		—	—		—	—
	Bachelor 2	0.66	0.35-1.22		0.54	0.27-1.06	
	Bachelor 3	0.55	0.29-1.01		0.52	0.26-1.02	
	Master & PhD	0.00			0.00		
Mother HBsAg+				0.075			0.027
	Yes	—	—		—	—	
	Unknown	0.68	0.14- 5.03		1.61	0.23-14.6	
	No	0.36	0.08-2.59		0.48	0.08-3.84	
HBsAg+Family member				0.28	0.28		0,23
	Yes		—	—		—	—
	Unknown	0.67	0.31-1.47		0.42	0.14-1.16	
	No	0.57	0.30-1.14		0.84	0.40, 1.82	
Dope					0.13		
	Yes		—	—		—	—
	No	0.00, NA	2,796-376		0.00, NA	9,892-022	
Housing style				0.005			0.025
	Alone	—	—		—	—	
	Roommate	6.32	1.91-28.7		5.41	1.55-25.6	
	Family	2.64	0.92-11.1		2.69	0.90-11.7	
History of hepatitis B examination				0.007			0.015
	Yes	—	—		—	—	
	No	0.46	0.27-0.80		0.46	0.25-0.86	

Note: Note: 95% CI = 95% Confidence Interval, NA = Not applicable (not retained in the model), OR = Odd Ratio.

Variable	Univariate regression COR (95% CI)	P	Multivariate regression AOR (95% CI)	P
HBsAg positive mother				
No	1		1	
Yes	2.01 (1.13-3.48)	0.015	2.60 (1.18-5.80)	0.018*
Family history of hepatitis B				
No	1		1	
Yes	1.74 (0.88-3.35)	0.102	1.03 (0.47-2.14)	0.936
Transcutaneous procedure				
No	1		1	
Yes	2.62 (1.39-4.81)	0.002	2.63 (1.32-5.10)	0.005*

**Type of housing**				
**Alone**	1		1	
**Family**	2.64 (0.92-11.15)	0.114	2.94 (0.98-12.78)	0.089
**Roommate**	6.32 (1.91-28.71)	0.006	7.19 (2.08-33.79)	0.004*
**History of HBV screening**				
**No**	1		1	
**Yes**	2.18 (1.24-3.75)	0.006	2.03 (1.11-3.65)	0.019*

Note: 95% CI = 95% Confidence Interval, model), COR: crude odds ratio, 1; reference category, *; significantly associated factors p-value <0.05; AOR and adjusted odds ratio.

Discussion

To inform policies aiming to reduce the burden of HBV in Chad and to achieve the objectives of the national health plan 2016-2030, this study was designed to determine the prevalence of HBV and to identify the HBV infection risk factors, among students at the Universities of N'djamena and Emi Koussi. We found, in a population of 457 students from the University of N'Djamena and the University of Emi Koussi, Chad The HBsAg seroprevalence was of HBV infection (HBsAg positivity) of 14.87% (95%CI: 13.9%, 21.7%). This prevalence exceeds the threshold of 8%, and places our study population in the high prevalence group according to the WHO classification (24). Risk factors independently associated with HBV infection were subjects with HBsAg+ mothers, history of transcutaneous procedures, lifestyle, and history of HBV testing. These figures are lower than that reported in studies carried out in the general population of south-west Chad, which were 22.9% (11) and estimate of 19% by the WHO (25). This may be due to the students' younger age. Most HBV infections in high-prevalence (>8%) countries are the result of mother-to-child transmission (MTCT) or early childhood household horizontal transmission (26) (27). However, sexual, and parenteral HBV infection also may occur and other factors, including percutaneous routes of transmission (barbers, piercing, or traditional practices like excision or circumcision), may also be incriminated in HBV transmission. Of sure, students are less exposed than adults and general population. HBV infection is still poorly covered by the national health systems, and it is poorly known among the population, as educational campaigns are rare. The lack of universal health coverage (UHC), in Chad and the lack of a national program for the management of hepatitis B infection is a real obstacle to control the epidemic. According to available data, in Chad, about 60,000 people are covered by health insurance, mainly in the private sector (28). In fact, the Chadian government has recently committed a national program to fight against viral hepatitis, but this has not yet been adequately funded.

Formatted: Normal, Line spacing: 1.5 lines, Don't adjust space between Latin and Asian text, Don't adjust space between Asian text and numbers

Formatted: Font: (Default) Cambria, 12 pt, Not Bold, Font color: Auto

~~,~~ among the included students. As already shown, the HBsAg+ serological status of the mother, the mode of residence and the history of hepatitis B were associated with the carriage of HBV among the students of the universities of N'djamena and Emi Koussi. On the other hand, the presence of family members carrying HBV and the number of sexual partners were not significantly associated with HBV carriage.

~~Regarding the seroprevalence of HBsAg, these results are similar to those of the study conducted in N'djamena which showed a prevalence of 13.5% ([8] and are higher than in the study on patients with acute febrile jaundice in Chad, where the prevalence reached 10.3% [9]. These differences might be related to socio-cultural, demographic, economic and behavioral factors. Most of the students were young, of different origins and cultures, with many sexual partners and various sexual practices. These are factors that may contribute to increase the rate of infection. Other studies conducted elsewhere have reported lower or higher prevalence than in our study.~~ The seroprevalence of HBsAg among students found in this study is higher than that reported in studies from ~~conducted in~~ Togo (4.6%) [17], Cameroon (5.6%) [18] and Ethiopia (11.5%)[19], ~~while~~ but ~~it is lower~~ than that of Nigeria (31.5%) (20]) . While our results are consistent with those reported in Central African Republic (15.5%) (21]. As well as birth dose followed by three doses of pentavalent vaccine as part of a national immunization programme, other prevention measures adapted to students (i.e information about sexuality, sexually transmitted diseases, and risky behavior) are not still implemented in Chad. The student environment may be risky (i.e drug, alcohol, and sex temptation etc.). Therefore, in addition to the extreme poverty of this population, students are tempted due to frequent inappropriate environments. Moreover, in the Chadian context, sexual education in the family remains a taboo that has not yet been broken. This lack of education makes students particularly vulnerable and exposes them to major risks such as unprotected sexual activity.

~~The high seroprevalence in this study implies an improvement in awareness, particularly among young people and students. It implies as well as a generalization of HBV vaccination and particularly catch up vaccination of children at school, before, and screening HBsAg and antiHBc and HBs antibodies of university or high school students before the beginning of the studies.~~

Formatted: Font color: Auto

Formatted: Space After: 8 pt, Adjust space between Latin and Asian text, Adjust space between Asian text and numbers

On the other hand, 23.4% of all students had already been screened for hepatitis B and the seroprevalence of HBsAg was 23% among these students. However, the seroprevalence of HBsAg was 12% among students who had not been previously tested. This result corroborates the WHO study which highlighted that more than 124,000 Africans die each year from the consequences of undetected and untreated hepatitis [3]. This finding could be explained by the low effectiveness of awareness-raising activities. Many people in Africa are screened following a medical consultation. However, in Chad, this screening is not systematic and not required by clinicians [6]. Therefore, asymptomatic patients remain a potential source of HBV infection.

Our results are similar to other studies that have reported no association between HBV infection and risk factors such as history of surgery, acupuncture and blood transfusion, smoking and alcohol consumption [18,21,22] . These results could be explained by the low proportion of participants with this background risks in the current study. Students aged between 25-35 years of age were less likely to contract HBV. A study conducted among Senegalese military personnel showed that participants aged 27-37 years were less likely to contract HBV (OR=0,16 95% CI =0,04-0,68; p=0,012)[23]. This result could be explained by clearance of HBsAg leading to spontaneous healing of hepatitis B or occult hepatitis.

Students with HBsAg-negative mothers were statistically unlikely to acquire HBV infection. We also found a significant association between HBV infection in the students and the HBs Ag + mother. The association between HBV infection and the HBV status of mother is coherent with results reported elsewhere . These results support findings from the literature reporting that sub-Saharan Africa is a highly endemic area where perinatal infection is common[24]. The same findings were observed in Gambia in 2007, where mother-to-child transmission was responsible for 16% of chronic infections [25] and in Cameroon, where children born from HBsAg positive mothers had a substantial risk of mother-to-child transmission even despite birth dose vaccination [26]. In 2019, the vaccination program of the Ministry of Health reported that less than 50% of children are fully vaccinated. This situation allows to say that the transmission of hepatitis B from mother to child are factors spreading the infection. To date, there is no program in place to prevent the transmission of HBV from mother to child In Chad i.e., birth dose HBV vaccination of newborns.

~~These results highlight the importance of a policy aimed at preventing mother-to-child transmission of HBV.~~

In this~~Our~~ study ~~also found that students who lived in shared~~ participants living in roommate accommodation were seven times more likely to acquire ~~are more likely to contract~~ HBV infection. This ~~suggests a decent home in addition to a protective factor against HBV infection. Therefore, our findings suggest that authorities could help students to improve student's life conditions.~~underlines the importance of changing room accommodations habits and built new housing for students in N'Djamena, as universities in Chad do not have student accommodation. The roommate is an economic solution as it allows sharing daily expenses besides renting. Despite its abundant natural resources, Chad remains one of the poorest countries in the world (36) ; with most of its inhabitants living in poverty, with a national incidence of 46.7% [32]. HBV can survive on environmental surfaces for more than a week. Indirect exposure to the virus can occur through contaminated objects (38). The risk of sharing objects for personal use, e.g., hairbrushes, combs, razors, can cause damage to the skin or mucous membranes and transmit HBV. This type of horizontal transmission is most common in areas of high endemicity, where hygiene is poor(39).

Students who had a history of transcutaneous medical examinations were almost three times more likely to acquire HBV infection than their counterparts [AOR = 2.63, 95% CI = 1.32-5.10, p = 0.005]. In fact, several factors determine the risk of HBV nosocomial transmission. These include invasive surgical procedures [35], failure to follow aseptic techniques and recommended control practices to prevent postoperative infections due to cross-contamination of medical equipment and devices. HBV is the most highly transmissible of the blood-borne viruses that are important in the health care setting. Available data suggest that the risk of transmitting HBV from infected healthcare workers to patients is much higher than for other viruses such as hepatitis C or HIV (41).

The study also showed ~~found~~ that the likelihood of being an HBV carrier was twice among students who had history of HBV screening. Our findings are consistent with data reported by Halatoko and al in Togo, who found a statistical association between knowledge of one's HBV status and hepatitis B infection (42).

Formatted: Normal, Space After: 0 pt, Line spacing: Double, Pattern: Clear

Formatted: Space After: 8 pt, Line spacing: 1.5 lines

Formatted: Font: (Default) Cambria, 12 pt

The participants in the study was voluntary ~~who knew their HBV serological status were twice as likely to be HBV carriers. Indeed, this result could illustrate~~ this may be a limitation of the study. ~~so far as participation in the study was voluntary. Thus, S~~students who know they are already infected ~~could~~ might be tempted to participate in the study hoping to receive treatment or to check ~~benefit from the study in terms of clinical management and care or hoping to verify~~ their serological status.There is a need to focus on management and clinical follow-up for student who were tested positive for HBsAg, and to encourage those who were tested negative to complete the vaccination course. In the current study, all participants were sensitized about HBV and those tested positive were referred to health facilities for follow up.

Formatted: Space After: 0 pt, Line spacing: Double

Formatted: Font: (Default) Calibri, 11 pt, Pattern: Clear

Strengths and limitations of the study

This is a multicenter study bringing together a mixed ~~diverse~~ population of students from the universities of N'djamena and Emi-Koussi. This reflects the epidemiology of HBV within this community. The study did not include ~~an~~ assay for the detection of total anti-HBc antibodies (Ab) and anti-HBc IgM Ab~~anti-HBc and HBeAg IgM markers~~. This could have made it possible to distinguish between acutely infected subjects(with anti-HBc IgM Ab), ~~and~~ chronic carriers and subjects who have recovered from acute or chronic infection (anti-HBc Ab positive).. ~~Quantification of HBV DNA would also have allowed the identification of cases of occult hepatitis.~~

Conclusions

HBV is ~~one of the~~ an infectious ~~neglected tropical~~ disease~~s~~ of great ~~major~~ concern in Chad and health authorities plan to eliminate it by 2030. The prevalence of HBV found in our study confirm that Chad is a highly endemic country. Having a HBsAg+ mother, living in shared flats, having a history of transcutaneous procedure and of HBV screening were factors independently associated with HBV infection among students. Policies are needed to reduce the incidence and mortality of VHB disease.

~~This study conducted in the universities setting revealed a prevalence of 14.87%. HBsAg+ mother and roommate status were risk factors associated with HBsAg carriage. These results suggest the need for improved awareness, screening and increasing vaccination coverage among young people and particularly among women. This study can also serve as a basis for other subsequent medical and scientific studies on neglected tropical diseases in young people.~~

Our results highlight the need to reinforce prevention, screening, management, and treatment measures, such as MTCT prevention through pregnant women HBV, birth dose vaccination of newborns, vaccination part of EPI or catch-up vaccination of children or students and screening the general population. Moreover, as drugs active against HBV, like nucleotide analogs, are now available at low cost, subjects with signs of liver disease should be treated according to WHO recommendations.

Recommendations

In view of our results, a policy to fight against this disease should include the sensitization of at-risk population such as students on the mode of transmission of HBV, introducing hepatitis B vaccination at birth, making full hepatitis vaccination status mandatory before a child starts school, promoting mass screening to identify and treat chronic HBV carriers to reduce transmission in the community, reducing the vaccination cost. The policy should promote a free of charge of HBV screening for pregnant women and the improving of the living conditions the population through decent accommodation including those of students.

Formatted: Space After: 8 pt, Line spacing: 1.5 lines

Formatted: Font: (Default) Cambria, 12 pt

List of abbreviations

Anti-HBs: Hepatitis B surface antibody; AOR: Adjusted odd ratio; CI: Confidence interval; COR: Crude odd ratio; EPI: Expanded Program of Immunization; HBsAg: Hepatitis B surface antigen; HBV: Hepatitis B virus; HCC: Hepatocellular carcinoma; WHO: World Health Organization.

Acknowledgements

We are thankful to laboratory technicians of Hôpital de la Paix in N'djamena who have performed the blood sampling and tests. We express our gratitude to data collectors and supervisors of the survey.

We thank the deans of Cheikh Anta Diop University for their guidance. We are thankful to the secretaries of the students who have facilitated the sensitization of students to participate to the study and the students who participated in the study.

Availability of data and materials

All relevant data are within the paper and supporting information files.

Conflicts of interest

The authors have declared that no competing interests exist.

Funding

The author(s) received no specific funding for this work.

Author Contributions

Conceptualization: Nalda Debsikréo, Françoise Lunel-Fabiani.

Data curation: Nalda Debsikréo, Birwé Léon Mankréo, Azoukalné Moukénet.

Formal analysis: Nalda Debsikréo, Birwé Léon Mankréo, Azoukalné Moukénet.

Funding acquisition: Nalda Debsikréo.

Investigation: Nalda Debsikréo, Merwa Ouangkake, Nathan Mara, Ali Mahamat Moussa.

Methodology: Nalda Debsikréo, Birwé Léon Mankréo, Azoukalné Moukénet,Ali Mahamat Moussa Ndèye Coumba Toure-Kane, Françoise Lunel-Fabiani.

Project administration: Nalda Debsikréo, Merwa Ouangkake, Nathan Mara, Ali Mahamat Moussa.

Software: Nalda Debsikréo, Birwé Léon Mankréo, Azoukalné Moukénet.

Laboratory: Nalda Debsikréo, Merwa Ouangkake, Nathan Mara.

Validation: Nalda Debsikréo ;Birwé Léon Mankréo, Azoukalné Moukénet, Merwa Ouangkake, Nathan Mara, Ali Mahamat Moussa, Ndèye Coumba Toure-Kane, Françoise Lunel-Fabiani.

Visualization: Nalda Debsikréo, Birwé Léon Mankré, Azoukalné Moukénet.

Writing – original draft: Nalda Debsikréo

Writing – review & editing: Nalda Debsikréo, Birwé Léon Mankré, Azoukalné Moukénet, Merwa Ouangkake, Nathan Mara, Ali Mahamat Moussa, Ndèye Coumba Toure-Kane, Françoise Lunel-Fabiani.

References

1. WHO. World Hepatitis Day 2020 [Internet]. [cité 22 juin 2022]. Disponible sur: https://www.who.int/campaigns/world-hepatitis-day/2020

2. Schweitzer A, Horn J, Mikolajczyk RT, Krause G, Ott JJ. Estimations of worldwide prevalence of chronic hepatitis B virus infection: a systematic review of data published between 1965 and 2013. Lancet Lond Engl. 17 oct 2015; 386(10003):1546‑55.

3. WHO. World Hepatitis Day, 28 July 2021 [Internet]. WHO | Regional Office for Africa. [cité 22 juin 2022]. Disponible sur: https://www.afro.who.int/regional-director/speeches-messages/world-hepatitis-day-28-july-2021

4. Koffas A, Kumar M, Gill US, Jindal A, Kennedy PTF, Sarin SK. Chronic hepatitis B: the demise of the 'inactive carrier' phase. Hepatol Int. 1 avr 2021; 15(2):290‑300.

5. Ferreira MS. Diagnosis and treatment of hepatitis B. Rev Soc Bras Med Trop. août 2000; 33:389‑400.

6. Tcd_rpt_annuairesstatistiquessanitaires_ministeresante_2017.pdf [Internet]. [cité 13 mai 2022]. Disponible sur: https://www.humanitarianresponse.info/sites/www.humanitarianresponse.info/files/documents/files/tcd_rpt_annuairesstatistiquessanitaires_ministeresante_2017.pdf

7. Suesstrunk J, Djongali FB. Hepatitis B virus prevalence in rural areas in south-west Chad. Trop Doct. oct 2017;47(4):374‑7.

8. Bessimbaye N, Moussa AM, Mbanga D, Tidjani A, Mahamat SO, Ngawara MN, et al. Séroprévalence de l'Ag HBs et de l'anticorps Anti VHC chez les personnes infectées par le VIH1 à N'Djamena, Tchad. Bull Société Pathol Exot. 1 déc 2014; 107(5):327‑31.

9. Yandai FH, Traore KA, Moussa AM, Ouoba BL, Ouoba JB, Bolti MA, et al. Prevalence and Diversity of Hepatitis Virus Markers among Patients with Acute Febrile Jaundice in Chad. Microbiol Res. déc 2021; 12(4):878‑87.

10. Zampino R, Boemio A, Sagnelli C, Alessio L, Adinolfi LE, Sagnelli E, et al. Hepatitis B virus burden in developing countries. World J Gastroenterol. 14 nov 2015; 21(42):11941‑53.

11. Tharmaphornpilas P, Rasdjarmrearnsook A orn, Plianpanich S, Sa-nguanmoo P, Poovorawan Y. Increased risk of developing chronic HBV infection in infants born to chronically HBV infected mothers as a result of delayed second dose of hepatitis B vaccination. Vaccine. 19 oct 2009; 27(44):6110‑5.

12. Mast EE, Alter MJ, Margolis HS. Strategies to prevent and control hepatitis B and C virus infections: a global perspective. Vaccine. 26 mars 1999; 17(13‑14):1730‑3.

13. Gomes C, Wong RJ, Gish RG. Global Perspective on Hepatitis B Virus Infections in the Era of Effective Vaccines. Clin Liver Dis. août 2019; 23(3):383‑99.

14. Tchad-Politique-Nationale-de-Sante-2016-2030.pdf [Internet]. [cité 13 mai 2022]. Disponible sur: https://www.prb.org/wp-content/uploads/2020/06/Tchad-Politique-Nationale-de-Sante-2016-2030.pdf

15. La formation à l'université de N'Djaména.Disponible sur: https://archive.wikiwix.com/cache/index2.php?url=https%3A%2F%2Fwww.universite-ndjamena.td%2Fformation#federation=archive.wikiwix.com

16. Charan J, Biswas T. How to Calculate Sample Size for Different Study Designs in Medical Research? Indian J Psychol Med. 2013; 35(2):121‑6.

17. Ekouevi DK, Thomas A, Sewu D, Lawson-Ananissoh L, Tchounga B, Salou M, et al. Prevalence of Hepatitis B among Students from the University of Lomé, Togo in 2015. Open J Epidemiol. 8 août 2017; 7(3):262‑72.

18. servais albert fiacre E. Statut Vaccinal Contre le Virus de l'Hépatite Virale B et portage de l'Antigène Hbs chez les Etudiants en Médecine et en Pharmacie de l'Université de Douala au Cameroun. Health Sci Dis. 1 janv 2014;

19. Tesfa T, Hawulte B, Tolera A, Abate D. Hepatitis B virus infection and associated risk factors among medical students in eastern Ethiopia. PLOS ONE. 19 févr 2021; 16(2):e0247267.

20. Tula MY, Iyoha O. A Cross-sectional Study on the Sero-prevalence of Hepatitis B Surface Antigen (HBsAg) among Apparently Healthy Students of a Tertiary Institution in North-Eastern Nigeria. Int J Trop Dis Health. 10 janv 2015; 7:102‑8.

21. Komas NP, Baï-Sepou S, Manirakiza A, Léal J, Béré A, Le Faou A. The prevalence of hepatitis B virus markers in a cohort of students in Bangui, Central African Republic. BMC Infect Dis. 29 juill 2010; 10:226.

22. Mutocheluh M, Owusu M, Kwofie TB, Akadigo T, Appau E, Narkwa PW. Risk factors associated with hepatitis B exposure and the reliability of five rapid kits commonly used for screening blood donors in Ghana. BMC Res Notes. 4 déc 2014; 7(1):873.

23. Diop M, Diouf A, Seck SM, Lo G, Ka D, Massaly A, et al. [Prevalence of hepatitis B surface antigen and its associated factors in Senegalese military personnel sent on mission to Darfur]. Pan Afr Med J. 2017; 26:154.

24. Shimakawa Y, Toure-Kane C, Mendy M, Thursz M, Lemoine M. Mother-to-child transmission of hepatitis B in sub-Saharan Africa. Lancet Infect Dis. 1 janv 2016; 16(1):19‑20.

25. Shimakawa Y, Lemoine M, Njai HF, Bottomley C, Ndow G, Goldin RD, et al. Natural history of chronic HBV infection in West Africa: a longitudinal population-based study from The Gambia. Gut. 1 déc 2016; 65(12):2007‑16.

26. Shimakawa Y, Veillon P, Birguel J, Pivert A, Sauvage V, Guillou-Guillemette HL, et al. Residual risk of mother-to-child transmission of hepatitis B virus infection despite timely birth-dose vaccination in Cameroon (ANRS 12303): a single-centre, longitudinal observational study. Lancet Glob Health. 1 avr 2022; 10(4):e521‑9.

Supporting information

S1 File. Questionnaire (docx)

S2 File. Checklist (docx)

S3 File. Data_base R (XLS)

S4 File code R

Formatted: English (United States)

Dear Author,

I am wishing to acknowledge author's work entitled: 'Prevalence of hepatitis B virus infection and associated risk factors among students in N'Djamena, Chad'. The results illustrated the prevalence of hepatitis B virus among students. Therefore, this study will be useful for future prevention and control of hepatitis infection through the knowledge of prevalence HBV and risk factors among students. The manuscript is more or less in line with the vision, objectives and instructions of PLoS One. Despite its potential, however, this manuscript contains major errors in data presentation and interpretation, grammatical errors which need to be corrected (see the attached file).

General Comments

1. Although what authors are reporting is not a novelty, they must however be commended for their efforts in reporting what is prevalence of HBV among students in their locality. But there are so many researches worldwide which were done by more advanced lab techniques like ELISA and molecular techniques (PCR).

Our response:

We thank reviewer for comments, we carried out additional tests by ARCHITECT HBsAg Quantitative confirming the results. Line:158-161

2. Use of English language is poor in certain sections and would require a detailed revision.

Our response:

We thank reviewer 1 for comments.

We have answered point by point and correct typo or grammatical errors.

3. Based on PLoS One authors' guideline, figures must be uploaded separately in tif or other form.

Our response:

"Thanks for reminding us about the respect of style requirement of PLOS One related to figures. However, we have not a figure to integrate into this paper".

Title

- The title was good. But why you removed the area name 'Emi Koussi' from the title? You talked more about this study area in the methods part.

Our response:

Thank you, "Emi Koussi" is the name of one of Universities whereas the study area itself is N'djamena

Abstract

- The punctuation colon (:) in each subheading was not necessary.

  Thank you for the remark. We have removed it accordingly

- Line 17; read as 'Viral hepatitis B (HBV) is a major public health threat'. It is better written as 'Viral hepatitis B virus (HBV) is a major public health problem'. Here the word 'threat' is not the appropriate word.

  Thank you for mentioning this. We have modified accordingly in -- line 21; "Hepatitis B virus (HBV) is a major public health problem." Line 20.

- Line 20-21; 'This study aims to determine the prevalence-------------' It is better written as, 'This study aimed to determine the prevalence--------------------'

  Thanks, in line 23-24 we have replaced 'This study aims to determine the prevalence -------------' by 'This study aimed to determine the prevalence---------------'

- Line 23; '------ conducted in July 2021 including 457 students randomly selected--------'. Here a specific length of time should be mentioned. Similarly, which random sampling technique was used? Simple or systematic?

- **Our response:**
- Thanks for the comment. Line 28; '-------- conducted in July 2021 including 457 students randomly selected-------------'. Retain " ....from 3 to 23 July 2021. Simple random sampling technique was used to calculate sample size. Participant were not selected randomly but based on a consecutive sampling.
- Which specimen type you have processed? It should be described before you talk about the method.
- The type of sample taken is serum. Line: 29.
- Line 26; 'Descriptive analyzes and binary logistic regressions were used to examine the relationship between dependent variables and sociodemographic including other risk factors'. It is better you say, 'Descriptive analysis and binary logistic regressions were used to examine the relationship between dependent variables and socio-demographic factors including other risk factors.'

Thank you for mentioning this. We replaced the sentence as suggested: line 166-167; 'Descriptive analysis and binary logistic regressions were used to examine the relationship between dependent variables and socio-demographic factors including other risk factors.'

- Among how many study participants the study conducted?
- **Thank you,** the study was conducted among 457 participants. University of N'djamena counts 26349 students.
- In the result part, it is better you include both the numerator and denominator together with percentage in presenting prevalence, for example A/B (14.87%).

Thank you for this comment, but we do not agree with this comment because the tables will be too clumpy.

- Too short and shallow result presentation

Thank you, the result presentation has been revised and we hope that it is more detailed and requested. Line: 208-262.

- Your result completely contrasts the fact. Are you interpreting the result inversely? See the data, 'Students aged between 26-35 years (0.032), who were born from HBsAg positive mothers (0.027), who lived with a roommate (0.025) or had a history of hepatitis (p=0.015) were less likely to contract HBV infection'. For example, A participant who lived with a roommate has a probability of acquiring HBV 4.5 times than those living lonely (OR= 5.42, 95% CI: 1.55-25.6; p= 0.025). The author better go through the analysis and correct mistakes that appeared.

Thank you, we have reviewed the analysis: "Having mother HBV (AOR: 2.60; 95%CI: 1.18-5.80, p=0.018), having transcutaneous procedure (AOR: 2.63 95%CI: 1.32-5.10, p=0.005), being flat share (AOR: 7.19, 95%CI 2.08-33.79, p=0.004) and having history of HBV screening (AOR: 2.03, 95%CI: 1.11-3.65, p=0.019) were associated with a higher risk of HBV infection" Line: 249-253.

- What is your baseline to say, 'high prevalence of HBV infection'?

Thank you for this remark. by the way, we would rather say that this prevalence, higher than 8%, places our study population in the high endemicity group according to the WHO classification. We have changed the sentence in the manuscript. Line: 272-273.

- Which type of conclusion is it? 'This study highlights some solutions to improve the situation of hepatitis B in Chad and suggests policies to eliminate viral hepatitis'.

Thank you, we have revised the presentation of the conclusion. Line: 367-378

Additional key words: -HBsAg, risk factor

We have added Additional key words: -HBsAg, risk factor. Line 52.

- It should be re-written and make adjustments based on the comments in the methods and results part that follows.

- Thanks for your comments, we have revised the whole manuscript and focus on highlighted parts accordingly.

Introduction

- The introduction seems like literature review, so it is better modified.

Thanks for your comments. We modified the introduction. Line: 54-107.

- The word treat is not the appropriate word; please replace by other word.

Thank you, we have changed the sentence according to your suggestion.

- The introduction described the burden among clinically ill participants, not apparently healthy individuals (e.g. students). Therefore, the author better show the burden of HBV in similar or related study populations.

  Thank you, we have changed the introduction according to your suggestion, by changing some references.

-

- There was no description about the microbiology of HBV.

  Thank you, we have added a paragraph about the microbiology of HBV according to your suggestion. Line 55-58.

- I do not think the importance of writing values for P-value, 95%CI and OR. For example, 'Infants infected during the perinatal period 62 have 3.74 times (OR=3.74, 95% CI: 0.97–14.39) risk of becoming chronic carriers [11]'.

Thank you, we have taken this remark into account.

- Line 82-84 should be deleted. 'Identifying these risk factors for HBsAg 83 carriage with appropriate management would reduce the disease burden. Knowledge of the HBV 84 infection risk factors would also guide policies aimed at reducing the HBV burden'.

  Thank you, we have deleted this sentence.

Materials and Methods

- Line 93; the subheading should be 'Sample size determination and sampling technique'.

  Thank you, we have considered the title 'Sample size determination and sampling technique': line 119.

- About the sampling technique, please see the comment in the abstract.

- The 'selection of variables' subheading better be replaced by 'Socio-demographic data collection'

  Thank you, we have considered the title 'Socio-demographic data collection': line 143.

- The 'Biological analysis' also should be replaced by 'Laboratory data processing'

  Thank you, we have considered the title 'Laboratory data processing': line 153.

- In the ethical clearance part, were all participants above the age of 18 years old? If not, why did not you take assent from participants below the age of 18 years old?

Thank you, all participants were above the age of 18 years old. Age ranged from 19 to 59 years. Line: 209.

- Your participants were apparently healthy individuals (students). There should be a mechanism in place to link those who need treatment with the healthcare system. How do you manage the psychological and potential social harm on HBV positives that do not need treatment? So ethical issues were not well described.

  Thank you for raising this point, HBV-positive people have been referred to the focal point of the fight against hepatitis in Chad for follow-up and advice. Line: 197-201.

- Specimen collection and processing needs to be well written.

  Thank you, you pointed out this commend. We proceeded to rewrite the specimen collection. Line 144-152.

Results

- Your subheading 'Sample characteristics' was not the appropriate one. It is better you replace by, ' Socio-demographic characteristics'
- Thank you, we have considered the title 'Socio-demographic characteristics': line 207.
- Based on your sample size determination your sample size was 422. Why you included 457 in the result part?

422 is the minimum size of the calculated sample. The sample was distributed proportionally to faculties selected purposely based on the number of students with Faculty of Languages, Letters, Arts and Communication (75 students), Faculty of Educational Sciences (80 students), Faculty of Exact Sciences and Applied (50 students), Faculty of Economics and Management (88 students), Faculty of Human and Social Sciences (75 students) and University of Emi Koussi (50 students). A total of 457 were students included in the study: line 127-134.

- You mix up the socio-demographic characteristics of participants with prevalence of HBV as well as risk factors. I recommend you to present, first the socio-demographic characteristics and then prevalence of HBV, finally risk factors for HBV.

  Thank you for this recommendation, we have presented, first the socio-demographic characteristics (table 1) line 219-220, and then prevalence of HBV (table 2) line 255-256, finally risk factors for HBV(table 3) line 263-264.

- What is your base to classify the age group? Have you associated with possible risks of interest? It is better again re-group and perform the analysis.

Thank you, we have reclassified the Age category: <20, 20 – 30, 30- 40 and > 40, referring to the article "bdoul Rahamane Njigou Mawouma, Amadou Hapsatou Djoulatou, Eliane Ornella Komnang, Etienne Omolomo Kimessoukie. Facteurs associés à l´infection de l´hépatite B chez les femmes enceintes dans les formations sanitaires du district de santé de Mokolo/Région de l´Extrême-Nord Cameroun. **PAMJ. 21 Jan 2022. 41(61)"**

- Is there any difference between the two subheadings? 'Factors associated with HBV infection among students' and 'Prevalence and risk factors associated with HBsAg carriage'. For me it was not clear.

Thanks, the two subtitles have been rewritten: - Estimated prevalence of HBsAg: line 255-256 and Factors associated with HBV infection among students. line: 263-264.

- It was difficult to understand/catch up the concepts of the tables. They lacked OR and 95%CI. Yet you presented in the text. From where you get it (OR and 95%CI)?

Thank you, we improved the reading of the tables and bring out the odds ratios and their 95% confidence intervals. Line: 263-264.

- Similarly, it is better you represent the crude odds ratio by COR and the adjusted odds ratio by AOR than simply by OR.

Thank you, we have taken into consideration your suggestion, we have represented the crude odds ratio by COR and the adjusted odds ratio by AOR. Line: 263-264.

- All table titles should be complete in description, area and period of study.

Thank you for this advice, we have taken them into account: Table 1: Socio-demographic characteristics of study participants (line 219-220), Table 1: Estimated prevalence of HBsAg (line255-256) and Table 3: Factors associated with HBV status of study participants (line263-264).

Discussions

- Since the necessity of doing this work was already described in the introduction part. So here writing about it was not necessary. Therefore, delete the following. Line 206 'To inform policies aiming to reduce the burden of HBV in Chad and to achieve the objectives of the 207 national health plan 2016-2030, this study was designed to determine the prevalence of HBV and to 208 identify the HBV infection risk factors, among students at the Universities of N'djamena and Emi Koussi'.

  Thank you, these sentences have been deleted.

- Authors tried to compare their findings with different reports from worldwide. They also better justify the reason for variation among results of different research findings with respect to theirs' based on actual situation; most of the reason for variation mentioned in the discussion part were already intensively described by other authors.

- Thank you, we have improved the
- discussion

  Most of the literatures used for comparisons were researches on patients. In this case these literatures were incomparable to this study.

  Thank you, we rewrote the discussion: line 269-365.

Conclusions

- Normally, conclusion emanates from your findings of the research.

Thank you for the remark, the conclusion has been rewritten. Line: 366-378.

- Revise it and accommodate recommendation of community/government importance.

Thank you for the remark, the recommendation has been rewritten. Line: 379-387.
