# Supplementary material for: Prevalence of hepatitis B virus infection and its associated factors among students in N’Djamena, Chad
Source: PLoS One. 2024 Apr 18;19(4):e0273589. doi: 10.1371/journal.pone.0273589 (PMC11025733; doi:10.1371/journal.pone.0273589)
Supplement: Attachment — Submitted filename: PONE-D-22-22361_R5_Annotated.pdf [file pone.0273589.s014.pdf]

PLOS ONE

Titre : Prévalence de l'infection par le virus de l'hépatite B et ses facteurs associés chez les étudiants de N'Djamena, Tchad.

--Manuscript Draft--

**Manuscript Number:**	PONE-D-22-22361R5
**Article Type:**	Research Article
**Full Title:**	Titre : Prévalence de l'infection par le virus de l'hépatite B et ses facteurs associés chez les étudiants de N'Djamena, Tchad.
**Short Title:**	Hepatitis B virus and associated factors among Chadian students
**Corresponding Author:**	NALDA DEBSIKREO<br>Cheikh Anta Diop University of Dakar: Universite Cheikh Anta Diop de Dakar<br>Dakar, SENEGAL
**Keywords:**	KEY WORDS:hepatitis B, HBsAg, prevalence, associated factors, students, Chad.
**Abstract:**	Introduction<br><br>Infection by hepatitis B virus (HBV) is a major issue in public health. The prevalence of HBV in Chad is 12.4%, all age groups considered. Here, we aimed to determine the prevalence of HBV and its associated factors among university students in N'Djamena, the country's capital.<br><br>Methods<br><br>A cross-sectional survey of students at either the University of N'djamena or Emi Koussi University was conducted from 3 to 23 July 2021. All participating students provided signed, informed consent and were included in the study consecutively. Blood samples were collected, and serum tested for hepatitis B surface antigen (HBsAg) using the Determine HBsAg rapid test kit, with confirmation of positive tests on an Abbott Architect i1000SR analyzer. Descriptive analysis and logistic regression were used to determine associations between the outcome variable and independent/covariate variables.<br><br>Results<br><br>A total of 457 students with a median age of 24 years were included across different faculties. The prevalence of HBV infection was 14.87% (68/457). Most students (75%) were aged 25 years or less. Unprotected sex was reported by 64.9% of the students and multiple sexual partners by 53.6%. Furthermore, 45.7% of them reported having no knowledge of hepatitis B. Having an HBsAg-positive mother (AOR: 2.11), having a history of transcutaneous medical procedures (AOR: 2.97) and living with a family (AOR: 4.63) were significantly associated with HBV status. Age ≥26 years appeared as a protective factor (AOR = 0.41).Conclusion<br><br>Our study detected a high, 14.87% prevalence of HBV infection among students in N'djamena, Chad, and shed light on its associated factors. HBV prevention strategies should include raising awareness among students, making full hepatitis vaccination mandatory before children begin school, promoting mass screening to identify & treat chronic HBV carriers and reduce transmission, and reducing the cost of vaccination.
**Order of Authors:**	NALDA DEBSIKREO
	Birwé Léon Mankréo
	Azoukalné Moukénet
	Merwa Ouangkake
	Nathan Mara
	Ali Mahamat Moussa

Powered by Editorial Manager® and ProduXion Manager® from Aries Systems Corporation

	Ndèye Coumba Toure-Kane
	Françoise Lunel-Fabiani
**Opposed Reviewers:**	
**Response to Reviewers:**	Response to reviewers<br>One of the reviewers suggested that final version should incorporate the following changes based on the manuscript evaluation:<br>"In the submitted manuscript “Prevalence of hepatitis B virus infection and its associated factors among students in N’Djamena, Chad”, PONE-D-22-22361R2, this study addressed the high prevalence of HBV infection among university students in Chad. HBV prevention, especially HBV immunization, should be considered in low-income countries.<br>However, there are some points that need to be clarified:<br>1.The authors should mention inclusion criteria in the method part (e.g., sex, age, health status, etc.).<br><br>Thank you for this observation. Students who accepted to participate in the study were enrolled consecutively irrespective of age, sex and other sociodemographic characteristics or risk factors line 105-109<br>2.The data was collected using a questionnaire, was it a self-administered questionnaire or face-to-face interview? Please specify in data collection method.<br>Thank you for your pertinent observation. The data was it questionnaire face-to-face line 122-123<br>3. The prevalence of HBV infection was 14.87%, what’s about 95%CI? Please specify.<br>14.87 is the prevalence of HBsAg in the population studied (95% CI = 13.9-21.7%). Line 193<br>4. I would suggest using median age instead of mean age.<br><br>Thank you for this suggestion. Median age was 24 years. Line 185.<br>5. Considering p<0.30 in bivariate analysis, why did the authors select only five variables for multivariable logistic regression? (What about age, marital status, knowledge of HBV, history of drug injection, density of residence? there were also p<0.30.)<br><br>Age was included in the analysis (age>26 was a protective factor). A previous reviewer and the editor suggested, with reason, to remove knowledge of HBV, which of course is a confounding factor. We have forgotten to remove it from the manuscript in the data analysis paragraph. Marital status, history of drug injection and density of residence were also included in the analysis, but were not significantly associated with HBs Ag positivity (see table 3). Line 215<br>6. The authors did not ask about the history of HB immunization in the study participants. Although most likely did not benefit from vaccination against HBV in childhood as part of EPI, some may receive the vaccine in adult. We therefore can distinguish between who has protective or susceptible for HBV infection."<br><br>Thank you for your comment. Unfortunately, students were not asked about their vaccination status. Please see discussion — line 275—277<br><br>I commend the authors did a great job by incorporating reviewers' comments which substantively improved the paper. However, the paper doesn't until now rigorously align with Plos One's authors guidelines. Please note the following:<br>1. References: Plos One uses brackets;<br>Thank you for this observation, we've changed hooks<br>2. In all Tables, add 1 decimal for percentages<br>Thank you for your comment, we have added 1 decimal for percentages. Table 1: line 188-189, Table 2:203-204 Table 3:214-215<br>3. When citing R, the date is unnecessary<br>Thank you, we have deleted. Line 171<br>Additionally, reviewer #2 has raised some comments to be integrated. I suggest to put these questions in the discussion section or alternatively, add a study limitations section.<br>Thank you for this suggestion, we have added a section on the Strengths and limitations

	I commend the authors did a great job by incorporating reviewers' comments which substantively improved the paper. However, the paper doesn't until now rigorously align with Plos One's authors guidelines. Please note the following:<br>1. References: Plos One uses brackets;<br>Thank you for this observation, we've changed hooks<br>2. In all Tables, add 1 decimal for percentages<br>Thank you for your comment, we have added 1 decimal for percentages. Table 1: line 188-189, Table 2:203-204 Table 3:214-215<br>3. When citing R, the date is unnecessary<br>Thank you, we have deleted. Line 171<br>Additionally, reviewer #2 has raised some comments to be integrated. I suggest to put these questions in the discussion section or alternatively, add a study limitations section.<br>Thank you for this suggestion, we have added a section on the Strengths and limitations<br>However, there are some points that need to be clarified:<br><br>1. the authors should mention inclusion criteria in the method part (e.g., sex, age, health status, etc<br>Thank you for this observation. We have revised the text accordingly, stating that students who accepted to participate in the study were enrolled consecutively irrespective of age, sex, other sociodemographic characteristics or risk factors. Line 105–109.<br>2. The data was collected using a questionnaire, was it a self-administered questionnaire or face-to-face interview? Please specify in data collection method.<br><br>Thank you for your pertinent observation. The interviews were done during face-to-face meetings. Line 122–123.<br>3. The prevalence of HBV infection was 14.87%, what's about 95%CI? Please specify.<br>14.87 is the prevalence of HBsAg in the population studied (95% CI = 13.9–21.7%). This confidence interval was added to the text. Line 195.<br>4. I would suggest using median age instead of mean age.<br>Thank you for this suggestion. Median age was 24 years. Line 185.<br><br>5. Considering p<0.30 in bivariate analysis, why did the authors select only five variables for multivariable logistic regression? (What about age, marital status, knowledge of HBV, history of drug injection, density of residence? there were also p<0.30.)<br>Age was included in the analysis (age ≥26 was a protective factor). A previous reviewer and the editor suggested, with reason, to remove knowledge of HBV, which of course is a confounding factor. We had forgotten to remove it from the manuscript in the data analysis paragraph. Marital status, history of drug injection and density of residence were also included in the analysis, but were not significantly associated with HBs Ag positivity (see Table 3).<br><br>6. The authors did not ask about the history of HB immunization in the study participants. Although most likely did not benefit from vaccination against HBV in childhood as part of EPI, some may receive the vaccine in adult. We therefore can distinguish between who has protective or susceptible for HBV infection.<br>Thank you for your comment. Unfortunately, students were not asked about their vaccination status. We've touched upon this point in the discussion. Line 274–275
**Additional Information:**	
**Question**	**Response**
**Financial Disclosure**<br><br>Enter a financial disclosure statement that describes the sources of funding for the work included in this submission. Review the submission guidelines for detailed requirements. View published research articles from *PLOS ONE* for specific	The author(s) received no specific funding for this work

examples.<br><br>This statement is required for submission and **will appear in the published article** if the submission is accepted. Please make sure it is accurate.<br><br>**Unfunded studies**<br>Enter: *The author(s) received no specific funding for this work.*<br><br>**Funded studies**<br>Enter a statement with the following details:<br>• Initials of the authors who received each award<br>• Grant numbers awarded to each author<br>• The full name of each funder<br>• URL of each funder website<br>• Did the sponsors or funders play any role in the study design, data collection and analysis, decision to publish, or preparation of the manuscript?<br>• **NO** - Include this sentence at the end of your statement: *The funders had no role in study design, data collection and analysis, decision to publish, or preparation of the manuscript.*<br>• **YES** - Specify the role(s) played.<br><br>* typeset	
**Competing Interests**<br><br>Use the instructions below to enter a competing interest statement for this submission. On behalf of all authors, disclose any competing interests that could be perceived to bias this work—acknowledging all financial support and any other relevant financial or non-financial competing interests.<br><br>This statement is required for submission and **will appear in the published article** if the submission is accepted. Please make sure it is accurate and that any funding sources listed in your Funding Information later in the submission form are also declared in your Financial Disclosure statement.	The authors have declared that no competing interests exist

View published research articles from *PLOS ONE* for specific examples.<br><br>**NO authors have competing interests**<br><br>Enter: *The authors have declared that no competing interests exist.*<br><br>**Authors with competing interests**<br><br>Enter competing interest details beginning with this statement:<br><br>*I have read the journal's policy and the authors of this manuscript have the following competing interests: [insert competing interests here]*<br><br>* typeset	
**Ethics Statement**<br><br>Enter an ethics statement for this submission. This statement is required if the study involved:<br><br>• Human participants<br>• Human specimens or tissue<br>• Vertebrate animals or cephalopods<br>• Vertebrate embryos or tissues<br>• Field research<br><br>Write "N/A" if the submission does not require an ethics statement.<br><br>General guidance is provided below. Consult the submission guidelines for detailed instructions. **Make sure that all information entered here is included in the Methods section of the manuscript.**	The study was approved by the National Bioethics Committee of Chad (N° 201/PR/MESRI/DG/CNBT/2020, November 8, 2020) and the UCAD Research Ethics Committee (CER /UCAD/AD/MSN/050/2020, December 07, 2021). Prior to the study, informed consent was obtained from all participants in accordance with the approval of the Chad National Bioethics Committee and the UCAD Research Ethics Committee

**Format for specific study types**<br><br>**Human Subject Research (involving human participants and/or tissue)**<br>• Give the name of the institutional review board or ethics committee that approved the study<br>• Include the approval number and/or a statement indicating approval of this research<br>• Indicate the form of consent obtained (written/oral) or the reason that consent was not obtained (e.g. the data were analyzed anonymously)<br><br>**Animal Research (involving vertebrate animals, embryos or tissues)**<br>• Provide the name of the Institutional Animal Care and Use Committee (IACUC) or other relevant ethics board that reviewed the study protocol, and indicate whether they approved this research or granted a formal waiver of ethical approval<br>• Include an approval number if one was obtained<br>• If the study involved *non-human primates*, add *additional details* about animal welfare and steps taken to ameliorate suffering<br>• If anesthesia, euthanasia, or any kind of animal sacrifice is part of the study, include briefly which substances and/or methods were applied<br><br>**Field Research**<br><br>Include the following details if this study involves the collection of plant, animal, or other materials from a natural setting:<br>• Field permit number<br>• Name of the institution or relevant body that granted permission	
**Data Availability**<br><br>Authors are required to make all data underlying the findings described fully available, without restriction, and from the time of publication. PLOS allows rare exceptions to address legal and ethical concerns. See the PLOS Data Policy and FAQ for detailed information.	Yes - all data are fully available without restriction

A Data Availability Statement describing where the data can be found is required at submission. Your answers to this question constitute the Data Availability Statement and **will be published in the article**, if accepted.<br><br>**Important:** Stating 'data available on request from the author' is not sufficient. If your data are only available upon request, select 'No' for the first question and explain your exceptional situation in the text box.<br><br>Do the authors confirm that all data underlying the findings described in their manuscript are fully available without restriction?	
**Describe where the data may be found in full sentences. If you are copying our sample text, replace any instances of XXX with the appropriate details.**<br><br>• If the data are **held or will be held in a public repository**, include URLs, accession numbers or DOIs. If this information will only be available after acceptance, indicate this by ticking the box below. For example: *All XXX files are available from the XXX database (accession number(s) XXX, XXX.).*<br>• If the data are all contained **within the manuscript and/or Supporting Information files**, enter the following: *All relevant data are within the manuscript and its Supporting Information files.*<br>• If neither of these applies but you are able to provide **details of access elsewhere**, with or without limitations, please do so. For example:<br><br>*Data cannot be shared publicly because of [XXX]. Data are available from the XXX Institutional Data Access / Ethics Committee (contact via XXX) for researchers who meet the criteria for access to confidential data.*<br><br>*The data underlying the results presented in the study are available from (include the name of the third party*	All relevant data are within the manuscript and its Supporting Information files.

Powered by Editorial Manager® and ProduXion Manager® from Aries Systems Corporation

and contact information or URL).<br>• This text is appropriate if the data are owned by a third party and authors do not have permission to share the data.<br><br>* typeset	
Additional data availability information:	Tick here if the URLs/accession numbers/DOIs will be available only after acceptance of the manuscript for publication so that we can ensure their inclusion before publication.

Prof Emily Chenette

Editor

Plos One

February 2024

PONE-D-22-22361R1: Prevalence of hepatitis B virus infection and its associated factors among students in N'Djamena, Chad

Dear Prof Emily Chenette,

On behalf of all the co-authors we would like to thank you for this third opportunity to submit a revised version of our manuscript entitled "Prevalence of hepatitis B virus infection and its associated factors among students in N'Djamena, Chad." to Plos One. We appreciate the time and effort that you and the reviewers had dedicated to providing your valuable feedback on the last version of our manuscript and thank the last reviewer for the additional constructive observations provided for our previous submission. We believe the refinements made here have improved the manuscript even further. All second-round and third round changes are incorporated in the single original version as requested by the Editor. We have attached a marked-up copy of the manuscript labeled "Revised Manuscript with Track Changes" and an unmarked clean version of the manuscript labeled "Manuscript." We have also attached a point-by-point response to the last reviewers' comments in the file labeled "Response to Reviewers."

Yours sincerely,

Nalda Debsikréo (on behalf of all authors)

Manuscrit
Click here to access/download;Manuscript;Manuscript.doc

Title: Prevalence of hepatitis B virus infection and its associated factors among students in N'Djamena, Chad

Short title: Hepatitis B virus and associated factors among Chadian students

Authors: Nalda Debsikréo[1,2]*, Birwé Léon Mankréo[1], Azoukalné Moukénet[1,3], Merwa Ouangkake[4], Nathan Mara[4], Ali Mahamat Moussa[3,5], Ndèye Coumba Toure-Kane[1,2], Françoise Lunel-Fabiani[6]

Affiliations

[1] Cheikh Anta Diop University, Dakar, Senegal

[2] Institut de Recherche en Santé, de Surveillance Épidémiologique et de Formation, Dakar, Sénégal

[3] University of N'Djamena, N'Djamena, Chad

[4] Hôpital de la Paix, N'Djamena, Chad

[5] Centre Hospitalier Universitaire la Référence, N'Djamena, Chad

[6] Centre Hospitalier Universitaire Angers, BAT IBS-4 rue Larrey-49000 ANGERS, Laboratoire HIFIH, UFR Santé département Médecine, SFR 4208-UPRES EA3859, Université d'Angers, 49045 Angers Cedex 01, France

Corresponding author: * ndaldaeliane@gmail.com (ND)

Abstract

Introduction

Infection by hepatitis B virus (HBV) is a major issue in public health. The prevalence of HBV in Chad is 12.4%, all age groups considered. Here, we aimed to determine the prevalence of HBV and its associated factors among university students in N'Djamena, the country's capital.

Methods

A cross-sectional survey of students at either the University of N'djamena or Emi Koussi University was conducted from 3 to 23 July 2021. All participating students provided signed, informed consent and were included in the study consecutively. Blood samples were collected, and serum tested for hepatitis B surface antigen (HBsAg) using the Determine HBsAg rapid test kit, with confirmation of positive tests on an Abbott Architect i1000SR analyzer. Descriptive analysis and logistic regression were used to determine associations between the outcome variable and independent/covariate variables.

Results

A total of 457 students with a median age of 24 years were included across different faculties. The prevalence of HBV infection was 14.87% (68/457). Most students (75%) were aged 25 years or less. Unprotected sex was reported by 64.9% of the students and multiple sexual partners by 53.6%. Furthermore, 45.7% of them reported having no knowledge of hepatitis B. Having an HBsAg-positive mother (AOR: 2.11), having a history of transcutaneous medical procedures (AOR: 2.97) and living with a family (AOR: 4.63) were significantly associated with HBV status. Age ≥26 years appeared as a protective factor (AOR = 0.41).

Conclusion

Our study detected a high, 14.87% prevalence of HBV infection among students in N'djamena, Chad, and shed light on its associated factors. HBV prevention strategies should include raising awareness among students, making full hepatitis vaccination mandatory before children begin school, promoting mass screening to identify & treat chronic HBV carriers and reduce transmission, and reducing the cost of vaccination.

KEY WORDS: hepatitis B, HBsAg, prevalence, associated factors, students, Chad.

Introduction

Despite the availability of a prophylactic vaccine, hepatitis B remains a major public health problem. It causes close to 900,000 deaths per year and thus counts among the leading causes of mortality worldwide [1]. Its causative agent, the hepatitis B virus (HBV), is a partially circular DNA virus belonging to the *Hepadnaviridae* family. It is a small, enveloped virus responsible for both acute (sometimes fulminant) and chronic hepatitis. Moreover, HBV has oncogenic potential [2]. Chronic HBV infection is widespread [3] and its overall prevalence is 3.2% [4].

Although data on hepatitis B in Africa is scarce, its prevalence ~~there~~ is ~~estimated to be~~ among the highest in the world. More than 80 million people are infected with HBV in Africa [5], representing a prevalence of 6.1% [6]. Furthermore, a high prevalence of hepatitis B surface antigen (HBsAg)—exceeding 8% in the general population—is observed in Sub-Saharan Africa [7]. Hepatitis B is underdiagnosed on the continent, stemming from the frequently silent nature of chronic HBV infection and a lack of public information on its prevention, risks and consequences. Poor general health and vaccinal coverage also contribute to an increased risk of HBV infection in the population [8], with the risk of developing hepatocellular carcinoma [9].

Testing and access to treatment remain limited in many low-income countries, including those of Africa, despite the high burden of HBV infection frequently observed in them. More than 124,000 Africans die each year from undetected and/or untreated hepatitis[10]. Considering these aspects, population screening for HBV infection is a major challenge along the path toward preventing, treating and potentially eliminating hepatitis B[11]. ~~The authors of~~ the 1990-to-2019 Global Burden of Disease Study on hepatitis B reported an all-ages

prevalence of 12.4% for the disease in Chad in 2019 [12]. This significant public health issue thus concerns Chad directly and requires active surveillance ~~there.~~ Infected individuals can transmit HBV to others via ~~such~~ biological fluids ~~as~~ blood and sexual secretions. In areas of high endemicity and in the perinatal period, ~~this~~ transmission of HBV may be vertical or horizontal [13]. Non-vertical transmission may occur through blood transfusions, blood exposure accidents for health professionals, the use of non-sterile equipment for tattooing, piercing, acupuncture or intravenous drug use, or unprotected sexual intercourse [14].

Among these latter aspects, unprotected sexual activity and substance use in particular may increase the likelihood of HBV infection among university students, who are generally young and apparently healthy [15]. In addition, a family history of HBV infection, sexual activity and socioeconomic conditions have been reported to be ~~the main~~ risk factors for HBV infection among students in Bangui [16] and asymptomatic adolescents in Nigeria [17].

In the 2000s and with the goal of preventing transmission of HBV during childhood, several sub-Saharan countries began adding the HBV vaccine to their Expanded Programs on Immunization. This includes Chad, where the vaccine was added to its national plan in 2008 [18]. Despite this policy, the prevalence of HBV infection continues to increase, especially in the population over 15 years of age (incidence of 26.46% versus a national incidence of 13.62%)[19]. This aspect could hamper the attainment of National Health Plan targets to eliminate viral hepatitis, water-borne diseases and other communicable diseases by 2030 [20]. Studies on the burden of HBV infection among the student population and factors associated with hepatitis B are limited in this community, making it necessary to conduct studies to guide policies for the prevention of this scourge in Chad. Thus, ~~with the work~~

presented here, we aimed to determine the prevalence of HBV infection and identify associated factors among a sampling of students in N'Djamena, the capital city of Chad.

Methods and Materials

Study design

A cross-sectional study was conducted at the University of N'djamena (a public institution) and Emi Koussi University (a private institution, also located in N'djamena) from 3 to 23 July 2021. These institutions were selected because they have the largest number of students and N'Djamena is the city with the highest incidence of HBV (42.16%) in Chad [19]. The University of N'Djamena is the largest public university in the country, counting 16,142 students, seven faculties and four campuses [21]. Emi Koussi University is one of the largest private universities with 3,000 students approximately, two faculties and two campuses [22].

Inclusion criteria

Lists of all faculties of the two universities were obtained from their respective administrations. Based on enrollment in them, students who accepted to participate in the study were enrolled consecutively irrespective of age, sex, other sociodemographic characteristics or risk factors.

Sample size determination and sampling technique

A single-population sample size was calculated using the formula $n = \frac{z^2pq}{i^2}$. Given that there is no similar study in a student environment in Chad, a prevalence of 50%, a Z score value of 1.96 with an alpha level of 0.05 (95% confidence) and a 10% relative precision were used. This yielded a minimum sample size of 422 students and we ultimately interviewed and collected samples from 457 students. The final sample size was distributed proportionally to

the number of students in each faculty: Faculty of Languages, Letters, Arts and Communication (75 students); Faculty of Educational Sciences (80 students); Faculty of Exact and Applied Sciences (50 students); Faculty of Economics and Management (88 students); Faculty of Human and Social Sciences (75 students) and Emi Koussi University (50 students).

Data collection method

A structured questionnaire was developed based on that of Rajamoorthy *et al* [23]. It was used to collect information on socio-demographic characteristics and was administered during face-to-face meetings with the students. The questionnaire included age, sex, marital status, place of origin, faculty and study level, and risk factors, such as number of sexual partners, history of blood transfusion, surgery or hospitalization, HBsAg status of the mother, family history of HBV infection, HBsAg status of family members, history of traditional transcutaneous exposures such as piercings, tattoos, scarification, circumcision or excision (grouped as “risky practices”), history of transcutaneous medical procedures (including acupuncture), drug use, prison stays, unprotected sex, alcohol abuse, sharp object wounds, mode of residence, density of residence, clothes sharing, and sleeping arrangements.

Study variables

In light of a literature review [24, 25, 26], the following variables were included in this study: sex, age, marital status, educational level and study site. With regard to risk factors, the following potential variables were retained: student's life history, including number of sexual partners, history of blood transfusion, surgery, hospitalization, maternal HBsAg status, family history of HBV infection, risky practices, transcutaneous medical procedures, drug use,

prison stay, unprotected sex, alcohol, sharp injuries, density of residence, living conditions, sharing of clothing, living in a family, history of hepatitis B testing.

Specimen collection and processing

The study participants had a 5 ml venous blood sample drawn by a lab technician in an EDTA tube. The blood samples were labeled with unique numerical identifiers and coagulated. Sera were separated by centrifugation at 3000 rpm for 5 minutes and placed into Eppendorf tubes. HBsAg was searched for in the sera samples using the qualitative, rapid-detection immunochromatographic ALERE Determine HBsAg test, providing sensitivity and specificity of 95.3% and 93.3% respectively according to the manufacturer [27]. Positive results were confirmed on an Abbott Architect i1000SR analyzer (Abbott Diagnostics, Abbott Park, IL, USA) via chemiluminescent microparticle immunoassay. After HBsAg screening, sera samples were frozen and stored at -80°C in the biobank laboratory of the national reference hospital.

Data analysis

Descriptive analysis and logistic regression were used to examine associations between the outcome variable and independent/covariate variables. Quantitative variables were expressed as mean ± SD. Categorical variables were expressed as figures and percentages. The independent t-test was used to compare the means of continuous variables. When normal distribution or equal variance could not be assumed, the Mann-Whitney U test was used. The chi-squared test was used to compare proportions. For multivariate analysis, logistic regression analysis was performed to identify factors associated with HBV serological status among students. The regression was performed in two selection stages. The first consisted of automatic selection. The variables were selected according to the statistical selection criteria by a descending stepwise procedure. The model was selected by excluding

the non-significant variables, using Akaike's information criterion as an index of parsimony. In this first stage of selection, five variables were selected, namely age, history of surgery, maternal HBV, transcutaneous medical procedures and type of housing. In the second stage, variables with a p-value <0.3 in univariate analysis were included in the initial multivariate logistic regression model [28]. Gender was included as a forced variable in the multivariate analysis, even though it had a p-value >0.3 in the univariate analysis. In this second stage, the variables marital status and injected-drug use were selected. Adjusted odds ratios (AORs) were calculated to test the statistical associations between the dependent and independent variables, after adjustment for the other selected variables. Those with a p-value <0.05 were considered statistically significant. A 95% confidence interval was calculated for all odds ratios. All analyses were performed using R version 4.1.2.

Ethical considerations

The study was approved by the National Bioethics Committee of Chad (N° 201/PR/MESRI/DG/CNBT/2020, 8 November 2020) and the UCAD Research Ethics Committee (CER /UCAD/AD/MSN/050/2020, 7 December 2021). Written, informed and signed consent was obtained from all participants before inclusion in the study. The objectives of the survey were presented to the deans of the concerned faculties. All participants were informed of their serological results and provided with information on the possible consequences of HBV infection and, if necessary, the measures to be taken. The subjects who tested positive for HBV were referred to the program “Fight Against Hepatitis in Chad” for advice and follow-up, if warranted.

Results

Socio-demographic characteristics

Of the 457 students who participated in the study, 348 (76%) were male and 109 (24%) female, i.e., a sex ratio (M/F) of 3.19. Their median age was 24 years and a majority (75%) were aged less than 25 years. The majority of the participants were from the University of N'djamena (407, 89%). Also, most were single (348, 76%) **(Table 1).**

Table 1: Socio-demographic characteristics of study participants

Characteristic	Category	Frequency	Percent (%)
Gender	Female	109	23.85
	Male	348	76.14
Age	≤25	343	75.05
	>26	114	24.94
Site	FLLAC	75	16.41
	FES	99	21.66
	FEAS	50	10.94
	FEM	100	21.88
	FHSS	83	18.13
	EKU	50	10.94
Study level	Bachelor 1	141	30.85
	Bachelor 2	143	31.29
	Bachelor 3	168	36.76
	Master & PhD	5	1.09
Marital status	Married	109	23.85
	Single	348	76.14

FLLAC (Faculty of Languages, Letters, Arts and Communication), FES (Faculty of Educational Sciences), FEAS (Faculty of Exact and Applied Sciences), FEM (Faculty of Economics and Management), FHSS (Faculty of Humanities and Social Sciences), EKU (Emi-Koussi University).

Prevalence of HBV and its associated risk factors

Of the 457 students participating in the study, 68 (14.87 %((95% CI = 13.9–21.7%)) were positive for HBsAg. Table 2 depicts the percentage of people interviewed who displayed risk

factors and the prevalence of HBsAg among them. HBsAg positivity was highest among students living with a family (95.6%, n=65), followed by students having unprotected sex (65%, n=44), having multiple sexual partners (57%, n=39), living in a density of residence of more than two (50%, n=34), sharing sharp objects (46%, n=31) and having an HBsAg-positive mother (34%, n=23). Of the 107 (23%) students who had undergone a screening test for hepatitis B previously, 25 were HBsAg positive (37%) *(***Table 2).**

Table 2: Prevalence of HBV risk factors

**Characteristic**	**Overall n (%)**	**HBsAg positive n (%)**	**p-value**
Knowledge of hepatitis B	248 (54.26%)	38 (55.9%)	0.8
History of blood transfusion	58 (12.69%)	10 (14.7%)	0.6
Multiple sexual partners	252 (55.14%)	39 57.4%)	0.7
History of hospital admission	108 (26.63%)	16 (23.5)	>0.9
HBsAg-positive mother	102 (22.31%)	23 (33.8%)	0.014
Family history of hepatitis B	71 (15.53%)	15 (22.05%)	0.3
Transcutaneous medical procedures	65 (14.22%)	18 (26.5%)	0.002
History of drug injection	15 (3.3%)	1 (1.5%)	0.7
Risky practices (piercings, tattoos, etc.)	136 (29.75%)	21 (30.88%)	0.8
Jail stay	28 (6.1%)	5 (7.4%)	0.6
Unprotected sex	297 (64.98%)	44 (64.7%)	>0.9
Sharing of sharp materials	186 (40.70%)	31 (45.6%)	0.4
Housing style: family	353 (77.24%)	65 (95.6 %)	0.004
Density of residence: >2	245 (53.61%)	34 (50. 00 %)	0.5
History of hepatitis B testing	107 (23.41%)	25 (36.76%)	0.005*

Factors associated with HBV status in univariate and multivariate analyses

Logistic regression was used to test the association between HBV infection and the independent variables and to identify risk factors. Considering a p-value <0.30 in bivariate analysis, having an HBsAg-positive mother, a family history of HBV infection, a history of transcutaneous medical procedures, housing style and a history of HBV screening were identified as candidate variables for multivariate analysis. Having an HBsAg-positive mother (AOR: 2.11), having a history of transcutaneous medical procedures (AOR: 2.97), and living

with a family (AOR: 4.63) were significantly associated with HBV status, whereas age ≥26 years was a protective factor (AOR = 0.41) **(Table 3)**.

Table 3: Factors associated with HBV status of study participants in univariate and multivariate analysis.

Variable	Category	Overall n (%)	HBsAg prevalence n (%)	Univariable logistic regression		Multivariable logistic regression	
				**COR [95% CI]**	**P**	**AOR [95% CI])**	**P**
Sex	Female	109 (23.85)	16 (23.5)	1		1	
	Male	348 (76.14)	52 (76.5)	1.11 (0.58-2.22)	0.761	-	
Age	≤ 25	343 (75.05)	56 (82.4)	1		1	
	≥ 26	114 (24.94)	12 (17.6)	0.39 (0.17-0.82)	0.018	0.41 (0.18-0.85)	0.022*
Marital status	Married	109 (23.85)	17 (25.0)	1		1	
	Single	348 (76.14)	51 (75.0)	0.58 (0.28-1.22)	0.145	0.65 (0.33-1.31)	0.219
History of surgical procedure	No	435 (95.1)	68 (100.00)	1		1	
	Yes	22 (4.8)		0.00 (0.00-9467)	0.984	-	
History of blood transfusion	No	399 (87.3)	58 (85.3)	1		1	
	Yes	58 (12.69)	10 (14.7)	1.50 (0.64-3.29)	0.326	-	
Multiple sexual partners	>1	252 (55.14)	39 (57.4)	1		1	
	1	205 (44.85)	29 (42.6)	0.96 (0.53-1.73)	0.904	-	
Risky practices (piercings, tattoos, etc.)	No	321 (70.24)	47 (69.1)	1		1	
	Yes	136 (30 29.75)	21 (30.9)	0.93 (0.48-1.74)	0.821	-	
History of hospital admission	No	349 (76.36)	52 (76.5)	1		1	
	Yes	108 (23.63)	16 (23.5)	1.06 (0.53-2.03)	0.869	-	
HBsAg-positive mother	No	355(77.68)	45 (66.2)	1		1	
	Yes	102 (22.31)	23 (33.8)	2.03 (1.09-3.74)	0.023	2.11 (1.16-3.78)	0.013*
History of drug injection	No	442 (96.7)	67 (98.5)	1		1	
	Yes	15 (3.3)	1 (1.5)	0.28 (0.01-1.65)	0.246	0.29 (0.02-1.59)	0.244
Jail stay	No	429 (93.8)	63 (92.6)	1		1	
	Yes	28 (6.1)	5 (7.4)	0.85 (0.24-2.50)	0.785	-	
Unprotected sex	No	160(35.01)	24 (35.3)	1		1	
	Yes	297 (64.98)	44 (64.7)	0.95 (0.52-1.77)	0.869	-	
Transcutaneous medical procedures	No	392 (85.77)	50 (73.5)	1		1	
	Yes	65 14.23)	18 (26.5)	3.14 (1.54-6.34)	0.001	2.97 (1.51-5.76)	0.001*
Sharing of sharp materials	No	271 (59 59.29)	37 (54.4)	1		1	
	Yes	186 (40.70)	31 (45.6)	1.33 (0.75-2.34)	0.327		
Housing style	Alone	48 (12.3)	3 (4.4)	1		1	
	Family	353 (77.24)	65 (95.6)	4.54 (1.44-20.47)	0.022	4.63 (1.50-20.52)	0.018*
Density of residence	1-2	212 (46.38)	34 (50.00)	1.49 (0.83-2.70)	0.186	1.45 (0.81-2.58)	0.210
	>2	245 (53.61)	34 (50.00)	1		1	

95% CI = 95% Confidence Interval, COR: crude odds ratio, AOR: adjusted odds ratio, 1: reference category, *: significantly associated factors p-value <0.05

Discussion

In our study, we assessed the prevalence of HBV and its associated factors among students in the main public university and the main private university in N'Djamena, Chad. Our results show a prevalence of HBV infection (HBsAg positivity) of 14.87% (68/457). That finding places our study population in the high prevalence group, i.e., well above the 8% threshold established by the World Health Organization [29].

The HBsAg positivity prevalence found in our study is also higher than the overall prevalence of 12.4% reported for all age groups in Chad, and over those reported for some other population groups in N'Djamena, for example 7.2% in pregnant women [30] and 13.5% in HIV-infected patients [31]. Our higher prevalence may be due to the fact that our students were largely part of a relatively young—but not young enough—population. Indeed, with an average age of 24±3 years, our subjects most likely did not benefit from vaccination against HBV in childhood as part of the Expanded Program on Immunization introduced in 2008 [18]. Outside of that program, the relatively onerous price of the HBV vaccine is not covered by the current Chadian healthcare system, which acts as a barrier to large coverage in this population.

Moreover, the HBV prevalence found in our population is higher than those reported in studies on students from Togo (4.6%) [25], Cameroon (5.6%) [32] and Ethiopia (11.5%) [33]. It is lower however than that reported in Nigeria (31.5%) (34) and comparable to that reported in the Central African Republic (15.5%) [16].

Currently, Chadian students are not provided with prevention information (e.g. on sexuality, sexually transmitted diseases and risky practices), which most likely plays a part in that high prevalence rate. The environment in which students live may also carry risk factors

(presence of drugs, alcohol, sex, etc.) [15] and the students themselves are in a period of life where risk taking may be more common, as compared to other age groups. Furthermore, sex education continues to be a taboo subject in Chadian families.

Of the 107 students (23%) who had already been tested for HBV infection in the past, 25 (37%) tested positive in our study. That finding is consistent with data reported by Halatoko *et al* in Togo [26]. Our interpretation is that students who knew they were already infected might have been particularly tempted to participate in the study, hoping to receive treatment or to check their serological status.

Focusing on the management and clinical follow-up for HBsAg-positive students, and encouraging those who tested HBsAg and anti-HBc-antibody-negative to complete the vaccination course are both critical. All participants in our study received information on HBV infection and those who tested positive were referred to health facilities for follow-up.

Our data showed that maternal HBV status was significantly associated with HBsAg carriage among our students, likely explained by mother-to-child (vertical) transmission risks. Results similar to ours were reported in Gambia in 2007, where vertical transmission was responsible for 16% of chronic infections [35] and in Cameroon, where the risk of vertical transmission was substantial despite birth-dose vaccination [36]. These data confirm the hypothesis that in sub-Saharan Africa, a highly endemic region for HBV, vertical transmission merits as much attention as its horizontal counterpart [37].

Our study reported a significantly higher risk of transmission among students living in families, with a seroprevalence of 95.6% (65/68) in this study group. These results corroborate those obtained in the Central African Republic [16] , where intrafamilial transmission was reported to be associated with hepatitis B in students. This association is probably due to contact between HBV-infected family members [38], the exchange of sharp

objects and the sharing of personal-use items (those causing skin and mucous-membrane lesions), and a lack of knowledge on the different modes of transmission of HBV. For these reasons, public awareness and systematic screening of those living with infected people are essential.

A history of transcutaneous medical procedures was found to be a risk factor for hepatitis B in this study. This could be due to nosocomial transmission of HBV (resulting from non-compliance with universal recommendations to prevent postoperative infections and cross-contamination of equipment and medical devices) [39].

According to the multivariate analysis, students aged 26 years or more were less likely to be infected with HBV than those aged 25 years or less, suggesting age as a protective factor. Similar results were reported by Samsunder *et al* in South Africa [34], where participants aged 20–24 years were more exposed than other groups. It is possible that the older students had already been infected during childhood or adolescence but cleared that earlier HBV more or less quickly, and thus did not developed chronic infection. Unfortunately, we were unable to check for anti-HBc antibodies (Ab) to confirm this hypothesis. Similarly, we did not check for anti-HBs Ab, which could have given us an idea of the percentage of vaccinated students.

Strengths and limitations

Our study adds to data regarding prevalence of hepatitis B virus infection in Chad. Such findings are expected to guide public health strategies for the diagnosis of HBV pathologies among students and the general population. Although our work involved a mixed population of students from both the University of N'djamena and Emi-Koussi University, it does have certain limitations. As underlined above, we were unable to carry out total anti-HBc Ab, anti-

HBc IgM Ab and anti-HBs Ab testing. This would have allowed for a distinction between subjects who were acutely infected (with anti-HBc IgM Ab), chronic carriers, showing recovery from an acute or chronic infection (anti-HBc Ab positive) [40, 41], or immunized against HBV (anti-HBs Ab positive). The face-to-face nature of our interviews may represent a limitation and constraints in the study design may have introduced selection bias

Logistic regression was adjusted for other variables but only ten were taken into account. This may represent a limit on the control of confounding factors

Conclusion and Recommendations

Our study showed that the prevalence of HBV infection in students was higher than the overall prevalence in Chad. Having an HBsAg-positive mother, a history of transcutaneous medical procedures, living with a roommate, and living with a family were significantly associated with HBV status. In view of our results, HBV prevention and treatment policies should include: awareness campaigns to inform at-risk populations like students on HBV transmission pathways; HBV vaccination at birth; mandatory full hepatitis vaccination status before children start school; the promotion of mass screening to identify & treat chronic HBV carriers and reduce transmission; reduction of vaccination costs; and the promotion of free-of-charge HBV screening for pregnant women.

List of abbreviations

Anti-HBs: Hepatitis B surface antibody, AOR: Adjusted odd ratio; CI: Confidence interval, COR: Crude odd ratio, HBsAg: Hepatitis B surface antigen, HBV: Hepatitis B virus.

Inclusivity in global research

Additional information regarding the ethical, cultural, and scientific considerations specific to inclusivity in global research is included in the Supporting Information (S2 Checklist).

Supporting information

S1 Checklist. Inclusivity in global research questionnaire. (DOCX)

S2 Data. (XLSX)

Acknowledgements

We thank the laboratory technicians at Hôpital de la Paix in N'djamena who took the blood samples and performed the various tests. We express our gratitude to the survey's data collectors and supervisors. We furthermore thank the deans of Cheikh Anta Diop University in Dakar for their guidance. We express our gratitude both to the student secretaries who facilitated participation in the study and particularly to the students who joined us for it.

Availability of data and materials

The datasets generated and/or analyzed during the current study are available in the Mendeley repository here .

Conflicts of interest

The authors have declared that no competing interests exist.

Funding

The authors received no specific funding for this work.

Author Contributions

Conceptualization: Nalda Debsikréo, Françoise Lunel-Fabiani

Data curation: Nalda Debsikréo, Birwé Léon Mankréo, Azoukalné Moukénet

Formal analysis: Nalda Debsikréo, Birwé Léon Mankréo, Azoukalné Moukénet

Funding acquisition: Nalda Debsikréo

Investigation: Nalda Debsikréo, Merwa Ouangkake, Nathan Mara, Ali Mahamat Moussa

Methodology: Nalda Debsikréo, Birwé Léon Mankréo, Azoukalné Moukénet, Ali Mahamat Moussa Ndèye Coumba Toure-Kane, Françoise Lunel-Fabiani

Project administration: Nalda Debsikréo, Merwa Ouangkake, Nathan Mara, Ali Mahamat Moussa

Software: Nalda Debsikréo, Birwé Léon Mankréo, Azoukalné Moukénet

Laboratory: Nalda Debsikréo, Merwa Ouangkake, Nathan Mara

Validation: Nalda Debsikréo; Birwé Léon Mankréo, Azoukalné Moukénet, Merwa Ouangkake, Nathan Mara, Ali Mahamat Moussa, Ndèye Coumba Toure-Kane, Françoise Lunel-Fabiani

Visualization: Nalda Debsikréo, Birwé Léon Mankréo, Azoukalné Moukénet

Writing – original draft: Nalda Debsikréo, Françoise Lunel-Fabiani

Writing – review & editing: Nalda Debsikréo, Birwé Léon Mankréo, Azoukalné Moukénet, Merwa Ouangkake, Nathan Mara, Ali Mahamat Moussa, Ndèye Coumba Toure-Kane, Françoise Lunel-Fabiani

References

1. Stanaway JD, Flaxman AD, Naghavi M, Fitzmaurice C, Vos T, Abubakar I, et al. The global burden of viral hepatitis from 1990 to 2013: findings from the Global Burden of Disease Study 2013. The Lancet. 10 sept 2016; 388(10049):1081‑8.

2. Selzer L, Zlotnick A. Assembly and Release of Hepatitis B Virus. Cold Spring Harb Perspect Med. déc 2015; 5(12):a021394.

3. Global hepatitis report, 2017 [Internet]. [[Cited 26 March 2023].]. https://www.who.int/publications-detail-redirect/9789241565455

4. Polaris Observatory Collaborators. Global prevalence, cascade of care, and prophylaxis coverage of hepatitis B in 2022: a modelling study. Lancet Gastroenterol Hepatol. oct 2023;8(10):879‑907.

5. Gnyawali B, Pusateri A, Nickerson A, Jalil S, Mumtaz K. Epidemiologic and socioeconomic factors impacting hepatitis B virus and related hepatocellular carcinoma. World J Gastroenterol. 7 août 2022; 28(29):3793‑802.

6. Spearman CW, Afihene M, Ally R, Apica B, Awuku Y, Cunha L, et al. Hepatitis B in sub-Saharan Africa: strategies to achieve the 2030 elimination targets. Lancet Gastroenterol Hepatol. 1 déc 2017; 2(12):900‑9.

7. Polaris Observatory Collaborators. Global prevalence, treatment, and prevention of hepatitis B virus infection in 2016: a modelling study. Lancet Gastroenterol Hepatol. juin 2018;3(6):383‑403.

8. Lu PJ, O'Halloran AC, Williams WW, Nelson NP. Hepatitis B vaccination coverage among adults aged ≥ 18 years traveling to a country of high or intermediate endemicity, United States, 2015. Vaccine. 25 avr 2018; 36(18):2471‑9.

9. Park Y, Lee JH, Sinn DH, Park JY, Kim MA, Kim YJ, et al. Risk and Risk Score Performance of Hepatocellular Carcinoma Development in Patients With Hepatitis B Surface Antigen Seroclearance. Clin Transl Gastroenterol. 11 janv 2021; 12(1):e00290.

10. WHO. WHO | Regional Office for Africa. [Cited 22 June 2022]. World Hepatitis Day, 28 July 2021. https://www.afro.who.int/regional-director/speeches-messages/world-hepatitis-day-28-july-2021

11. Abbas Z, Abbas M. Challenges in Formulation and Implementation of Hepatitis B Elimination Programs. Cureus. 13(4):e14657.

12. GBD 2019 Hepatitis B Collaborators. Global, regional, and national burden of hepatitis B, 1990-2019: a systematic analysis for the Global Burden of Disease Study 2019. Lancet Gastroenterol Hepatol. sept 2022;7(9):796‑829.

13. Zampino R, Boemio A, Sagnelli C, Alessio L, Adinolfi LE, Sagnelli E, et al. Hepatitis B virus burden in developing countries. World J Gastroenterol. 14 nov 2015;21(42):11941‑53.

14. Tao I, Compaoré TR, Diarra B, Djigma F, Zohoncon TM, Assih M, et al. Seroepidemiology of hepatitis B and C viruses in the general population of burkina faso. Hepat Res Treat. 2014; 2014:781843.

15. Gülsoy SS, Siyez E, Esen E, Baran B, Siyez DM. Predictors of self-reported sexually transmitted infections among Turkish university students. J Dtsch Dermatol Ges J Ger Soc Dermatol JDDG. juill 2023;21(7):751‑8.

16. Komas NP, Baï-Sepou S, Manirakiza A, Léal J, Béré A, Le Faou A. The prevalence of hepatitis B virus markers in a cohort of students in Bangui, Central African Republic. BMC Infect Dis. 29 juill 2010; 10:226.

17. Njoku OO. Prevalence of assymptomatic hepatitis B virus among sexually active youths in a rural community of Ebonyi state, Southeast Nigeria. Int J Infect Dis. 1 avr 2016; 45:450‑1.

18. Ferrinho P, Dramé M, Tumusiime P. Perceptions of the usefulness of external support to immunization coverage in Chad: an analysis of the GAVI-Alliance cash-based support. Pan Afr Med J. 7 juin 2013;15:44.

19. tcd_rpt_annuairesstatistiquessanitaires_ministeresante_2017.pdf [Internet]. [Cited 13 May 2022]. https://www.humanitarianresponse.info/sites/www.humanitarianresponse.info/files/documents/files/tcd_rpt_annuairesstatistiquessanitaires_ministeresante_2017.pdf

20. WATHI. Politique nationale de santé 2016-2030, ministère de la Santé publique du Tchad, 2016 [Internet]. WATHI. 2021 [cited 17 Feb 2023]. https://www.wathi.org/wathinote-election-tchad-situation-sanitaire/politique-nationale-de-sante-2016-2030-2016/

21. La formation à l'université de N'Djaména [Internet]. [Cited 23 april 2022]]. https://archive.wikiwix.com/cache/index2.php?url=https%3A%2F%2Fwww.universite-ndjamena.td%2Fformation#federation=archive.wikiwix.com

22. L'Université [Internet]. Unniversité Emikoussi. 2022 [cited 13 March 2023]. https://universite-emikoussi.net/luniversite/

23. Rajamoorthy Y, Taib NM, Munusamy S, Anwar S, Wagner AL, Mudatsir M, et al. Knowledge and awareness of hepatitis B among households in Malaysia: a community-based cross-sectional survey. BMC Public Health. 10 janv 2019; 19:47.

24. Mosaad M, Al Nozha OM, Yamany H, Amer S. A survey of hepatitis B immune status of Taibah University medical students. J Taibah Univ Med Sci. 1 déc 2014; 9(4):301‑6.

25. Ekouevi DK, Thomas A, Sewu D, Lawson-Ananissoh L, Tchounga B, Salou M, et al. Prevalence of Hepatitis B among Students from the University of Lomé, Togo in 2015. Open J Epidemiol. 8 août 2017; 7(3):262‑72.

26. Halatoko WA, Patassi A, Yanogo P, Banla LI, Koba A, Issa Z, et al. Risk factors of hepatitis B virus surface antigen carriage and serological profile of HBsAg carriers in Lomé Togo, 2016. BMC Public Health. 8 janv 2019; 19(1):32.

27. Njai HF, Shimakawa Y, Sanneh B, Ferguson L, Ndow G, Mendy M, et al. Validation of rapid point-of-care (POC) tests for detection of hepatitis B surface antigen in field and laboratory settings in the Gambia, Western Africa. J Clin Microbiol. avr 2015;53(4):1156‑63.

28. Ouoba S, Ko K, Lingani M, Nagashima S, Guingané AN, Bunthen E, et al. Intermediate hepatitis B virus infection prevalence among 1622 pregnant women in rural Burkina Faso and implications for mother-to-child transmission. Sci Rep. 14 avr 2023; 13(1):6115.

29. Guidelines for the prevention, care and treatment of persons with chronic hepatitis B infection [Internet]. [cited 13 jan 2024]. https://www.who.int/publications-detail-redirect/9789241549059

30. Debsikréo N, Mankréo BL, Moukenet A, Ndiaye AJS, Diouf NL, Lo G, et al. Prevalence of Hepatitis B and Associated Factors among Pregnant Women in N'djamena, Chad. Fortune J Health Sci. 13 juill 2023; 6(3):253‑62.

31. Bessimbaye N, Moussa AM, Mbanga D, Tidjani A, Mahamat SO, Ngawara MN, et al. [Seroprevalence of HBs Ag and of anti-HCV antibodies among HIV infected people in N'Djamena, Chad]. Bull Soc Pathol Exot 1990. déc 2014;107(5):327‑31.

32. servais albert fiacre E. Statut Vaccinal Contre le Virus de l'Hépatite Virale B et portage de l'Antigène Hbs chez les Etudiants en Médecine et en Pharmacie de l'Université de Douala au Cameroun. Health Sci Dis. 1 janv 2014;

33. Tesfa T, Hawulte B, Tolera A, Abate D. Hepatitis B virus infection and associated risk factors among medical students in eastern Ethiopia. PLOS ONE. 19 févr 2021; 16(2):e0247267.

34. Tula MY, Iyoha O. A Cross-sectional Study on the Sero-prevalence of Hepatitis B Surface Antigen (HBsAg) among Apparently Healthy Students of a Tertiary Institution in North-Eastern Nigeria. Int J Trop Dis Health. 10 janv 2015; 7:102‑8.

35. Shimakawa Y, Lemoine M, Njai HF, Bottomley C, Ndow G, Goldin RD, et al. Natural history of chronic HBV infection in West Africa: a longitudinal population-based study from The Gambia. Gut. 1 déc 2016;65(12):2007‑16.

36. Shimakawa Y, Veillon P, Birguel J, Pivert A, Sauvage V, Guillou-Guillemette HL, et al. Residual risk of mother-to-child transmission of hepatitis B virus infection despite timely birth-dose vaccination in Cameroon (ANRS 12303): a single-centre, longitudinal observational study. Lancet Glob Health. 1 avr 2022; 10(4):e521‑9.

37. Shimakawa Y, Toure-Kane C, Mendy M, Thursz M, Lemoine M. Mother-to-child transmission of hepatitis B in sub-Saharan Africa. Lancet Infect Dis. 1 janv 2016;16(1):19‑20.

38. Mast EE, Weinbaum CM, Fiore AE, Alter MJ, Bell BP, Finelli L, et al. A comprehensive immunization strategy to eliminate transmission of hepatitis B virus infection in the United States: recommendations of the Advisory Committee on Immunization Practices (ACIP) Part II: immunization of adults. MMWR Recomm Rep Morb Mortal Wkly Rep Recomm Rep. 8 déc 2006; 55(RR-16):1‑33; quiz CE1-4.

39. Sugimoto S, Nagakubo S, Ito T, Tsunoda Y, Imamura S, Tamura T, et al. A case of acute hepatitis B related to previous gynecological surgery in Japan. J Infect Chemother Off J Jpn Soc Chemother. juin 2013;19(3):524‑9.

40. Lau GKK. How do we handle the anti-HBc positive patient? (In highly endemic settings). Clin Liver Dis. 27 mars 2015; 5(2):29‑31.

41. Gachara G, Magoro T, Mavhandu L, Lum E, Kimbi HK, Ndip RN, et al. Characterization of occult hepatitis B virus infection among HIV positive patients in Cameroon. AIDS Res Ther. 8 mars 2017; 14:11.

Supporting information

S1 File. Questionnaire (docx)

381 S2 File. Checklist (docx)

Click here to access/download
Supporting Information
S1 Questionnaire.docx

Click here to access/download
Supporting Information
S2 Check list.docx

Click here to access/download
Supporting Information - Compressed/ZIP File Archive
Analysis results.docx

Click here to access/download

Supporting Information - Compressed/ZIP File Archive

BDE_Data_set.xlsx

Title: Prevalence of hepatitis B virus infection and its associated factors among students in N'Djamena, Chad.

Short title: Hepatitis B virus and associated factors among Chadian students

Authors: Nalda Debsikréo[1,2]*, Birwé Léon Mankréo[1], Azoukalné Moukénet[1,3], Merwa Ouangkake[4], Nathan Mara[4], Ali Mahamat Moussa[3,5], Ndèye Coumba Toure-Kane[1,2], Françoise Lunel-Fabiani[6]

Affiliations:

[1] Cheikh Anta Diop University, Dakar, Senegal

[2] Institut de Recherche en Santé, de Surveillance Épidémiologique et de Formation, Dakar, Sénégal

[3] University of N'Djamena, N'Djamena, Chad

[4] Hôpital de la Paix, N'Djamena, Chad

[5] Centre Hospitalier Universitaire la Référence, N'Djamena, Chad

[6] Centre Hospitalier Universitaire Angers, BAT IBS-4 rue Larrey-49000 ANGERS, Laboratoire HIFIH, UFR Santé département Médecine, SFR 4208-UPRES EA3859, Université d'Angers, 49045 Angers Cedex 01, France

Corresponding author: * ndaldaeliane@gmail.com (ND)

Abstract

Introduction

Infection by hepatitis B virus (HBV) is a major issue in public health. The prevalence of HBV in Chad is 12.4%, all age groups considered. Here, we aimed to determine the prevalence of HBV and its associated factors among university students in N'Djamena, the country's capital.

Methods

A cross-sectional survey of students at either the University of N'djamena or Emi Koussi University was conducted from 3 to 23 July 2021. All participating students provided signed, informed consent and were included in the study consecutively. Blood samples were collected, and serum tested for hepatitis B surface antigen (HBsAg) using the Determine HBsAg rapid test kit, with confirmation of positive tests on an Abbott Architect i1000SR analyzer. Descriptive analysis and logistic regression were used to determine associations between the outcome variable and independent/covariate variables.

Results

A total of 457 students with a ~~mean age of 24±3~~ median age of 24 years were included across different faculties. The prevalence of HBV infection was 14.87% (68/457). Most students (75%) were aged 25 years or less. Unprotected sex was reported by 64.9% of the students and multiple sexual partners by 53.6%. Furthermore, 45.7% of them reported having no knowledge of hepatitis B. Having an HBsAg-positive mother (AOR: 2.11), having a history of transcutaneous medical procedures (AOR: 2.97) and living with a family (AOR: 4.63) were significantly associated with HBV status. Age ≥26 years appeared as a protective factor (AOR = 0.41).

Conclusion

Our study detected a high, 14.87% prevalence of HBV infection among students in N'djamena, Chad, and shed light on its associated factors. HBV prevention strategies should include raising awareness among students, making full hepatitis vaccination mandatory before children begin school, promoting mass screening to identify & treat chronic HBV carriers and reduce transmission, and reducing the cost of vaccination.

KEY WORDS: hepatitis B, HBsAg, prevalence, associated factors, students, Chad.

Introduction

Despite the availability of a prophylactic vaccine, hepatitis B remains a major public health problem. It causes close to 900,000 deaths per year and thus counts among the leading causes of mortality worldwide [1]. Its causative agent, the hepatitis B virus (HBV), is a partially circular DNA virus belonging to the *Hepadnaviridae* family. It is a small, enveloped virus responsible for both acute (sometimes fulminant) and chronic hepatitis. Moreover, HBV has oncogenic potential [2]. Chronic HBV infection is widespread [3] and its overall prevalence is 3.2% [4].

Although data on hepatitis B in Africa is scarce, its prevalence there is estimated to be among the highest in the world. More than 80 million people are infected with HBV in Africa [5], representing a prevalence of 6.1% [6]. Furthermore, a high prevalence of hepatitis B surface antigen (HBsAg)—exceeding 8% in the general population—is observed in Sub-Saharan Africa [7]. Hepatitis B is underdiagnosed on the continent, stemming from the frequently silent nature of chronic HBV infection and a lack of public information on its prevention, risks and consequences. Poor general health and vaccinal coverage also contribute to an increased risk of HBV infection in the population [8], with the risk of developing hepatocellular carcinoma [9].

Testing and access to treatment remain limited in many low-income countries, including those of Africa, despite the high burden of HBV infection frequently observed in them. More than 124,000 Africans die each year from undetected and/or untreated hepatitis[10]. Considering these aspects, population screening for HBV infection is a major challenge along the path toward preventing, treating and potentially eliminating hepatitis B[11]. The authors of the 1990-to-2019 Global Burden of Disease Study on hepatitis B reported an all-ages

prevalence of 12.4% for the disease in Chad in 2019 [12]. This significant public health issue thus concerns Chad directly and requires active surveillance there. Infected individuals can transmit HBV to others via such biological fluids as blood and sexual secretions. In areas of high endemicity and in the perinatal period, this transmission of HBV may be vertical or horizontal [13]. Non-vertical transmission may occur through blood transfusions, blood exposure accidents for health professionals, the use of non-sterile equipment for tattooing, piercing, acupuncture or intravenous drug use, or unprotected sexual intercourse [14].

Among these latter aspects, unprotected sexual activity and substance use in particular may increase the likelihood of HBV infection among university students, who are generally young and apparently healthy [15]. In addition, a family history of HBV infection, sexual activity and socioeconomic conditions have been reported to be the main risk factors for HBV infection among students in Bangui [16] and asymptomatic adolescents in Nigeria [17].

In the 2000s and with the goal of preventing transmission of HBV during childhood, several sub-Saharan countries began adding the HBV vaccine to their Expanded Programs on Immunization. This includes Chad, where the vaccine was added to its national plan in 2008 [18]. Despite this policy, the prevalence of HBV infection continues to increase, especially in the population over 15 years of age (incidence of 26.46% versus a national incidence of 13.62%)[19]. This aspect could hamper the attainment of National Health Plan targets to eliminate viral hepatitis, water-borne diseases and other communicable diseases by 2030 [20]. Studies on the burden of HBV infection among the student population and factors associated with hepatitis B are limited in this community, making it necessary to conduct studies to guide policies for the prevention of this scourge in Chad. Thus, with the work

presented here, we aimed to determine the prevalence of HBV infection and identify associated factors among a sampling of students in N'Djamena, the capital city of Chad.

Methods and Materials

Study design

A cross-sectional study was conducted at the University of N'djamena (a public institution) and Emi Koussi University (a private institution, also located in N'djamena) from 3 to 23 July 2021. These institutions were selected because they have the largest number of students and N'Djamena is the city with the highest incidence of HBV (42.16%) in Chad [19]. The University of N'Djamena is the largest public university in the country, counting 16,142 students, seven faculties and four campuses [21]. Emi Koussi University is one of the largest private universities with 3,000 students approximately, two faculties and two campuses [22].

Inclusion criteria ~~Sample size determination and sampling technique~~

Lists of all faculties of the two universities were obtained from their respective administrations. Based on enrollment in them, students who accepted to participate in the study were enrolled consecutively irrespective of age, sex, other sociodemographic characteristics or risk factors.

Sample size determination and sampling technique

A single-population sample size was calculated using the formula $n = \frac{z^2pq}{i^2}$. Given that there is no similar study in a student environment in Chad, a prevalence of 50%, a Z score value of 1.96 with an alpha level of 0.05 (95% confidence) and a 10% relative precision were used. This yielded a minimum sample size of 422 students and we ultimately interviewed and collected

samples from 457 students. The final sample size was distributed proportionally to the number of students in each faculty: Faculty of Languages, Letters, Arts and Communication (75 students); Faculty of Educational Sciences (80 students); Faculty of Exact and Applied Sciences (50 students); Faculty of Economics and Management (88 students); Faculty of Human and Social Sciences (75 students) and Emi Koussi University (50 students).

Data collection method

A structured questionnaire was developed based on that of Rajamoorthy *et al* [23]. It was used to collect information on socio-demographic characteristics. and was administered during face-to-face meetings with the students The questionnaire included age, sex, marital status, place of origin, faculty and study level, and risk factors, such as number of sexual partners, history of blood transfusion, surgery or hospitalization, HBsAg status of the mother, family history of HBV infection, HBsAg status of family members, history of traditional transcutaneous exposures such as piercings, tattoos, scarification, circumcision or excision (grouped as “risky practices”), history of transcutaneous medical procedures (including acupuncture), drug use, prison stays, unprotected sex, alcohol abuse, sharp object wounds, mode of residence, density of residence, clothes sharing, and sleeping arrangements.

Study variables

In light of a literature review [24, 25, 26], the following variables were included in this study: sex, age, marital status, educational level and study site. With regard to risk factors, the following potential variables were retained: student's life history, including number of sexual partners, history of blood transfusion, surgery, hospitalization, maternal HBsAg status, family history of HBV infection, risky practices, transcutaneous medical procedures, drug use, prison

stay, unprotected sex, alcohol, sharp injuries, density of residence, living conditions, sharing of clothing, living in a family, history of hepatitis B testing.

Specimen collection and processing

The study participants had a 5 ml venous blood sample drawn by a lab technician in an EDTA tube. The blood samples were labeled with unique numerical identifiers and coagulated. Sera were separated by centrifugation at 3000 rpm for 5 minutes and placed into Eppendorf tubes. HBsAg was searched for in the sera samples using the qualitative, rapid-detection immunochromatographic ALERE Determine HBsAg test, providing sensitivity and specificity of 95.3% and 93.3% respectively according to the manufacturer [27]. Positive results were confirmed on an Abbott Architect i1000SR analyzer (Abbott Diagnostics, Abbott Park, IL, USA) via chemiluminescent microparticle immunoassay. After HBsAg screening, sera samples were frozen and stored at -80°C in the biobank laboratory of the national reference hospital.

Data analysis

Descriptive analysis and logistic regression were used to examine associations between the outcome variable and independent/covariate variables. Quantitative variables were expressed as mean ± SD. Categorical variables were expressed as figures and percentages. The independent t-test was used to compare the means of continuous variables. When normal distribution or equal variance could not be assumed, the Mann-Whitney U test was used. The chi-squared test was used to compare proportions. For multivariate analysis, logistic regression analysis was performed to identify factors associated with HBV serological status among students. The regression was performed in two selection stages. The first consisted of automatic selection. The variables were selected according to the statistical selection criteria by a descending stepwise procedure. The model was selected by excluding

the non-significant variables, using Akaike's information criterion as an index of parsimony. In this first stage of selection, five variables were selected, namely age, history of surgery, maternal HBV, transcutaneous medical procedures and type of housing. In the second stage, variables with a p-value <0.3 in univariate analysis were included in the initial multivariate logistic regression model [28]. Gender was included as a forced variable in the multivariate analysis, even though it had a p-value >0.3 in the univariate analysis. In this second stage, the variables marital status and injected-drug use were selected. Adjusted odds ratios (AORs) were calculated to test the statistical associations between the dependent and independent variables, after adjustment for the other selected variables. Those with a p-value <0.05 were considered statistically significant. A 95% confidence interval was calculated for all odds ratios. All analyses were performed using R version 4.1.2 ~~(2021 11 01)~~

Ethical considerations

The study was approved by the National Bioethics Committee of Chad (N° 201/PR/MESRI/DG/CNBT/2020, 8 November 2020) and the UCAD Research Ethics Committee (CER /UCAD/AD/MSN/050/2020, 7 December 2021). Written, informed and signed consent was obtained from all participants before inclusion in the study. The objectives of the survey were presented to the deans of the concerned faculties. All participants were informed of their serological results and provided with information on the possible consequences of HBV infection and, if necessary, the measures to be taken. The subjects who tested positive for HBV were referred to the program “Fight Against Hepatitis in Chad” for advice and follow-up, if warranted.

Results

Socio-demographic characteristics

Of the 457 students who participated in the study, 348 (76%) were male and 109 (24%) female, i.e., a sex ratio (M/F) of 3.19. Their ~~mean~~ median age was 24~~±3~~ years ~~(median=24)~~ and a majority (75%) were aged less than 25 years. The majority of the participants were from the University of N'djamena (407, 89%). Also, most were single (348, 76%) **(Table 1).**

Table 1: Socio-demographic characteristics of study participants

Characteristic	Category	Frequency	Percent (%)
Gender	Female	109	~~22~~23.85
	Male	348	~~76~~ 76.14
Age	≤25	343	~~75~~ 75.05
	>26	114	~~25~~ 24.94
Site	FLLAC	75	~~16~~16.41
	FES	99	~~22~~ 21.66
	FEAS	50	~~11~~ 10.94
	FEM	100	~~22~~21.88
	FHSS	83	~~18~~ 18.13
	EKU	50	~~11~~ 10.94
Study level	Bachelor 1	141	~~31~~ 30.85
	Bachelor 2	143	~~31~~31.29
	Bachelor 3	168	~~37~~36.76
	Master & PhD	5	~~1.1~~ 1.09
Marital status	Married	109	~~24~~ 23.85
	Single	348	~~76~~76.14

Formatted: Left, Tab stops: 0.33", Left + 0.53", Centered

Formatted: Left, Tab stops: 0.28", Left + 0.53", Centered

FLLAC (Faculty of Languages, Letters, Arts and Communication), FES (Faculty of Educational Sciences), FEAS (Faculty of Exact and Applied Sciences), FEM (Faculty of Economics and Management), FHSS (Faculty of Humanities and Social Sciences), EKU (Emi-Koussi University).

Prevalence of HBV and its associated risk factors

Of the 457 students participating in the study, 68 (14.87%(~~-~~(95% CI = 13.9–21.7%)) were positive for HBsAg. Table 2 depicts the percentage of people interviewed who displayed risk

factors and the prevalence of HBsAg among them. HBsAg positivity was highest among students living with a family (95.6%, n=65), followed by students having unprotected sex (65%, n=44), having multiple sexual partners (57%, n=39), living in a density of residence of more than two (50%, n=34), sharing sharp objects (46%, n=31) and having an HBsAg-positive mother (34%, n=23). Of the 107 (23%) students who had undergone a screening test for hepatitis B previously, 25 were HBsAg positive (37%) (Table 2).

Table 2: Prevalence of HBV risk factors

Characteristic	Overall n (%)	HBsAg positive n (%)	p-value
Knowledge of hepatitis B	248 (~~54~~54.26%)	38 (~~56~~ 55.9%)	0.8
History of blood transfusion	58 (~~13~~12.69%)	10 (~~15~~ 14.7%)	0.6
Multiple sexual partners	252 (~~55~~55.14%)	39 ~~(57~~ 57.4%)	0.7
History of hospital admission	108 (~~27~~26.63%)	16 (~~24~~23.5)	>0.9
HBsAg-positive mother	102 (~~22~~22.31%)	23 (~~34~~33.8%)	0.014
Family history of hepatitis B	71 (~~16~~ 15.53%)	15 (~~22~~ 22.05%)	0.3
Transcutaneous medical procedures	65 (~~14~~14.22%)	18 (26.5%)	0.002
History of drug injection	15 (3.3%)	1 (1.5%)	0.7
Risky practices (piercings, tattoos, etc.)	136 (~~30~~ 29.75%)	21 (30.88%)	0.8
Jail stay	28 (6.1%)	5 (7.4%)	0.6
Unprotected sex	297 (~~65~~ 64.98%)	44 (~~65~~64.7%)	>0.9
Sharing of sharp materials	186 (~~41~~40.70%)	31 (45.6%)	0.4
Housing style: family	353 (~~77~~ 77.24%)	65 (95.6 %)	0.004
Density of residence: >2	245 (~~54~~ 53.61%)	34 (50. 00 %)	0.5
History of hepatitis B testing	107 (~~23~~ 23.41%)	25 (~~37~~ 36.76%)	0.005*

Factors associated with HBV status in univariate and multivariate analyses

Logistic regression was used to test the association between HBV infection and the independent variables and to identify risk factors. Considering a p-value <0.30 in bivariate analysis, having an HBsAg-positive mother, a family history of HBV infection, a history of transcutaneous medical procedures, housing style and a history of HBV screening were

identified as candidate variables for multivariate analysis. Having an HBsAg-positive mother (AOR: 2.11), having a history of transcutaneous medical procedures (AOR: 2.97), and living with a family (AOR: 4.63) were significantly associated with HBV status, whereas age ≥26 years was a protective factor (AOR = 0.41) **(Table 3)**.

Table 3: Factors associated with HBV status of study participants in univariate and multivariate analysis.

Variable	Category	Overall n (%)	HBsAg prevalence	Univariable logistic regression		Multivariable logistic regression	
			n (%)	COR [95% CI]	P	AOR [95% CI])	P
Sex	Female	109 (~~24~~ 23.85)	16 (23.5)	1		1	
	Male	348 (~~76~~ 76.14)	52 (76.5)	1.11 (0.58-2.22)	0.761	-	
Age	≤ 25	343 (~~75~~ 75.05)	56 (82.4)	1		1	
	≥ 26	114 (~~25~~ 24.94)	12 (17.6)	0.39 (0.17-0.82)	0.018	0.41 (0.18-0.85)	0.022*
Marital status	Married	109 (~~24~~ 23.85)	17 (25.0)	1		1	
	Single	348 (~~76~~ 76.14)	51 (75.0)	0.58 (0.28-1.22)	0.145	0.65 (0.33-1.31)	0.219
History of surgical procedure	No	435 (~~95~~ 95.1)	68 (100.00)	1		1	
	Yes	22 (4.8)		0.00 (0.00-9467)	0.984	-	
History of blood transfusion	No	399 (~~87~~ 87.3)	58 (85.3)	1		1	
	Yes	58 (~~13~~ 12.69)	10 (14.7)	1.50 (0.64-3.29)	0.326	-	
Multiple sexual partners	>1	252 (~~55~~ 55.14)	39 (57.4)	1		1	
	1	205 (~~45~~ 44.85)	29 (42.6)	0.96 (0.53-1.73)	0.904	-	
Risky practices (piercings, tattoos, etc.)	No	321 (~~70~~ 70.24)	47 (69.1)	1		1	
	Yes	136 (30 29.75)	21 (30.9)	0.93 (0.48-1.74)	0.821	-	
History of hospital admission	No	349 (~~76~~ 76.36)	52 (76.5)	1		1	
	Yes	108 (~~24~~ 23.63)	16 (23.5)	1.06 (0.53-2.03)	0.869	-	
HBsAg-positive mother	No	355(~~78~~77.68)	45 (66.2)	1		1	

	Yes	102 (~~22~~ 22.31)	23 (33.8)	2.03 (1.09-3.74)	0.023	2.11 (1.16-3.78)	0.013*
History of drug injection	No	442 (96.7)	67 (98.5)	1		1	
	Yes	15 (3.3)	1 (1.5)	0.28 (0.01-1.65)	0.246	0.29 (0.02-1.59)	0.244
Jail stay	No	429 (~~94~~ 93.8)	63 (92.6)	1		1	
	Yes	28 (6.1)	5 (7.4)	0.85 (0.24-2.50)	0.785	-	
Unprotected sex	No	160(~~35~~35.01)	24 (35.3)	1		1	
	Yes	297 (~~65~~ 64.98)	44 (64.7)	0.95 (0.52-1.77)	0.869	-	
Transcutaneous medical procedures	No	392 (85.77)	50 (73.5)	1		1	
	Yes	65 ~~(14~~ 14.23)	18 (26.5)	3.14 (1.54-6.34)	0.001	2.97 (1.51-5.76)	0.001*
Sharing of sharp materials	No	271 (59 59.29)	37 (54.4)	1		1	
	Yes	186 (40.70)	31 (45.6)	1.33 (0.75-2.34)	0.327		
Housing style	Alone	48 (12.3)	3 (4.4)	1		1	
	Family	353 (~~77~~ 77.24)	65 (95.6)	4.54 (1.44-20.47)	0.022	4.63 (1.50-20.52)	0.018*
Density of residence	1-2	212 (~~46~~ 46.38)	34 (50.00)	1.49 (0.83-2.70)	0.186	1.45 (0.81-2.58)	0.210
	>2	245 (~~54~~ 53.61)	34 (50.00)	1		1	

95% CI = 95% Confidence Interval, COR: crude odds ratio, AOR: adjusted odds ratio, 1: reference category, *: significantly associated factors p-value <0.05

Discussion

In our study, we assessed the prevalence of HBV and its associated factors among students in the main public university and the main private university in N'Djamena, Chad. Our results show a prevalence of HBV infection (HBsAg positivity) of 14.87% (68/457). That finding places our study population in the high prevalence group, i.e., well above the 8% threshold established by the World Health Organization [29].

The HBsAg positivity prevalence found in our study is also higher than the overall prevalence of 12.4% reported for all age groups in Chad, and over those reported for some other population groups in N'Djamena, for example 7.2% in pregnant women [30] and 13.5% in HIV-infected patients [31]. Our higher prevalence may be due to the fact that our students were largely part of a relatively young—but not young enough—population. Indeed, with an average age of 24±3 years, our subjects most likely did not benefit from vaccination against HBV in childhood as part of the Expanded Program on Immunization introduced in 2008 [18]. Outside of that program, the relatively onerous price of the HBV vaccine is not covered by the current Chadian healthcare system, which acts as a barrier to large coverage in this population.

Moreover, the HBV prevalence found in our population is higher than those reported in studies on students from Togo (4.6%) [25], Cameroon (5.6%) [32] and Ethiopia (11.5%) [33]. It is lower however than that reported in Nigeria (31.5%) (34) and comparable to that reported in the Central African Republic (15.5%) [16].

Currently, Chadian students are not provided with prevention information (e.g. on sexuality, sexually transmitted diseases and risky practices), which most likely plays a part in that high prevalence rate. The environment in which students live may also carry risk factors (presence

of drugs, alcohol, sex, etc.) [15] and the students themselves are in a period of life where risk taking may be more common, as compared to other age groups. Furthermore, sex education continues to be a taboo subject in Chadian families.

Of the 107 students (23%) who had already been tested for HBV infection in the past, 25 (37%) tested positive in our study. That finding is consistent with data reported by Halatoko *et al* in Togo [26]. Our interpretation is that students who knew they were already infected might have been particularly tempted to participate in the study, hoping to receive treatment or to check their serological status.

Focusing on the management and clinical follow-up for HBsAg-positive students, and encouraging those who tested HBsAg and anti-HBc-antibody-negative to complete the vaccination course are both critical. All participants in our study received information on HBV infection and those who tested positive were referred to health facilities for follow-up.

Our data showed that maternal HBV status was significantly associated with HBsAg carriage among our students, likely explained by mother-to-child (vertical) transmission risks. Results similar to ours were reported in Gambia in 2007, where vertical transmission was responsible for 16% of chronic infections [35] and in Cameroon, where the risk of vertical transmission was substantial despite birth-dose vaccination [36]. These data confirm the hypothesis that in sub-Saharan Africa, a highly endemic region for HBV, vertical transmission merits as much attention as its horizontal counterpart [37].

Our study reported a significantly higher risk of transmission among students living in families, with a seroprevalence of 95.6% (65/68) in this study group. These results corroborate those obtained in the Central African Republic [16] , where intrafamilial transmission was reported to be associated with hepatitis B in students. This association is probably due to contact between HBV-infected family members [38], the exchange of sharp objects and the sharing of

personal-use items (those causing skin and mucous-membrane lesions), and a lack of knowledge on the different modes of transmission of HBV. For these reasons, public awareness and systematic screening of those living with infected people are essential.

A history of transcutaneous medical procedures was found to be a risk factor for hepatitis B in this study. This could be due to nosocomial transmission of HBV (resulting from non-compliance with universal recommendations to prevent postoperative infections and cross-contamination of equipment and medical devices) [39].

According to the multivariate analysis, students aged 26 years or more were less likely to be infected with HBV than those aged 25 years or less, suggesting age as a protective factor. Similar results were reported by Samsunder *et al* in South Africa [34], where participants aged 20–24 years were more exposed than other groups. It is possible that the older students had already been infected during childhood or adolescence but cleared that earlier HBV more or less quickly, and thus did not developed chronic infection. Unfortunately, we were unable to check for anti-HBc antibodies (Ab) to confirm this hypothesis. Similarly, we did not check for anti-HBs Ab, which could have given us an idea of the percentage of vaccinated students.

Strengths and limitations

Our study adds to data regarding the prevalence of hepatitis B virus infection in Chad. Such findings are expected to guide public health strategies ~~to diagnoste~~ for the diagnosis of HBV pathologies among students and the general population. Although our work involved a mixed population of students from both the University of N'djamena and Emi-Koussi University, it does have certain limitations. As underlined above, we were unable to carry out total anti-HBc Ab, anti-HBc IgM Ab and anti-HBs Ab testing. This would have allowed for a distinction between subjects who were acutely infected (with anti-HBc IgM Ab), chronic carriers, showing recovery from an acute or chronic infection (anti-HBc Ab positive) [40, 41], or immunized

against HBV (anti-HBs Ab positive). The face-to-face nature of our interviews may represent a limitation and constraints in the ~~The study is limited by using the interview s during face to face meting, limitations~~ study design may have introduced selection bias

Logistic regression was adjusted for other variables but only ten were taken into account. This may represent a limit on the control of confounding factors~~. and thus on the accuracy of our results~~

Conclusion and Recommendations

Our study showed that the prevalence of HBV infection in students was higher than the overall prevalence in Chad. Having an HBsAg-positive mother, a history of transcutaneous medical procedures, living with a roommate, and living with a family were significantly associated with HBV status. In view of our results, HBV prevention and treatment policies should include: awareness campaigns to inform at-risk populations like students on HBV transmission pathways; HBV vaccination at birth; mandatory full hepatitis vaccination status before children start school; the promotion of mass screening to identify & treat chronic HBV carriers and reduce transmission; reduction of vaccination costs; and the promotion of free-of-charge HBV screening for pregnant women.

List of abbreviations

Anti-HBs: Hepatitis B surface antibody, AOR: Adjusted odd ratio; CI: Confidence interval, COR: Crude odd ratio, HBsAg: Hepatitis B surface antigen, HBV: Hepatitis B virus.

Inclusivity in global research

Additional information regarding the ethical, cultural, and scientific considerations specific to inclusivity in global research is included in the Supporting Information (S2 Checklist).

Supporting information

S1 Checklist. Inclusivity in global research questionnaire. (DOCX)

S2 Data. (XLSX)

Acknowledgements

We thank the laboratory technicians at Hôpital de la Paix in N'djamena who took the blood samples and performed the various tests. We express our gratitude to the survey's data collectors and supervisors. We furthermore thank the deans of Cheikh Anta Diop University in Dakar for their guidance. We express our gratitude both to the student secretaries who facilitated participation in the study and particularly to the students who joined us for it.

Availability of data and materials

The datasets generated and/or analyzed during the current study are available in the Mendeley repository here .

Conflicts of interest

The authors have declared that no competing interests exist.

Funding

The authors received no specific funding for this work.

Author Contributions

Conceptualization: Nalda Debsikréo, Françoise Lunel-Fabiani

Data curation: Nalda Debsikréo, Birwé Léon Mankréo, Azoukalné Moukénet

Formal analysis: Nalda Debsikréo, Birwé Léon Mankréo, Azoukalné Moukénet

Funding acquisition: Nalda Debsikréo

Investigation: Nalda Debsikréo, Merwa Ouangkake, Nathan Mara, Ali Mahamat Moussa

Methodology: Nalda Debsikréo, Birwé Léon Mankréo, Azoukalné Moukénet, Ali Mahamat Moussa Ndèye Coumba Toure-Kane, Françoise Lunel-Fabiani

Project administration: Nalda Debsikréo, Merwa Ouangkake, Nathan Mara, Ali Mahamat Moussa

Software: Nalda Debsikréo, Birwé Léon Mankréo, Azoukalné Moukénet

Laboratory: Nalda Debsikréo, Merwa Ouangkake, Nathan Mara

Validation: Nalda Debsikréo; Birwé Léon Mankréo, Azoukalné Moukénet, Merwa Ouangkake, Nathan Mara, Ali Mahamat Moussa, Ndèye Coumba Toure-Kane, Françoise Lunel-Fabiani

Visualization: Nalda Debsikréo, Birwé Léon Mankréo, Azoukalné Moukénet

Writing – original draft: Nalda Debsikréo, Françoise Lunel-Fabiani

Writing – review & editing: Nalda Debsikréo, Birwé Léon Mankréo, Azoukalné Moukénet, Merwa Ouangkake, Nathan Mara, Ali Mahamat Moussa, Ndèye Coumba Toure-Kane, Françoise Lunel-Fabiani

References

1. Stanaway JD, Flaxman AD, Naghavi M, Fitzmaurice C, Vos T, Abubakar I, et al. The global burden of viral hepatitis from 1990 to 2013: findings from the Global Burden of Disease Study 2013. The Lancet. 10 sept 2016; 388(10049):1081‑8.

2. Selzer L, Zlotnick A. Assembly and Release of Hepatitis B Virus. Cold Spring Harb Perspect Med. déc 2015; 5(12):a021394.

3. Global hepatitis report, 2017 [Internet]. [[Cited 26 March 2023].]. https://www.who.int/publications-detail-redirect/9789241565455

4. Polaris Observatory Collaborators. Global prevalence, cascade of care, and prophylaxis coverage of hepatitis B in 2022: a modelling study. Lancet Gastroenterol Hepatol. oct 2023;8(10):879‑907.

5. Gnyawali B, Pusateri A, Nickerson A, Jalil S, Mumtaz K. Epidemiologic and socioeconomic factors impacting hepatitis B virus and related hepatocellular carcinoma. World J Gastroenterol. 7 août 2022; 28(29):3793‑802.

6. Spearman CW, Afihene M, Ally R, Apica B, Awuku Y, Cunha L, et al. Hepatitis B in sub-Saharan Africa: strategies to achieve the 2030 elimination targets. Lancet Gastroenterol Hepatol. 1 déc 2017; 2(12):900‑9.

7. Polaris Observatory Collaborators. Global prevalence, treatment, and prevention of hepatitis B virus infection in 2016: a modelling study. Lancet Gastroenterol Hepatol. juin 2018;3(6):383‑403.

8. Lu PJ, O'Halloran AC, Williams WW, Nelson NP. Hepatitis B vaccination coverage among adults aged ≥ 18 years traveling to a country of high or intermediate endemicity, United States, 2015. Vaccine. 25 avr 2018; 36(18):2471‑9.

9. Park Y, Lee JH, Sinn DH, Park JY, Kim MA, Kim YJ, et al. Risk and Risk Score Performance of Hepatocellular Carcinoma Development in Patients With Hepatitis B Surface Antigen Seroclearance. Clin Transl Gastroenterol. 11 janv 2021; 12(1):e00290.

10. WHO. WHO | Regional Office for Africa. [Cited 22 June 2022]. World Hepatitis Day, 28 July 2021. https://www.afro.who.int/regional-director/speeches-messages/world-hepatitis-day-28-july-2021

11. Abbas Z, Abbas M. Challenges in Formulation and Implementation of Hepatitis B Elimination Programs. Cureus. 13(4):e14657.

12. GBD 2019 Hepatitis B Collaborators. Global, regional, and national burden of hepatitis B, 1990-2019: a systematic analysis for the Global Burden of Disease Study 2019. Lancet Gastroenterol Hepatol. sept 2022;7(9):796‑829.

13. Zampino R, Boemio A, Sagnelli C, Alessio L, Adinolfi LE, Sagnelli E, et al. Hepatitis B virus burden in developing countries. World J Gastroenterol. 14 nov 2015;21(42):11941‑53.

14. Tao I, Compaoré TR, Diarra B, Djigma F, Zohoncon TM, Assih M, et al. Seroepidemiology of hepatitis B and C viruses in the general population of burkina faso. Hepat Res Treat. 2014; 2014:781843.

15. Gülsoy SS, Siyez E, Esen E, Baran B, Siyez DM. Predictors of self-reported sexually transmitted infections among Turkish university students. J Dtsch Dermatol Ges J Ger Soc Dermatol JDDG. juill 2023;21(7):751‑8.

16. Komas NP, Baï-Sepou S, Manirakiza A, Léal J, Béré A, Le Faou A. The prevalence of hepatitis B virus markers in a cohort of students in Bangui, Central African Republic. BMC Infect Dis. 29 juill 2010; 10:226.

17. Njoku OO. Prevalence of assymptomatic hepatitis B virus among sexually active youths in a rural community of Ebonyi state, Southeast Nigeria. Int J Infect Dis. 1 avr 2016; 45:450‑1.

18. Ferrinho P, Dramé M, Tumusiime P. Perceptions of the usefulness of external support to immunization coverage in Chad: an analysis of the GAVI-Alliance cash-based support. Pan Afr Med J. 7 juin 2013;15:44.

19. tcd_rpt_annuairesstatistiquessanitaires_ministeresante_2017.pdf [Internet]. [Cited 13 May 2022].

https://www.humanitarianresponse.info/sites/www.humanitarianresponse.info/files/documents/files/tcd_rpt_annuairesstatistiquessanitaires_ministeresante_2017.pdf

20. WATHI. Politique nationale de santé 2016-2030, ministère de la Santé publique du Tchad, 2016 [Internet]. WATHI. 2021 [cited 17 Feb 2023]. https://www.wathi.org/wathinote-election-tchad-situation-sanitaire/politique-nationale-de-sante-2016-2030-2016/

21. La formation à l'université de N'Djaména [Internet]. [Cited 23 april 2022]]. https://archive.wikiwix.com/cache/index2.php?url=https%3A%2F%2Fwww.universite-ndjamena.td%2Fformation#federation=archive.wikiwix.com

22. L'Université [Internet]. Unniversité Emikoussi. 2022 [cited 13 March 2023]. https://universite-emikoussi.net/luniversite/

23. Rajamoorthy Y, Taib NM, Munusamy S, Anwar S, Wagner AL, Mudatsir M, et al. Knowledge and awareness of hepatitis B among households in Malaysia: a community-based cross-sectional survey. BMC Public Health. 10 janv 2019; 19:47.

24. Mosaad M, Al Nozha OM, Yamany H, Amer S. A survey of hepatitis B immune status of Taibah University medical students. J Taibah Univ Med Sci. 1 déc 2014; 9(4):301‑6.

25. Ekouevi DK, Thomas A, Sewu D, Lawson-Ananissoh L, Tchounga B, Salou M, et al. Prevalence of Hepatitis B among Students from the University of Lomé, Togo in 2015. Open J Epidemiol. 8 août 2017; 7(3):262‑72.

26. Halatoko WA, Patassi A, Yanogo P, Banla LI, Koba A, Issa Z, et al. Risk factors of hepatitis B virus surface antigen carriage and serological profile of HBsAg carriers in Lomé Togo, 2016. BMC Public Health. 8 janv 2019; 19(1):32.

27. Njai HF, Shimakawa Y, Sanneh B, Ferguson L, Ndow G, Mendy M, et al. Validation of rapid point-of-care (POC) tests for detection of hepatitis B surface antigen in field and laboratory settings in the Gambia, Western Africa. J Clin Microbiol. avr 2015;53(4):1156‑63.

28. Ouoba S, Ko K, Lingani M, Nagashima S, Guingané AN, Bunthen E, et al. Intermediate hepatitis B virus infection prevalence among 1622 pregnant women in rural Burkina Faso and implications for mother-to-child transmission. Sci Rep. 14 avr 2023; 13(1):6115.

29. Guidelines for the prevention, care and treatment of persons with chronic hepatitis B infection [Internet]. [cited 13 jan 2024]. https://www.who.int/publications-detail-redirect/9789241549059

30. Debsikréo N, Mankréo BL, Moukenet A, Ndiaye AJS, Diouf NL, Lo G, et al. Prevalence of Hepatitis B and Associated Factors among Pregnant Women in N'djamena, Chad. Fortune J Health Sci. 13 juill 2023; 6(3):253‑62.

31. Bessimbaye N, Moussa AM, Mbanga D, Tidjani A, Mahamat SO, Ngawara MN, et al. [Seroprevalence of HBs Ag and of anti-HCV antibodies among HIV infected people in N'Djamena, Chad]. Bull Soc Pathol Exot 1990. déc 2014;107(5):327‑31.

32. servais albert fiacre E. Statut Vaccinal Contre le Virus de l'Hépatite Virale B et portage de l'Antigène Hbs chez les Etudiants en Médecine et en Pharmacie de l'Université de Douala au Cameroun. Health Sci Dis. 1 janv 2014;

33. Tesfa T, Hawulte B, Tolera A, Abate D. Hepatitis B virus infection and associated risk factors among medical students in eastern Ethiopia. PLOS ONE. 19 févr 2021; 16(2):e0247267.

34. Tula MY, Iyoha O. A Cross-sectional Study on the Sero-prevalence of Hepatitis B Surface Antigen (HBsAg) among Apparently Healthy Students of a Tertiary Institution in North-Eastern Nigeria. Int J Trop Dis Health. 10 janv 2015; 7:102‑8.

35. Shimakawa Y, Lemoine M, Njai HF, Bottomley C, Ndow G, Goldin RD, et al. Natural history of chronic HBV infection in West Africa: a longitudinal population-based study from The Gambia. Gut. 1 déc 2016;65(12):2007‑16.

36. Shimakawa Y, Veillon P, Birguel J, Pivert A, Sauvage V, Guillou-Guillemette HL, et al. Residual risk of mother-to-child transmission of hepatitis B virus infection despite timely birth-dose vaccination in Cameroon (ANRS 12303): a single-centre, longitudinal observational study. Lancet Glob Health. 1 avr 2022; 10(4):e521‑9.

37. Shimakawa Y, Toure-Kane C, Mendy M, Thursz M, Lemoine M. Mother-to-child transmission of hepatitis B in sub-Saharan Africa. Lancet Infect Dis. 1 janv 2016;16(1):19‑20.

38. Mast EE, Weinbaum CM, Fiore AE, Alter MJ, Bell BP, Finelli L, et al. A comprehensive immunization strategy to eliminate transmission of hepatitis B virus infection in the United States: recommendations of the Advisory Committee on Immunization Practices (ACIP) Part II: immunization of adults. MMWR Recomm Rep Morb Mortal Wkly Rep Recomm Rep. 8 déc 2006; 55(RR-16):1‑33; quiz CE1-4.

39. Sugimoto S, Nagakubo S, Ito T, Tsunoda Y, Imamura S, Tamura T, et al. A case of acute hepatitis B related to previous gynecological surgery in Japan. J Infect Chemother Off J Jpn Soc Chemother. juin 2013;19(3):524‑9.

40. Lau GKK. How do we handle the anti-HBc positive patient? (In highly endemic settings). Clin Liver Dis. 27 mars 2015; 5(2):29‑31.

41. Gachara G, Magoro T, Mavhandu L, Lum E, Kimbi HK, Ndip RN, et al. Characterization of occult hepatitis B virus infection among HIV positive patients in Cameroon. AIDS Res Ther. 8 mars 2017; 14:11.

Supporting information

S1 File. Questionnaire (docx)

381 S2 File. Checklist (docx)

472

Response to reviewers

One of the reviewers suggested that final version should incorporate the following changes based on the manuscript evaluation:

"In the submitted manuscript “Prevalence of hepatitis B virus infection and its associated factors among students in N’Djamena, Chad”, PONE-D-22-22361R2, this study addressed the high prevalence of HBV infection among university students in Chad. HBV prevention, especially HBV immunization, should be considered in low-income countries.

However, there are some points that need to be clarified:

1. The authors should mention inclusion criteria in the method part (e.g., sex, age, health status, etc.).

Thank you for this observation. Students who accepted to participate in the study were enrolled consecutively irrespective of age, sex and other sociodemographic characteristics or risk factors line 105-109

2. The data was collected using a questionnaire, was it a self-administered questionnaire or face-to-face interview? Please specify in data collection method.

Thank you for your pertinent observation. The data was it questionnaire face-to-face line 122-123

3. The prevalence of HBV infection was 14.87%, what’s about 95%CI? Please specify.

14.87 is the prevalence of HBsAg in the population studied (95% CI = 13.9-21.7%). Line 193

4. I would suggest using median age instead of mean age.

Thank you for this suggestion. Median age was 24 years. Line 185.

5. Considering $p<0.30$ in bivariate analysis, why did the authors select only five variables for multivariable logistic regression? (What about age, marital status, knowledge of HBV, history of drug injection, density of residence? there were also $p<0.30$.)

Age was included in the analysis (age>26 was a protective factor). A previous reviewer and the editor suggested, with reason, to remove knowledge of HBV, which of course is a confounding factor. We have forgotten to remove it from the manuscript in the data analysis paragraph. Marital status, history of drug injection and density of residence were also included in the analysis, but were not significantly associated with HBs Ag positivity (see table 3). Line 215

6. The authors did not ask about the history of HB immunization in the study participants. Although most likely did not benefit from vaccination against HBV in childhood as part of EPI, some may receive the vaccine in adult. We therefore can distinguish between who has protective or susceptible for HBV infection."

Thank you for your comment. Unfortunately, students were not asked about their vaccination status. Please see discussion — line 275—277

I commend the authors did a great job by incorporating reviewers' comments which substantively improved the paper. However, the paper doesn't until now rigorously align with Plos One's authors guidelines. Please note the following:

1. References: Plos One uses brackets;

Thank you for this observation, we've changed hooks

2. In all Tables, add 1 decimal for percentages

Thank you for your comment, we have added 1 decimal for percentages. Table 1: line 188-189, Table 2:203-204 Table 3:214-215

3. When citing R, the date is unnecessary

Thank you, we have deleted. Line 171

Additionally, reviewer #2 has raised some comments to be integrated. I suggest to put these questions in the discussion section or alternatively, add a study limitations section.

Thank you for this suggestion, we have added a section on the Strengths and limitations

I commend the authors did a great job by incorporating reviewers' comments which substantively improved the paper. However, the paper doesn't until now rigorously align with Plos One's authors guidelines. Please note the following:

1. References: Plos One uses brackets;

Thank you for this observation, we've changed hooks

2. In all Tables, add 1 decimal for percentages

Thank you for your comment, we have added 1 decimal for percentages. Table 1: line 188-189, Table 2:203-204 Table 3:214-215

3. When citing R, the date is unnecessary

Thank you, we have deleted. Line 171

Additionally, reviewer #2 has raised some comments to be integrated. I suggest to put these questions in the discussion section or alternatively, add a study limitations section.

Thank you for this suggestion, we have added a section on the Strengths and limitations

However, there are some points that need to be clarified:

1. the authors should mention inclusion criteria in the method part (e.g., sex, age, health status, etc

Thank you for this observation. We have revised the text accordingly, stating that students who accepted to participate in the study were enrolled consecutively irrespective of age, sex, other sociodemographic characteristics or risk factors. Line 105‐109.

2. The data was collected using a questionnaire, was it a self-administered questionnaire or face-to-face interview? Please specify in data collection method.

Thank you for your pertinent observation. The interviews were done during face-to-face meetings. Line 122–123.

3. The prevalence of HBV infection was 14.87%, what’s about 95%CI? Please specify.

14.87 is the prevalence of HBsAg in the population studied (95% CI = 13.9‐21.7%). This confidence interval was added to the text. Line 195.

4. I would suggest using median age instead of mean age.

Thank you for this suggestion. Median age was 24 years. Line 185.

5. Considering $p<0.30$ in bivariate analysis, why did the authors select only five variables for multivariable logistic regression? (What about age, marital status, knowledge of HBV, history of drug injection, density of residence? there were also $p<0.30$.)

Age was included in the analysis (age ≥26 was a protective factor). A previous reviewer and the editor suggested, with reason, to remove knowledge of HBV, which of course is a confounding factor. We had forgotten to remove it from the manuscript in the data analysis paragraph. Marital status, history of drug injection and density of residence were also included in the analysis, but were not significantly associated with HBs Ag positivity (see Table 3).

6. The authors did not ask about the history of HB immunization in the study participants. Although most likely did not benefit from vaccination against HBV in childhood as part of EPI, some may receive the vaccine in adult. We therefore can distinguish between who has protective or susceptible for HBV infection.

Thank you for your comment. Unfortunately, students were not asked about their vaccination status. We've touched upon this point in the discussion. Line 274–275
