# Supplementary material for: Prevalence of hepatitis B virus infection and its associated factors among students in N’Djamena, Chad
Source: PLoS One. 2024 Apr 18;19(4):e0273589. doi: 10.1371/journal.pone.0273589 (PMC11025733; doi:10.1371/journal.pone.0273589)
Supplement: Attachment — Submitted filename: PONE-D-22-22361_R1_reviewer.docx [file pone.0273589.s008.docx]

**Title:**	Prevalence and associated factors of hepatitis B virus infection among students in N'Djamena, Chad.

**Manuscript ID:** PONE-D-22-22361R1

This paper addressed an interesting topic which is a global public health issue, particularly in low income countries. So, I would like to acknowledge the authors for their effort to come up with this relevant topic. However this paper need a through edition and clarification to be an additional evidence for the scientific community and the general public health improvement.

General comments:

- Overall, the paper lacks standard manuscript quality. This paper need a lot of work for better improvement.
- There are lots of vague words and sentences (with no meaning)
- There are lots of grammar errors.
- It need lots of language edition
- The authors need to clarify lots of issues

| **Line Number** | **Comment** | **Remark** |
| --- | --- | --- |
| **Title** | | |
| **Line N^o^: 1-2** | - Prevalence and associated factors of hepatitis B virus infection among students in N'Djamena, Chad. - **I think it may be easy if you write it as:** “Prevalence of hepatitis B virus infection and its associated factors among students in N'Djamena, Chad. - ***Check this with your objective on line 24 & 25*** |  |
| **Abstract** | | |
| **General comment** | - I hope you will write the abstract on an independent page |  |
| **Line N^o^: 24-25** | - This study aimed to determine the prevalence of HBV and identify factors among students in N'Djamena, Chad. - The highlighted sentence does not give meaning - Who are these students? Medical students or any students? High school vs. elementary; higher institution vs. private institutions, please specify your study units - Do you mean identify factors associated with it? - We mostly state our study population in our objective |  |
| **Line N^o^: 28-29** | What type of sampling is “a consecutive sampling”?  Please state the sampling methods you used.  Did you took all the study units or you used systematic sampling? But you stated in our method as “students who volunteered to participate”  You can also state you final sample with this statement |  |
| **Line N^o^: 32** | Did you really do multivariate logistic regressions or you did **multivariable logistic regressions**  **Differentiate between the two** |  |
| **Line N^o^: 32-34** | Re-write this sentence: “Descriptive analysis, binary and multivariate logistic regressions were used to examine the relationship between dependent variables and socio-demographic factors including other associated factors”  You need to mention all other associated factors or you better name all as independent variables. |  |
| **Line N^o^: 36-37** | The prevalence of HBV infection was 14.87% (95% CI =13,9–21,7%).  What is the purpose of mentioning CI here?  When do we use CI?  Aren’t you sure it is 14.87%?  3,9–21,7%: is it a comma or full stop between the numbers? |  |
| **Line N^o^: 39-40** | Be consistent when using hepatitis B. Is it hepatitis B, hepatitis B virus, HBV? |  |
| **Line N^o^: 41-42** | “Being in a roommate” what does this mean? How can this be related to scientific logic? |  |
| **Line N^o^: 42-44** | - How did “having history of HBV screening (AOR: 2.03, 95%CI: 1.11-3.65, p=0.019) was associated with a higher risk of HBV infection. - So what is your recommendation on this? Do you recommend not to be screened for HBV? |  |
| **Line N^o^: 46-47** | - **I don’t think you wrote a good conclusion** - How can you conclude this small study (conducted in two university) to the whole country? It is also study among small students and you generalized it as endemic in the whole Chad - You also included students who volunteered to participate (non-probability sampling with the highest bias) - Please re-write your conclusion based on you finding and write its implication |  |
| **Line N^o^: 49-51** | - **Don’t you think this sentence contradict you result above?** - Policies, such as routine screening of the population, birth dose vaccine and access to treatment are needed to reduce the incidence and mortality of HBV related diseases. |  |
| **Line N^o^: 52** | Re-check your key words **(hepatitis B, some words are written in italic)** |  |
| **Introduction** | | |
| **General comment** | There are a lot of grammar errors in this section. Please consult someone who can help you with language edition |  |
| **Line N^o^: 55-56** | Hepatitis B virus (HBV) is a DNA. Does this give any sense to you?  Please consult someone who help you with grammar |  |
| **Line N^o^: 58-60** | Re-write this sentence: state the data reference (global vs. specific geographic area)  Chronic HBV infection is widespread, and 900,000 deaths per year are related to HBV infection (3) and its prevalence is 3.6% (4). |  |
| **Line N^o^: 58-60** | Where is your reference for this sentence:  “Despite a lack of accurate data on viral hepatitis B in Africa, it is estimated that its prevalence is one of the highest in the world” |  |
| **Line 79** | What do you mean by “HIV population of N'Djamena”?  ***Are there a people called HIV population in Chad?***  **Do you mean HIV infected people?** |  |
| **Line 83-84** | What is your reference for this sentence: “HBV can be transmitted from biological fluids of infected individuals, especially blood and sexual secretions. |  |
| **Line 88** | Merge the two references in the same parenthesis  “unprotected sexual intercourse (3) (15).” |  |
| **Methods and materials** | | |
| **General comment** | I would recommend you to re-write “Materials and Methods” as “Methods and Materials” |  |
| **Line 109** | “Study design and framework”  What do you mean by framework? |  |
| **Line 123** | What type of sampling is “consecutive sampling” in volunteer sampling? |  |
| **Data collection** | What was the source of your data collection tool? Is it standardized? If so, what was the source of you data collection tool?  Who were the data collectors? |  |
| **Socio-demographic data collection** | The heading and what you mentioned in the subsequent paragraph does not match |  |
|  | **Line 152:** Is medical/non-medical history a socio-demographic data or clinical data? |  |
| **Laboratory data processing** | I recommend you write this heading as specimen collection and processing |  |
| **Line 159-160** | Use reference manager to cite your reference in this sentence  “This is a qualitative test for the rapid detection of HBsAg, with very good sensitivity and specificity (100%) [20).” |  |
| **Line 171-172** | “relationship between dependent variables and socio-demographic factors including other risk factors  Simply write as association between outcome variable and independent/covariates variables. |  |
| **Data analysis** | Your data analysis section is not well organized and bulky. There are lots of redundancy. It is almost one page. Please summarize your **Data analysis section** |  |
| **Line 172-173** | What do you mean by “Quantitative variables”?  Do you have qualitative variables?  Isn’t Categorical variables a quantitative variables  Quantitative variables can be categorized in to continuous/numerical vs. categorical |  |
|  | When do we use Mann–Whitney U test?  Dd you had any non-parametric data? |  |
| **Line 177** | “In both cases, the level of significance was set at p < 5%”.  Please use the standard value which is < 0.05”. |  |
| **Ethical consideration** | “written informed consent was obtained from all participants for inclusion in the study.  Have they also signed it?  If so, write it as written, informed and signed consent |  |
| **Results** | | |
| **Line 209** | “Age ranged from 19 to 59 years” does this give you any sense? |  |
| **Table 1** | What is your based for age classification?  Is it the standard classification? If so, what is your standard reference? |  |
| **Line 233** | Where is table 2  You also cited table 2 in two different sections? Check line 233 and line 243 |  |
|  | Tables should come immediately following the sentence or paragraph |  |
|  | You included tables in the main document for some heading and not for others. Why? |  |
| **Line 244-254** | I don’t think you are expected to mention AOR with CI both in the paragraph and the table. You can just mention the AOR in the paragraphs. |  |
| **Table 3** | You need to edit table three. (Merge lines, minimize horizontal and vertical lines).  Just use borders for bottom and top of the table and between the first and second rows. |  |
|  | Be consistent in preparing tables and figures  Follow the journal standard. |  |
| **Regression table** | How did only five variables selected for multivariable logistic regression?  You should have increased the cut of value for binary logistic regression to 0.03 or more to fit your model |  |
|  | You also need to include frequency of each observation (cell) so that we can prove you model |  |
|  | Your regression table is not satisfactory |  |
| **Discussion** | | |
| **Line 269-271** | Doe this sentence give you any meaning  We found, in a population of 457 students from the University of N'Djamena and the University of Emi Koussi, Chad, a prevalence of HBV infection (HBsAg positivity) of 14.87% (95%CI: 13.9%, 21.7%). |  |
|  | In general you need to improve your discussion in line with your primary outcome and regression analysis including comparison with other findings and their implications to the general public health. |  |
| **Strength and limitation of your study** | You included only five variables for logistic regression. There seems a lot of confounders you didn’t control for. How can this affect your finding? |  |
| **Conclusion** | Re-write your finding  You need to summarize your findings with its implication and recommendations |  |
