# Supplementary material for: Prevalence of hepatitis B virus infection and its associated factors among students in N’Djamena, Chad
Source: PLoS One. 2024 Apr 18;19(4):e0273589. doi: 10.1371/journal.pone.0273589 (PMC11025733; doi:10.1371/journal.pone.0273589)
Supplement: Attachment — Submitted filename: PONE-D-22-22361_Rep reviewer R2.docx [file pone.0273589.s010.docx]

**Title:**	Prevalence and associated factors of hepatitis B virus infection among students in N'Djamena, Chad.

**Manuscript ID:** PONE-D-22-22361R1

This paper addressed an interesting topic which is a global public health issue, particularly in low income countries. So, I would like to acknowledge the authors for their effort to come up with this relevant topic. However this paper need a through edition and clarification to be an additional evidence for the scientific community and the general public health improvement.

General comments:

- Overall, the paper lacks standard manuscript quality. This paper need a lot of work for better improvement.
- There are lots of vague words and sentences (with no meaning)
- There are lots of grammar errors.
- It need lots of language edition
- The authors need to clarify lots of issues

| **Line Number** | **Comment** | **Remark** |
| --- | --- | --- |
| **Title** | | |
| **Line N^o^: 1-2** | - Prevalence and associated factors of hepatitis B virus infection among students in N'Djamena, Chad. - **I think it may be easy if you write it as:** “Prevalence of hepatitis B virus infection and its associated factors among students in N'Djamena, Chad. - ***Check this with your objective on line 24 & 25*** | Thank you for this suggestion, which we have retained for the revised manuscript. |
| **Abstract** | | |
| **General comment** | - I hope you will write the abstract on an independent page | We've put the summary on a separate page. |
| **Line N^o^: 24-25** | - This study aimed to determine the prevalence of HBV and identify factors associated with HBV infection among students in N'Djamena, Chad. - The highlighted sentence does not give meaning - Who are these students? Medical students or any students? High school vs. elementary; higher institution vs. private institutions, please specify your study units - Do you mean identify factors associated with it? - We mostly state our study population in our objective | Thank you for your comment.  We have added a more thorough description of the students included in the study.  See Methods and Materials  Sample size from different faculties (…..list) |
| **Line N^o^: 28-29** | What type of sampling is “a consecutive sampling”?  Please state the sampling methods you used.  Did you took all the study units or you used systematic sampling? But you stated in our method as “students who volunteered to participate”  You can also state you final sample with this statement | Thank you for your comment.  We have changed this wording to “were included in the study consecutively.” |
| **Line N^o^: 32** | Did you really do multivariate logistic regressions or you did **multivariable logistic regressions**  **Differentiate between the two** | Thank you for this observation.  Indeed, we performed a multivariate logistic regression.  The word “multivariable” snuck in under the influence of French. |
| **Line N^o^: 32-34** | Re-write this sentence: “Descriptive analysis, binary and multivariate logistic regressions were used to examine the relationship between dependent variables and socio-demographic factors including other associated factors”  You need to mention all other associated factors or you better name all as independent variables. | Thank you for this advice. The manuscript has been rewritten to address these concerns. |
| **Line N^o^: 36-37** | The prevalence of HBV infection was 14.87% (95% CI =13,9–21,7%).  What is the purpose of mentioning CI here?  When do we use CI?  Aren’t you sure it is 14.87%?  3,9–21,7%: is it a comma or full stop between the numbers? | Thank you, yes, it's 14.87%, and we’ve changed the comma decimals into point decimals. |
| **Line N^o^: 39-40** | Be consistent when using hepatitis B. Is it hepatitis B, hepatitis B virus, HBV? | Thank you for this advice. Although specific wording can be context-specific, we’ve harmonized the text around “hepatitis B virus / HBV” to describe the virus and “hepatitis B” to describe the disease. |
| **Line N^o^: 41-42** | “Being in a roommate” what does this mean? How can this be related to scientific logic? | Thank you. The manuscript has been extensively rewritten. It is now clear that we are speaking of several students sharing a habitation. |
| **Line N^o^: 42-44** | - How did “having history of HBV screening (AOR: 2.03, 95%CI: 1.11-3.65, p=0.019) was associated with a higher risk of HBV infection. - So what is your recommendation on this? Do you recommend not to be screened for HBV? | Thank you for your pertinent observation.  In the multivariate analysis, the variable "history of HBV screening" was removed. |
| **Line N^o^: 46-47** | - **I don’t think you wrote a good conclusion** - How can you conclude this small study (conducted in two university) to the whole country? It is also study among small students and you generalized it as endemic in the whole Chad - You also included students who volunteered to participate (non-probability sampling with the highest bias) - Please re-write your conclusion based on you finding and write its implication | Thank you for your comment.  The University of N'Djamena, founded in 1971 as the "University of Chad," is the main component of higher education in the country.  It is also the largest public university in Chad, with 16,142 students spread over 4 campuses and 7 faculties (20). As for Emi Koussi University, it is one of the largest private universities, with around 3,000 students and 2 faculties on 2 campuses (20).  Our population was composed not of volunteers but of students who accepted to participate in the study. We’ve reworded where necessary to make our conclusions more coherent with the work done. |
| **Line N^o^: 49-51** | - **Don’t you think this sentence contradict you result above?** - Policies, such as routine screening of the population, birth dose vaccine and access to treatment are needed to reduce the incidence and mortality of HBV related diseases. | Thank you for this observation. The conclusion has been rewritten. |
| **Line N^o^: 52** | Re-check your key words **(hepatitis B, some words are written in italic)** | Thank you for your comment.  We have cleaned up how the keywords are presented. |
| **Introduction** | | |
| **General comment** | There are a lot of grammar errors in this section. Please consult someone who can help you with language edition | We had the manuscript proofread by a native-English-speaking, formally trained, experienced, professional medical translator/proofreader. He indeed made a great number of changes to correct grammatical errors and improve the readability of the text. We hope that you will find the changes satisfactory, but remain open to any additional specific suggestions you may have. |
| **Line N^o^: 55-56** | Hepatitis B virus (HBV) is a DNA. Does this give any sense to you?  Please consult someone who help you with grammar | This has been done, see the response to the preceding question. |
| **Line N^o^: 58-60** | Re-write this sentence: state the data reference (global vs. specific geographic area)  Chronic HBV infection is widespread, and 900,000 deaths per year are related to HBV infection (3) and its prevalence is 3.6% (4). | We have rewritten this sentence and added the data reference. |
| **Line N^o^: 58-60** | Where is your reference for this sentence:  “Despite a lack of accurate data on viral hepatitis B in Africa, it is estimated that its prevalence is one of the highest in the world” | The reference has been added to the revised manuscript. |
| **Line 79** | What do you mean by “HIV population of N'Djamena”?  ***Are there a people called HIV population in Chad?***  **Do you mean HIV infected people?** | Thank you for your remark.  We have changed this wording to “HIV-infected patients.” |
| **Line 83-84** | What is your reference for this sentence: “HBV can be transmitted from biological fluids of infected individuals, especially blood and sexual secretions. | We have added the reference. |
| **Line 88** | Merge the two references in the same parenthesis  “unprotected sexual intercourse (3) (15).” | We’ve merged the two references as requested. |
| **Methods and materials** | | |
| **General comment** | I would recommend you to re-write “Materials and Methods” as “Methods and Materials” | We’ve made this change as requested. |
| **Line 109** | “Study design and framework”  What do you mean by framework? | Thank you, we have rewritten this section title as “Study design.” |
| **Line 123** | What type of sampling is “consecutive sampling” in volunteer sampling? | Thank you for this observation. we have changed the wording here to “students who accepted to participate in the study were enrolled consecutively and had blood specimens drawn.” |
| **Data collection** | What was the source of your data collection tool? Is it standardized? If so, what was the source of you data collection tool?  Who were the data collectors? | Thank you for your remark. Data were collected by two laboratory technicians. Thereto, we used a self-developed data collection sheet based on Rajamoorthy Y et al (21). |
| **Socio-demographic data collection** | The heading and what you mentioned in the subsequent paragraph does not match | Thank you for your remark. We have changed this to “Study variables” in the revised manuscript. |
|  | **Line 152:** Is medical/non-medical history a socio-demographic data or clinical data? |  |
| **Laboratory data processing** | I recommend you write this heading as specimen collection and processing | Thank you for your suggestion, which we have implemented. |
| **Line 159-160** | Use reference manager to cite your reference in this sentence  “This is a qualitative test for the rapid detection of HBsAg, with very good sensitivity and specificity (100%) [20).” | Thank you and we have made this change. |
| **Line 171-172** | “relationship between dependent variables and socio-demographic factors including other risk factors  Simply write as association between outcome variable and independent/covariates variables. | Thank you. We have changed this to “an association between the outcome variable and independent/covariate variables,” as suggested. |
| **Data analysis** | Your data analysis section is not well organized and bulky. There are lots of redundancy. It is almost one page. Please summarize your **Data analysis section** | Thank you for your remark.  The data analysis section has been better streamlined in the revised manuscript. |
| **Line 172-173** | What do you mean by “Quantitative variables”?  Do you have qualitative variables?  Isn’t Categorical variables a quantitative variables  Quantitative variables can be categorized in to continuous/numerical vs. categorical | Quantitative variables are those that can be measured on a continuous scale. Qualitative variables, also known as categorical variables, differ from quantitative variables.  Quantitative variables are those with measurable numerical values. Quantitative variables and qualitative, or categorical, variables are distinct concepts. As an illustration, we transformed the quantitative variable 'age' into a categorical variable through discretization. |
|  | When do we use Mann–Whitney U test?  Dd you had any non-parametric data? | The Mann-Whitney U test (also known as the Mann-Whitney-Wilcoxon (MWW/MWU), Wilcoxon rank-sum test, or Wilcoxon-Mann-Whitney test) is a non-parametric statistical analysis.  The Mann-Whitney U test was conducted instead of parametric tests when the latter were not feasible. |
| **Line 177** | “In both cases, the level of significance was set at p < 5%”.  Please use the standard value which is < 0.05”. | Thank you for noticing this oversight. We have changed to “< 0.05” in the revised manuscript. |
| **Ethical consideration** | “written informed consent was obtained from all participants for inclusion in the study.  Have they also signed it?  If so, write it as written, informed and signed consent | Thank you, we have implemented the suggested wording. |
| **Results** | | |
| **Line 209** | “Age ranged from 19 to 59 years” does this give you any sense? | The range of age has a sense in that there are 59-year-old civil servants returning to university in the population. However, in the revised manuscript we have changed the wording to “Mean age: 24 ±3 years.” |
| **Table 1** | What is your based for age classification?  Is it the standard classification? If so, what is your standard reference? | There is no basis for age classification, nor is there a standard classification. We have rescaled age into two groups. |
| **Line 233** | Where is table 2  You also cited table 2 in two different sections? Check line 233 and line 243 | Thank you and we have corrected/verified this points. |
|  | Tables should come immediately following the sentence or paragraph | Thank you, we have modified table placement. |
|  | You included tables in the main document for some heading and not for others. Why? | We’ve reformatted to harmonized table presentation. |
| **Line 244-254** | I don’t think you are expected to mention AOR with CI both in the paragraph and the table. You can just mention the AOR in the paragraphs. | Thank you and we have implemented this suggestion. |
| **Table 3** | You need to edit table three. (Merge lines, minimize horizontal and vertical lines).  Just use borders for bottom and top of the table and between the first and second rows. | Thank you, we have changed the table’s formatting |
|  | Be consistent in preparing tables and figures  Follow the journal standard. | Thank you, we have modified the tables so that they meet the journal’s standards. |
| **Regression table** | How did only five variables selected for multivariable logistic regression?  You should have increased the cut of value for binary logistic regression to 0.03 or more to fit your model | To select the variables, we deployed a stepwise forward selection procedure using the AIC criterion. The procedure retained the five variables.  However, in the revised manuscript, we deployed an additional step, including all variables with a p-value of less than 0.3 from the univariate logistic regression in the final multivariate logistic regression model. |
|  | You also need to include frequency of each observation (cell) so that we can prove you model | Thank you. We have implemented this request. |
|  | Your regression table is not satisfactory | The table has been modified as necessary. **Line 201** |
| **Discussion** | | |
| **Line 269-271** | Doe this sentence give you any meaning  We found, in a population of 457 students from the University of N'Djamena and the University of Emi Koussi, Chad, a prevalence of HBV infection (HBsAg positivity) of 14.87% (95%CI: 13.9%, 21.7%). | Thank you. This sentence, along with the rest of the manuscript, has been largely modified to improve clarity for the reader. |
|  | In general you need to improve your discussion in line with your primary outcome and regression analysis including comparison with other findings and their implications to the general public health. | Thank you for this observation and we have modified the discussion as suggested. |
| **Strength and limitation of your study** | You included only five variables for logistic regression. There seems a lot of confounders you didn’t control for. How can this affect your finding? | Thank you. we have added this observation to the discussion on the limits of the study. |
| **Conclusion** | Re-write your finding  You need to summarize your findings with its implication and recommendations : | Thank you. Here too we have tried to better summarize our findings and their implications. |
