# Supplementary material for: Prevalence of hepatitis B virus infection and its associated factors among students in N’Djamena, Chad
Source: PLoS One. 2024 Apr 18;19(4):e0273589. doi: 10.1371/journal.pone.0273589 (PMC11025733; doi:10.1371/journal.pone.0273589)
Supplement: Attachment — Submitted filename: Response to reviewer.docx [file pone.0273589.s011.docx]

**Comments to the Author**

1. If the authors have adequately addressed your comments raised in a previous round of review and you feel that this manuscript is now acceptable for publication, you may indicate that here to bypass the “Comments to the Author” section, enter your conflict of interest statement in the “Confidential to Editor” section, and submit your "Accept" recommendation.

Reviewer #6: All comments have been addressed

Reviewer #7: All comments have been addressed

2. Is the manuscript technically sound, and do the data support the conclusions?

The manuscript must describe a technically sound piece of scientific research with data that supports the conclusions. Experiments must have been conducted rigorously, with appropriate controls, replication, and sample sizes. The conclusions must be drawn appropriately based on the data presented.

Reviewer #6: Yes

Reviewer #7: Yes

3. Has the statistical analysis been performed appropriately and rigorously?

Reviewer #6: Yes

Reviewer #7: Yes

4. Have the authors made all data underlying the findings in their manuscript fully available?

The [PLOS Data policy](http://www.plosone.org/static/policies.action#sharing) requires authors to make all data underlying the findings described in their manuscript fully available without restriction, with rare exception (please refer to the Data Availability Statement in the manuscript PDF file). The data should be provided as part of the manuscript or its supporting information, or deposited to a public repository. For example, in addition to summary statistics, the data points behind means, medians and variance measures should be available. If there are restrictions on publicly sharing data—e.g. participant privacy or use of data from a third party—those must be specified.

Reviewer #6: Yes

Reviewer #7: Yes

5. Is the manuscript presented in an intelligible fashion and written in standard English?

PLOS ONE does not copyedit accepted manuscripts, so the language in submitted articles must be clear, correct, and unambiguous. Any typographical or grammatical errors should be corrected at revision, so please note any specific errors here.

Reviewer #6: Yes

Reviewer #7: Yes

6. Review Comments to the Author

Please use the space provided to explain your answers to the questions above. You may also include additional comments for the author, including concerns about dual publication, research ethics, or publication ethics. (Please upload your review as an attachment if it exceeds 20,000 characters)

Reviewer #6: I congratulate the authors for doing a thorough revision, and the responses are satisfactory. I recommend this version be accepted for publication after editorial checks and formatting.

Reviewer #7: In the submitted manuscript “Prevalence of hepatitis B virus infection and its associated factors among students in N’Djamena, Chad”, PONE-D-22-22361R2, this study addressed the high prevalence of HBV infection among university students in Chad. HBV prevention, especially HBV immunization, should be considered in low-income countries.

However, there are some points that need to be clarified:

1. the authors should mention inclusion criteria in the method part (e.g., sex, age, health status, etc

Thank you for this observation. We have revised the text accordingly, stating that students who accepted to participate in the study were enrolled consecutively irrespective of age, sex, other sociodemographic characteristics or risk factors. Line 105–108.

2. The data was collected using a questionnaire, was it a self-administered questionnaire or face-to-face interview? Please specify in data collection method.

Thank you for your pertinent observation. The interviews were done during face-to-face meetings. Line 119–121.

3. The prevalence of HBV infection was 14.87%, what’s about 95%CI? Please specify.

14.87 is the prevalence of HBsAg in the population studied (95% CI = 13.9–21.7%). This confidence interval was added to the text. Line 191.

4. I would suggest using median age instead of mean age.

Thank you for this suggestion. Median age was 24 years. Line 182.

5. Considering p<0.30 in bivariate analysis, why did the authors select only five variables for multivariable logistic regression? (What about age, marital status, knowledge of HBV, history of drug injection, density of residence? there were also p<0.30.)

Age was included in the analysis (age ≥26 was a protective factor). A previous reviewer and the editor suggested, with reason, to remove knowledge of HBV, which of course is a confounding factor. We had forgotten to remove it from the manuscript in the data analysis paragraph. Marital status, history of drug injection and density of residence were also included in the analysis, but were not significantly associated with HBs Ag positivity (see Table 3).

6. The authors did not ask about the history of HB immunization in the study participants. Although most likely did not benefit from vaccination against HBV in childhood as part of EPI, some may receive the vaccine in adult. We therefore can distinguish between who has protective or susceptible for HBV infection.

Thank you for your comment. Unfortunately, students were not asked about their vaccination status. We’ve touched upon this point in the discussion. Line 274–275
