# Supplementary material for: Prevalence of hepatitis B virus infection and its associated factors among students in N’Djamena, Chad
Source: PLoS One. 2024 Apr 18;19(4):e0273589. doi: 10.1371/journal.pone.0273589 (PMC11025733; doi:10.1371/journal.pone.0273589)
Supplement: Attachment — Submitted filename: Response to Reviewers.docx [file pone.0273589.s012.docx]

**Comments to the Author**

I commend the authors did a great job by incorporating reviewers' comments which substantively improved the paper. However, the paper doesn't until now rigorously align with Plos One's authors guidelines. Please note the following:

1. References: Plos One uses brackets;

Thank you for this observation, we've changed hooks

2. In all Tables, add 1 decimal for percentages

Thank you for your comment, we have added 1 decimal for percentages. Table 1: line 185-186, Table 2:199-200 Table 3:212-213

3. When citing R, the date is unnecessary

Thank you, we have deleted. Line 168

Additionally, reviewer #2 has raised some comments to be integrated. I suggest to put these questions in the discussion section or alternatively, add a study limitations section.

Thank you for this suggestion, we have added a section on the Strengths and limitations
