# Supplementary material for: Prevalence of hepatitis B virus infection and its associated factors among students in N’Djamena, Chad
Source: PLoS One. 2024 Apr 18;19(4):e0273589. doi: 10.1371/journal.pone.0273589 (PMC11025733; doi:10.1371/journal.pone.0273589)
Supplement: Attachment — Submitted filename: reponse to reviewer.docx [file pone.0273589.s013.docx]

Response to reviewers

One of the reviewers suggested that final version should incorporate the following changes based on the manuscript evaluation:

"In the submitted manuscript “Prevalence of hepatitis B virus infection and its associated factors among students in N’Djamena, Chad”, PONE-D-22-22361R2, this study addressed the high prevalence of HBV infection among university students in Chad. HBV prevention, especially HBV immunization, should be considered in low-income countries.

However, there are some points that need to be clarified:

1. The authors should mention inclusion criteria in the method part (e.g., sex, age, health status, etc.).

Thank you for this observation. Students who accepted to participate in the study were enrolled consecutively irrespective of age, sex and other sociodemographic characteristics or risk factors line 105-109

2. The data was collected using a questionnaire, was it a self-administered questionnaire or face-to-face interview? Please specify in data collection method.

Thank you for your pertinent observation. The data was it questionnaire face-to-face line 122-123

3. The prevalence of HBV infection was 14.87%, what’s about 95%CI? Please specify.

14.87 is the prevalence of HBsAg in the population studied (95% CI = 13.9-21.7%). Line 193

4. I would suggest using median age instead of mean age.

Thank you for this suggestion. Median age was 24 years. Line 185.

5. Considering p<0.30 in bivariate analysis, why did the authors select only five variables for multivariable logistic regression? (What about age, marital status, knowledge of HBV, history of drug injection, density of residence? there were also p<0.30.)

Age was included in the analysis (age>26 was a protective factor). A previous reviewer and the editor suggested, with reason, to remove knowledge of HBV, which of course is a confounding factor. We have forgotten to remove it from the manuscript in the data analysis paragraph. Marital status, history of drug injection and density of residence were also included in the analysis, but were not significantly associated with HBs Ag positivity (see table 3). Line 215

6. The authors did not ask about the history of HB immunization in the study participants. Although most likely did not benefit from vaccination against HBV in childhood as part of EPI, some may receive the vaccine in adult. We therefore can distinguish between who has protective or susceptible for HBV infection."

Thank you for your comment. Unfortunately, students were not asked about their vaccination status. Please see discussion — line 275—277

I commend the authors did a great job by incorporating reviewers' comments which substantively improved the paper. However, the paper doesn't until now rigorously align with Plos One's authors guidelines. Please note the following:

1. References: Plos One uses brackets;

Thank you for this observation, we've changed hooks

2. In all Tables, add 1 decimal for percentages

Thank you for your comment, we have added 1 decimal for percentages. Table 1: line 188-189, Table 2:203-204 Table 3:214-215

3. When citing R, the date is unnecessary

Thank you, we have deleted. Line 171

Additionally, reviewer #2 has raised some comments to be integrated. I suggest to put these questions in the discussion section or alternatively, add a study limitations section.

Thank you for this suggestion, we have added a section on the Strengths and limitations

I commend the authors did a great job by incorporating reviewers' comments which substantively improved the paper. However, the paper doesn't until now rigorously align with Plos One's authors guidelines. Please note the following:

1. References: Plos One uses brackets;

Thank you for this observation, we've changed hooks

2. In all Tables, add 1 decimal for percentages

Thank you for your comment, we have added 1 decimal for percentages. Table 1: line 188-189, Table 2:203-204 Table 3:214-215

3. When citing R, the date is unnecessary

Thank you, we have deleted. Line 171

Additionally, reviewer #2 has raised some comments to be integrated. I suggest to put these questions in the discussion section or alternatively, add a study limitations section.

Thank you for this suggestion, we have added a section on the Strengths and limitations

However, there are some points that need to be clarified:

1. the authors should mention inclusion criteria in the method part (e.g., sex, age, health status, etc

Thank you for this observation. We have revised the text accordingly, stating that students who accepted to participate in the study were enrolled consecutively irrespective of age, sex, other sociodemographic characteristics or risk factors. Line 105–109.

2. The data was collected using a questionnaire, was it a self-administered questionnaire or face-to-face interview? Please specify in data collection method.

Thank you for your pertinent observation. The interviews were done during face-to-face meetings. Line 122–123.

3. The prevalence of HBV infection was 14.87%, what’s about 95%CI? Please specify.

14.87 is the prevalence of HBsAg in the population studied (95% CI = 13.9–21.7%). This confidence interval was added to the text. Line 195.

4. I would suggest using median age instead of mean age.

Thank you for this suggestion. Median age was 24 years. Line 185.

5. Considering p<0.30 in bivariate analysis, why did the authors select only five variables for multivariable logistic regression? (What about age, marital status, knowledge of HBV, history of drug injection, density of residence? there were also p<0.30.)

Age was included in the analysis (age ≥26 was a protective factor). A previous reviewer and the editor suggested, with reason, to remove knowledge of HBV, which of course is a confounding factor. We had forgotten to remove it from the manuscript in the data analysis paragraph. Marital status, history of drug injection and density of residence were also included in the analysis, but were not significantly associated with HBs Ag positivity (see Table 3).

6. The authors did not ask about the history of HB immunization in the study participants. Although most likely did not benefit from vaccination against HBV in childhood as part of EPI, some may receive the vaccine in adult. We therefore can distinguish between who has protective or susceptible for HBV infection.

Thank you for your comment. Unfortunately, students were not asked about their vaccination status. We’ve touched upon this point in the discussion. Line 274–275
