# Supplementary material for: Prevalence of hepatitis B virus infection and its associated factors among students in N’Djamena, Chad
Source: PLoS One. 2024 Apr 18;19(4):e0273589. doi: 10.1371/journal.pone.0273589 (PMC11025733; doi:10.1371/journal.pone.0273589)
Supplement: Attachment — Submitted filename: reponse to reviewer.docx [file pone.0273589.s015.docx]

**Dear authors, please note the following:**

1. I have extensively edited the manuscripts. Kindly incorporate these changes

We have been able to incorporate changes as requested by the Editor. Line: 18-20, 49, 55-57; 62; 64; 72; 75;95; 97; 116; 134; 142; 199; 230-231; 235; 238;

2. I am a bit concerned with Table 3. In this Table, some variables do not have confidence intervals in "multivariate analyses". I can understand that some variables were not included in multivariate analyses. However, doing this makes the Table a bit cumbersome to understand. I suggest that you separate bivariate Table and multivariate Table (in which ONLY variables retained in multivariate analyses are included. That way, each variable (or categories of variables) will have a confidence interval.

Thank you for this suggestion, we have table 3 in 2 tables. Table 3: line 216 and table 4: 228
