# Supplementary material for: Prevalence of hepatitis B virus infection and its associated factors among students in N’Djamena, Chad
Source: PLoS One. 2024 Apr 18;19(4):e0273589. doi: 10.1371/journal.pone.0273589 (PMC11025733; doi:10.1371/journal.pone.0273589)
Supplement: Attachment — Submitted filename: PONE-D-22-22361 reviewer comments.docx [file pone.0273589.s005.docx]

**Dear Author,**

I am wishing to acknowledge author's work entitled: ‘Prevalence of hepatitis B virus infection and associated risk factors among students in N'Djamena, Chad’. The results illustrated the prevalence of hepatitis B virus among students. Therefore, this study will be useful for future prevention and control of hepatitis infection through the knowledge of prevalence HBV and risk factors among students. The manuscript is more or less in line with the vision, objectives and instructions of PLoS One. Despite its potential, however, this manuscript contains major errors in data presentation and interpretation, grammatical errors which need to be corrected (see the attached file).

**General Comments**

1. Although what authors are reporting is not a novelty, they must however be commended for their efforts in reporting what is prevalence of HBV among students in their locality. But there are so many researches worldwide which were done by more advanced lab techniques like ELISA and molecular techniques (PCR).

2. Use of English language is poor in certain sections and would require a detailed revision.

3. Based on PLoS One authors’ guideline, figures must be uploaded separately in tif or other form.

**Title**

- The title was good. But why you removed the area name ‘Emi Koussi’ from the title? You talked more about this study area in the methods part.

**Abstract**

- The punctuation colon (:) in each subheading was not necessary.
- Line 17; read as ‘Viral hepatitis B (HBV) is a major public health threat’. It is better written as ‘Viral hepatitis B virus (HBV) is a major public health problem’. Here the word ‘threat’ is not the appropriate word.
- Line 20-21; ‘This study aims to determine the prevalence-------------’ It is better written as, ‘This study aimed to determine the prevalence--------------------’
- Line 23; ‘-------- conducted in July 2021 including 457 students randomly selected-------------’. Here a specific length of time should be mentioned. Similarly, which random sampling technique was used? Simple or systematic?
- Which specimen type you have processed? It should be described before you talk about the method.
- Line 26; ‘Descriptive analyzes and binary logistic regressions were used to examine the relationship between dependent variables and sociodemographic including other risk factors’. It is better you say, ‘Descriptive analysis and binary logistic regressions were used to examine the relationship between dependent variables and socio-demographic factors including other risk factors.’
- Among how many study participants the study conducted?
- In the result part, it is better you include both the numerator and denominator together with percentage in presenting prevalence, for example A/B (14.87%).
- Too short and shallow result presentation
- Your result completely contrasts the fact. Are you interpreting the result inversely? See the data, ‘Students aged between 26-35 years (0.032), who were born from HBsAg positive mothers (0.027), who lived with a roommate (0.025) or had a history of hepatitis (p=0.015) were less likely to contract HBV infection’. For example, A participant who lived with a roommate has a probability of acquiring HBV 4.5 times than those living lonely (OR= 5.42, 95% CI: 1.55-25.6; p= 0.025). The author better go through the analysis and correct mistakes that appeared.
- What is your baseline to say ‘high prevalence of HBV infection’?
- Which type of conclusion is it? ‘This study highlights some solutions to improve the situation of hepatitis B in Chad and suggests policies to eliminate viral hepatitis’.
- Additional key words:-HBsAg, risk factor
- It should be re**-**written and make adjustments based on the comments in the methods and results part that follows.

**Introduction**

- The introduction seems like literature review, so it is better modified.
- The word treat is not the appropriate word; please replace by other word.
- The introduction described the burden among clinically ill participants, not apparently healthy individuals (e.g. students). Therefore, the author better show the burden of HBV in similar or related study populations.
- There was no description about the microbiology of HBV.
- I do not think the importance of writing values for P-value, 95%CI and OR. For example, ‘Infants infected during the perinatal period 62 have 3.74 times (OR=3.74, 95% CI: 0.97–14.39) risk of becoming chronic carriers [11]’.
- Line 82-84 should be deleted. ‘Identifying these risk factors for HBsAg 83 carriage with appropriate management would reduce the disease burden. Knowledge of the HBV 84 infection risk factors would also guide policies aimed at reducing the HBV burden’.

**Materials and Methods**

- Line 93; the subheading should be ‘Sample size determination and sampling technique’
- About the sampling technique, please see the comment in the abstract
- The ‘selection of variables’ subheading better be replaced by ‘Socio-demographic data collection’
- The ‘Biological analysis’ also should be replaced by ‘Laboratory data processing’
- In the ethical clearance part, were all participants above the age of 18 years old? If not, Why did not you took assent from participants below the age of 18 years old?
- Your participants were apparently healthy individuals (students). There should be a mechanism in place to link those who need treatment with the healthcare system. How do you manage the psychological and potential social harm on HBV positives that do not need treatment? So ethical issues were not well described.
- Specimen collection and processing needs to be well written.

**Results**

- Your subheading ‘Sample characteristics’ was not the appropriate one. It is better you replace by, ‘ Socio-demographic characteristics’
- Based on your sample size determination your sample size was 422. Why you included 457 in the result part?
- You mix up the socio-demographic characteristics of participants with prevalence of HBV as well as risk factors. I recommend you to present, first the socio-demographic characteristics and then prevalence of HBV, finally risk factors for HBV.
- What is your base to classify the age group? Have you associated with possible risks of interest? It is better again re-group and perform the analysis.
- Is there any difference between the two subheadings? ‘Factors associated with HBV infection among students’ and ‘Prevalence and risk factors associated with HBsAg carriage’. For me it was not clear.
- It was difficult to understand/catch up the concepts of the tables. They lacked OR and 95%CI. Yet you presented in the text. From where you get it (OR and 95%CI)?
- Similarly, it is better you represent the crude odds ratio by COR and the adjusted odds ratio by AOR than simply by OR.
- All table titles should be complete in description, area and period of study.

**Discussions**

- Since the necessity of doing this work was already described in the introduction part. So here writing about it was not necessary. Therefore, delete the following. Line 206 ‘To inform policies aiming to reduce the burden of HBV in Chad and to achieve the objectives of the 207 national health plan 2016-2030, this study was designed to determine the prevalence of HBV and to 208 identify the HBV infection risk factors, among students at the Universities of N'djamena and Emi Koussi’.
- Authors tried to compare their findings with different reports from worldwide. They also better justify the reason for variation among results of different research findings with respect to theirs’ based on actual situation; most of the reason for variation mentioned in the discussion part were already intensively described by other authors.
- Most of the literatures used for comparisons were researches on patients. In this case these literatures were incomparable to this study.

**Conclusions**

- Normally, conclusion emanates from your findings of the research.
- Revise it and accommodate recommendation of community/government importance.
