# Supplementary material for: Prevalence of hepatitis B virus infection and its associated factors among students in N’Djamena, Chad
Source: PLoS One. 2024 Apr 18;19(4):e0273589. doi: 10.1371/journal.pone.0273589 (PMC11025733; doi:10.1371/journal.pone.0273589)
Supplement: Attachment — Submitted filename: PONE-D-22-22361 reviewer comments.doc [file pone.0273589.s007.doc]

**Dear Author,**

I am wishing to acknowledge author's work entitled: ‘Prevalence of hepatitis B virus infection and associated risk factors among students in N'Djamena, Chad’. The results illustrated the prevalence of hepatitis B virus among students. Therefore, this study will be useful for future prevention and control of hepatitis infection through the knowledge of prevalence HBV and risk factors among students. The manuscript is more or less in line with the vision, objectives and instructions of PLoS One. Despite its potential, however, this manuscript contains major errors in data presentation and interpretation, grammatical errors which need to be corrected (see the attached file).

**General Comments**

1. Although what authors are reporting is not a novelty, they must however be commended for their efforts in reporting what is prevalence of HBV among students in their locality. But there are so many researches worldwide which were done by more advanced lab techniques like ELISA and molecular techniques (PCR).

**Our response:**

We thank reviewer for comments, we carried out additional tests by ARCHITECT HBsAg Quantitative confirming the results. Line:158-161

2. Use of English language is poor in certain sections and would require a detailed revision.

**Our response:**

We thank reviewer 1 for comments.

We have answered point by point and correct typo or grammatical errors.

3. Based on PLoS One authors’ guideline, figures must be uploaded separately in tif or other form.

**Our response:**

“Thanks for reminding us about the respect of style requirement of PLOS One related to figures. However, we have not a figure to integrate into this paper”.

**Title**

- The title was good. But why you removed the area name ‘Emi Koussi’ from the title? You talked more about this study area in the methods part.

**Our response:**

Thank you, "Emi Koussi" is the name of one of Universities whereas the study area itself is N'djamena

**Abstract**

- The punctuation colon (:) in each subheading was not necessary.

Thank you for the remark. We have removed it accordingly

- Line 17; read as ‘Viral hepatitis B (HBV) is a major public health threat’. It is better written as ‘Viral hepatitis B virus (HBV) is a major public health problem’. Here the word ‘threat’ is not the appropriate word.

Thank you for mentioning this. We have modified accordingly in -- line 21; "Hepatitis B virus (HBV) is a major public health problem." Line 20.

- Line 20-21; ‘This study aims to determine the prevalence-------------’ It is better written as, ‘This study aimed to determine the prevalence--------------------’

Thanks, in line 23-24 we have replaced ‘This study aims to determine the prevalence -------------’ by ‘This study aimed to determine the prevalence---------------’

- Line 23; ‘------ conducted in July 2021 including 457 students randomly selected--------’. Here a specific length of time should be mentioned. Similarly, which random sampling technique was used? Simple or systematic?
- **Our response:**
- Thanks for the comment. Line 28; ‘-------- conducted in July 2021 including 457 students randomly selected-------------’. Retain " .........from 3 to 23 July 2021. Simple random sampling technique was used to calculate sample size. Participant were not selected randomly but based on a consecutive sampling.
- Which specimen type you have processed? It should be described before you talk about the method.
- The type of sample taken is serum. Line: 29.
- Line 26; ‘Descriptive analyzes and binary logistic regressions were used to examine the relationship between dependent variables and sociodemographic including other risk factors’. It is better you say, ‘Descriptive analysis and binary logistic regressions were used to examine the relationship between dependent variables and socio-demographic factors including other risk factors.’

Thank you for mentioning this. We replaced the sentence as suggested: line 166-167; ‘Descriptive analysis and binary logistic regressions were used to examine the relationship between dependent variables and socio-demographic factors including other risk factors.’

- Among how many study participants the study conducted?
- **Thank you,** the study was conducted among 457 participants. University of N'djamena counts 26349 students.
- In the result part, it is better you include both the numerator and denominator together with percentage in presenting prevalence, for example A/B (14.87%).

Thank you for this comment, but we do not agree with this comment because the tables will be too clumpy.

- Too short and shallow result presentation

Thank you, the result presentation has been revised and we hope that it is more detailed and requested. Line: 208-262.

- Your result completely contrasts the fact. Are you interpreting the result inversely? See the data, ‘Students aged between 26-35 years (0.032), who were born from HBsAg positive mothers (0.027), who lived with a roommate (0.025) or had a history of hepatitis (p=0.015) were less likely to contract HBV infection’. For example, A participant who lived with a roommate has a probability of acquiring HBV 4.5 times than those living lonely (OR= 5.42, 95% CI: 1.55-25.6; p= 0.025). The author better go through the analysis and correct mistakes that appeared.

Thank you, we have reviewed the analysis: “Having mother HBV (AOR: 2.60; 95%CI: 1.18-5.80, p=0.018), having transcutaneous procedure (AOR: 2.63 95%CI: 1.32-5.10, p=0.005), being flat share (AOR: 7.19, 95%CI 2.08-33.79, p=0.004) and having history of HBV screening (AOR: 2.03, 95%CI: 1.11-3.65, p=0.019) were associated with a higher risk of HBV infection” Line: 249-253.

- What is your baseline to say, ‘high prevalence of HBV infection’?

Thank you for this remark. by the way, we would rather say that this prevalence, higher than 8%, places our study population in the high endemicity group according to the WHO classification. We have changed the sentence in the manuscript. Line: 272-273.

- Which type of conclusion is it? ‘This study highlights some solutions to improve the situation of hepatitis B in Chad and suggests policies to eliminate viral hepatitis’.

## Thank you, we have revised the presentation of the conclusion. Line: 367-378

Additional key words: -HBsAg, risk factor

We have added Additional key words: -HBsAg, risk factor. Line 52.

- It should be re**-**written and make adjustments based on the comments in the methods and results part that follows.
- Thanks for your comments, we have revised the whole manuscript and focus on highlighted parts accordingly.

**Introduction**

- The introduction seems like literature review, so it is better modified.

Thanks for your comments. We modified the introduction. Line: 54-107.

- The word treat is not the appropriate word; please replace by other word.

Thank you, we have changed the sentence according to your suggestion.

- The introduction described the burden among clinically ill participants, not apparently healthy individuals (e.g. students). Therefore, the author better show the burden of HBV in similar or related study populations.

Thank you, we have changed the introduction according to your suggestion, by changing some references.

- There was no description about the microbiology of HBV.

Thank you, we have added a paragraph about the microbiology of HBV according to your suggestion. Line 55-58.

- I do not think the importance of writing values for P-value, 95%CI and OR. For example, ‘Infants infected during the perinatal period 62 have 3.74 times (OR=3.74, 95% CI: 0.97–14.39) risk of becoming chronic carriers [11]’.

Thank you, we have taken this remark into account.

- Line 82-84 should be deleted. ‘Identifying these risk factors for HBsAg 83 carriage with appropriate management would reduce the disease burden. Knowledge of the HBV 84 infection risk factors would also guide policies aimed at reducing the HBV burden’.

Thank you, we have deleted this sentence.

**Materials and Methods**

- Line 93; the subheading should be ‘Sample size determination and sampling technique’.

Thank you, we have considered the title ‘Sample size determination and sampling technique’: line 119.

- About the sampling technique, please see the comment in the abstract.
- The ‘selection of variables’ subheading better be replaced by ‘Socio-demographic data collection’

Thank you, we have considered the title ‘Socio-demographic data collection’: line 143.

- The ‘Biological analysis’ also should be replaced by ‘Laboratory data processing’

Thank you, we have considered the title ‘Laboratory data processing’: line 153.

- In the ethical clearance part, were all participants above the age of 18 years old? If not, why did not you take assent from participants below the age of 18 years old?

Thank you, all participants were above the age of 18 years old. Age ranged from 19 to 59 years. Line: 209.

- Your participants were apparently healthy individuals (students). There should be a mechanism in place to link those who need treatment with the healthcare system. How do you manage the psychological and potential social harm on HBV positives that do not need treatment? So ethical issues were not well described.

Thank you for raising this point, HBV-positive people have been referred to the focal point of the fight against hepatitis in Chad for follow-up and advice. Line: 197-201.

- Specimen collection and processing needs to be well written.

Thank you, you pointed out this commend. We proceeded to rewrite the specimen collection. Line 144-152.

**Results**

- Your subheading ‘Sample characteristics’ was not the appropriate one. It is better you replace by, ‘ Socio-demographic characteristics’
- Thank you, we have considered the title ‘Socio-demographic characteristics’: line 207.
- Based on your sample size determination your sample size was 422. Why you included 457 in the result part?

422 is the minimum size of the calculated sample. The sample was distributed proportionally to faculties selected purposely based on the number of students with Faculty of Languages, Letters, Arts and Communication (75 students), Faculty of Educational Sciences (80 students), Faculty of Exact Sciences and Applied (50 students), Faculty of Economics and Management (88 students), Faculty of Human and Social Sciences (75 students) and University of Emi Koussi (50 students). A total of 457 were students included in the study: line 127-134.

- You mix up the socio-demographic characteristics of participants with prevalence of HBV as well as risk factors. I recommend you to present, first the socio-demographic characteristics and then prevalence of HBV, finally risk factors for HBV.

Thank you for this recommendation, we have presented, first the socio-demographic characteristics (table 1) line 219-220, and then prevalence of HBV ( table 2) line 255-256, finally risk factors for HBV( table 3) line 263-264.

- What is your base to classify the age group? Have you associated with possible risks of interest? It is better again re-group and perform the analysis.

Thank you, we have reclassified the Age category: <20, 20 – 30, 30- 40 and > 40, referring to the article “bdoul Rahamane Njigou Mawouma, Amadou Hapsatou Djoulatou, Eliane Ornella Komnang, Etienne Omolomo Kimessoukie. Facteurs associés à l´infection de l´hépatite B chez les femmes enceintes dans les formations sanitaires du district de santé de Mokolo/Région de l´Extrême-Nord Cameroun. **PAMJ. 21 Jan 2022. 41(61)”**

- Is there any difference between the two subheadings? ‘Factors associated with HBV infection among students’ and ‘Prevalence and risk factors associated with HBsAg carriage’. For me it was not clear.

## Thanks, the two subtitles have been rewritten: - Estimated prevalence of HBsAg: line 255-256 and Factors associated with HBV infection among students. line: 263-264.

- It was difficult to understand/catch up the concepts of the tables. They lacked OR and 95%CI. Yet you presented in the text. From where you get it (OR and 95%CI)?

Thank you, we improved the reading of the tables and bring out the odds ratios and their 95% confidence intervals. Line: 263-264.

- Similarly, it is better you represent the crude odds ratio by COR and the adjusted odds ratio by AOR than simply by OR.

Thank you, we have taken into consideration your suggestion, we have represented the crude odds ratio by COR and the adjusted odds ratio by AOR. Line: 263-264.

- All table titles should be complete in description, area and period of study.

Thank you for this advice, we have taken them into account: Table 1**:** Socio-demographic characteristics of study participants (line 219-220), Table 1**:** Estimated prevalence of HBsAg (line255-256) and Table 3: Factors associated with HBV status of study participants (line263-264).

**Discussions**

- Since the necessity of doing this work was already described in the introduction part. So here writing about it was not necessary. Therefore, delete the following. Line 206 ‘To inform policies aiming to reduce the burden of HBV in Chad and to achieve the objectives of the 207 national health plan 2016-2030, this study was designed to determine the prevalence of HBV and to 208 identify the HBV infection risk factors, among students at the Universities of N'djamena and Emi Koussi’.

Thank you, these sentences have been deleted.

- Authors tried to compare their findings with different reports from worldwide. They also better justify the reason for variation among results of different research findings with respect to theirs’ based on actual situation; most of the reason for variation mentioned in the discussion part were already intensively described by other authors.
- Thank you, we have improved the
- discussion
  Most of the literatures used for comparisons were researches on patients. In this case these literatures were incomparable to this study.

Thank you, we rewrote the discussion: line 269-365.

**Conclusions**

- Normally, conclusion emanates from your findings of the research.

Thank you for the remark, the conclusion has been rewritten. Line: 366-378.

- Revise it and accommodate recommendation of community/government importance.

Thank you for the remark, the recommendation has been rewritten. Line: 379-387.
